# Equity in genome sequencing for rare disease diagnosis: a cross-sectional analysis of data from the UK 100,000 Genomes Project

**DOI:** 10.1016/j.ebiom.2026.106452

**Published:** 2026-08-26

**Authors:** Sam Tallman, Loukas Moutsianas, Thuy Nguyen, Yoonsu Cho, Maxine Mackintosh, Dalia Kasperaviciute, Matthew A. Brown, Jamie M. Ellingford, Karoline Kuchenbaecker, Matt J. Silver

**Affiliations:** a Genomics England, London, UK; b Medical Research Council Integrative Epidemiology Unit, University of Bristol, Bristol, UK; c Alan Turing Institute, London, UK; d Department of Medical and Molecular Genetics, King's College London, London, UK; e Division of Evolution, Infection and Genomic Sciences, School of Biological Sciences, Faculty of Biology, Medicine and Health, University of Manchester, Manchester, UK; f Manchester Centre for Genomic Medicine, St Mary's Hospital, Manchester University NHS Foundation Trust, Health Innovation Manchester, Manchester, UK; g Division of Psychiatry, University College London, London, UK; h Department of Non-communicable Disease Epidemiology, London School of Hygiene & Tropical Medicine, London, UK; i Medical Research Council Unit the Gambia at the London School of Hygiene and Tropical Medicine, Serrekunda, the Gambia

**Keywords:** Genomics, Rare disease, Genetic diagnosis, Genetic ancestry, Variant prioritisation, Equity

## Abstract

**Background:**

Genome sequencing has improved rare disease diagnosis and is now part of routine clinical care in the National Health Service in England. Automated prioritisation pipelines narrow millions of variants per patient to a small subset for clinical review, a process that relies on allele frequency resources that do not fully represent human genetic diversity. We assessed ancestry-related differences in variant prioritisation and diagnostic outcomes in patients from the UK 100,000 Genomes Project.

**Methods:**

We analysed 29,405 rare disease probands with genome sequencing and linked clinical outcomes data. We used multivariable regression to assess ancestry-related differences in the number of variants prioritised for clinical review, the proportion of prioritised variants that were recorded as diagnostic, and diagnostic yield. We also evaluated the use of ancestry-stratified allele frequency filters derived from an independent, diverse UK cohort (n = 33,724).

**Findings:**

Compared with the European ancestry group, the East African group had nearly three times more variants prioritised for clinical review (IRR 2.77, 95% CI 2.33–3.29). Other non-European groups also had significantly higher counts. Diagnostic yield was similar across ancestry groups after adjustment (LRT p = 0.1650). Prioritised variants were less likely to be recorded as diagnostic in East African (OR 0.32, 95% CI 0.22–0.46), West African (0.47, 0.39–0.57), South Asian (0.65, 0.58–0.73), and Middle Eastern (0.68, 0.54–0.86) groups. Applying ancestry-stratified allele-frequency filters removed 3.1% of prioritised variants overall—24.3% in the East African group—without loss of diagnostic sensitivity, including 29.5% of recorded VUS in this group.

**Interpretation:**

Differences in the likelihood of prioritised variants being recorded as diagnostic partly reflect limitations of current allele frequency resources, which use broad population groupings that mask within-group diversity. Increased representation of diverse ancestries in reference databases and better estimation of ancestry-appropriate allele frequencies will help reduce inefficiencies and improve equity in variant prioritisation for rare disease diagnosis.

**Funding:**

The UK Department of Health and Social Care and the EU's Horizon 2020 Research and Innovation Programme.

**Research in context**

**Evidence before this study**

We searched PubMed for studies on rare disease genomics and ancestry-related disparities in variant prioritisation and genetic diagnosis, published from database inception to April 1, 2025, using combinations of terms including “rare disease”, “sequencing”, “variant interpretation”, “variant prioritisation”, “diagnostic yield”, and “genetic ancestry”, with iterative refinement of search strategies. Evidence consistently highlighted under-representation of non-European ancestries in genomic databases and its effect on how rare variants are prioritised and clinically assessed. For example, a 2016 study using exome sequencing found that, after filtering variants against the largest reference databases available at the time, individuals of non-European ancestry were left with longer candidate variant lists. Since then, reference databases have grown considerably in size and diversity, improving the ability to identify variants underlying genetic diagnoses in diverse populations. However, reviews have highlighted that much of global diversity remains under-represented in currently available databases. Studies examining ancestry-related disparities in clinical outcomes in rare disease sequencing cohorts have reported mixed findings. A large 2023 UK-based study of developmental disorders found lower rates of genetic diagnosis among patients of African ancestry, whereas a 2024 study in a smaller US cohort of paediatric and prenatal conditions reported no ancestry-related differences in genetic diagnosis rates.

**Added value of this study**

This study provides a large-scale evaluation of ancestry-related differences in the performance of an automated variant prioritisation pipeline used to support genetic diagnosis in more than 29,000 patients with rare diseases in the UK 100,000 Genomes Project. Participants of non-European ancestries had significantly more variants prioritised for manual review, and these variants were less likely to be assessed as diagnostic. Disparities were most pronounced in participants of East African ancestry. Filtering variants against a diverse UK-based reference cohort that included a small East African ancestry-specific panel reduced the number of prioritised variants in this group by almost a quarter.

**Implications of all the available evidence**

These findings highlight the continued need for broader and more detailed representation of genetic diversity in genomic reference databases, particularly within communities of African ancestries where genetic variation remains under-represented or homogenised. Incorporating more representative reference data into variant prioritisation approaches could reduce unnecessary manual review and improve the efficiency of genetic diagnosis as genome sequencing for patients with rare diseases becomes more widely implemented in routine healthcare in the UK and worldwide.

## Introduction

Over 400 million people worldwide are estimated to be living with a rare disease.^1^ While it is thought that more than 80% of rare diseases have a genetic component, most patients do not receive a genetic diagnosis after standard testing.^2^ By capturing the majority of the genetic variation in an individual, genome sequencing has improved genetic diagnosis rates.^3^ However, distinguishing causal pathogenic variants from the millions of benign variants present in the genome remains a key challenge.

The rare diseases arm of the UK 100,000 Genomes Project (100kGP), is the largest study of sequenced rare disease patients and family members to date.^4^ As part of this nation-wide programme, variants identified through genome sequencing and prioritised by Genomics England's^5^ automated variant prioritisation pipeline have aided in the identification of causal pathogenic variants in thousands of previously undiagnosed participants. The project laid the foundation for the introduction of genome sequencing for patients with rare disease into the National Health Service (NHS) in England as part of routine clinical care.^6^

Pathogenic variants causing rare diseases are generally expected to be rare in all populations.^7^ Allele frequency estimates from large-scale reference datasets such as the Genome Aggregation Database (gnomAD)^8^ are therefore used to assist in variant prioritisation by excluding variants too common to plausibly cause the disease. However, a large fraction of global genetic diversity remains to be surveyed and there continues to be an overrepresentation of European ancestries in existing reference datasets.^9^ As genomic testing for rare disease diagnosis is incorporated into the NHS and other healthcare systems across the world, it is vital to ensure that advances in genomic medicine translate into accurate and equitable care for all.

We investigated how genetic ancestry affected the performance of Genomics England's rare disease variant prioritisation pipeline in participants from the 100kGP.

## Methods

### Study design

We analysed genome sequencing and linked health data from participants with rare diseases recruited into the 100kGP. Recruitment to the rare disease programme was coordinated by Genomics England in partnership with 16 NHS Genomic Medicine Centres (GMCs) across the UK between 2015 and 2018, with sequencing completed by December 2018 and results from the automated variant prioritisation pipeline (see “Procedures”) returned to NHS clinical laboratories by July 2019. Study protocols for the 100kGP are available online.

This retrospective analysis was restricted to data included in the version 19 main programme release of the National Genomic Research Library (NGRL),^5^ a research database comprising genome sequencing data and NHS health records including participants in the 100kGP. Patient representatives were not involved in the design or analysis of this study.

### Ethics

Ethical approval for the 100kGP was granted by the East of England–Cambridge South Research Ethics Committee (Reference:14/EE/1112; IRAS project ID 166046). Analyses were conducted within the secure Genomics England Research Environment under project reference RR786, approved through the Genomics England Research Registry. All participants consented to undergo genome sequencing, receive findings relevant to their condition and permitted access to their de-identified genomic and clinical data for research. This manuscript adheres to the 2024 Declaration of Helsinki ethical principles.

### Patients

Patients in the 100kGP rare disease programme were recruited across a broad spectrum of rare conditions (defined as <1 in 2000 births^10^). NHS medical practitioners determined eligibility based on suspicion of a monogenic or oligogenic disorder without a genetic diagnosis after standard investigations. Where possible, biological parents or other relatives were enrolled to support family-based analyses. Phenotypic data were collected at enrolment using terms selected from curated disease-specific data models, supporting the clinician-led classification of probands and affected relatives into standardised disease groups for downstream analysis (Supplementary Table S1). Demographic information including year of birth, sex, and self-reported ethnicity was collected alongside clinical data.

### Procedures

**Fig. 1:**
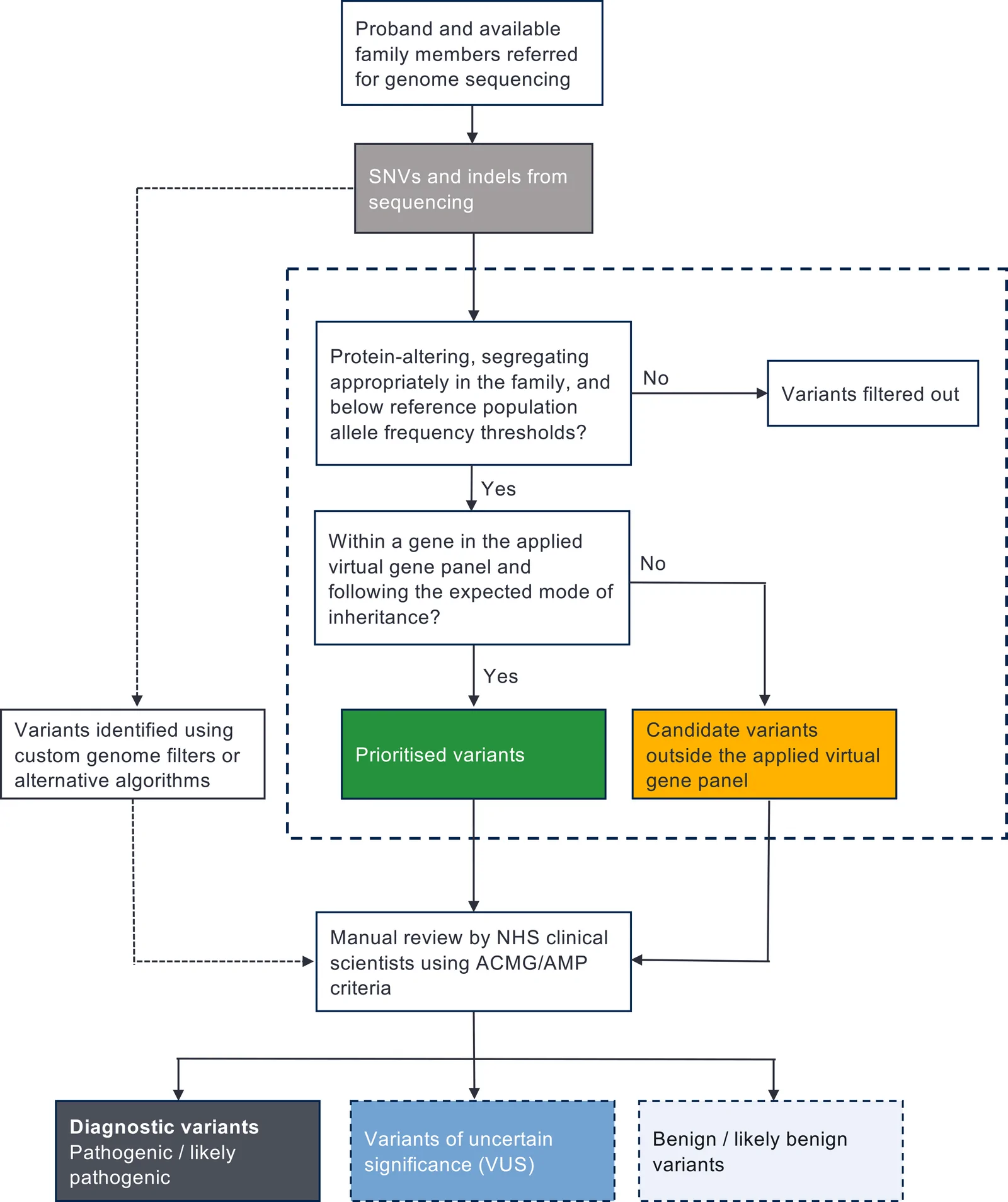
Schematic of the automated variant prioritisation pipeline and manual review process Single nucleotide variants (SNVs) and small indels were evaluated by Genomics England's automated variant prioritisation pipeline (dashed box). Variants were first filtered by predicted protein-altering consequence, familial segregation (where family data were available), and reference population allele frequency thresholds. Further filtering was based on membership of an applied, disease-relevant virtual gene panel and the expected mode of inheritance. *Prioritised variants* passing all the above criteria (green box) were sent for review by NHS clinical scientists. *Candidate variants* outside the applied virtual gene panel (orange box) were also sent but not routinely reviewed. Additional variants could also be identified for review using custom filters or alternative algorithms (dashed arrow). Following interpretation, clinical scientists recorded all *diagnostic variants* that fully or partially explained the proband's phenotype(s) (black box) in outcomes questionnaires. Non-diagnostic variants, such as *variants of uncertain significance (VUS*, blue box*)* or benign/likely benign variants (light grey box), could also be recorded but this was not mandatory.

As part of the 100kGP's primary clinical findings pathway for rare disease, single nucleotide variants (SNVs) and small insertions or deletions (indels) identified through genome sequencing of probands and available family members were processed via Genomics England's automated variant prioritisation pipeline. Full documentation is available online and a schematic overview is provided in Fig. 1.

The pipeline aimed to identify potentially causal variants by filtering out variants highly unlikely to cause a rare disease, before prioritising those most likely to explain the proband's phenotype(s). Filtering retained only high quality (PASS), predicted protein-altering SNVs and indels that segregated appropriately in the family or that showed strong evidence of being de novo and that occurred at <0.1% frequency (under dominant models) or <1% (under recessive models) in external reference datasets (gnomADv2^8^ and 1000 Genomes^11^) and sequencing technology-matched controls (see Supplementary Table S2 for the applied population frequency filters). Variants passing these criteria were then prioritised if they affected disease-relevant genes and were consistent with the gene's expected mode(s) of inheritance, via the application of expert-assessed virtual gene panels^12^ (continuously updated throughout the programme) selected for the proband's disease group(s) and clinical presentation. These are referred to as ‘prioritised variants’ hereafter.

All prioritised variants were reviewed by NHS clinical scientists at the GMCs using American College of Medical Genetics and Genomics and Association of Molecular Pathology (ACMG/AMP) criteria,^13^ following best-practice guidelines from the Association for Clinical Genomic Science (ACGS).^14^ Clinical scientists also had the option to extend their review beyond prioritised variants. This included candidate variants that passed the pipeline's filtering criteria but fell outside the applied virtual gene panels, as well as variants identified through custom filters or alternative algorithms such as Exomiser^15^ (see Supplementary Appendix (p5) for further details). For each case, Genomics England collected outcomes from the manual review process through questionnaires submitted by the clinical scientists. These questionnaires recorded whether any of the reviewed variants fully or partially explained the proband's presenting phenotype(s), and the pathogenic or likely pathogenic variants on which that conclusion was based, hereafter termed ‘diagnostic variants’ (Fig. 1). Reviewed variants not accepted as diagnostic, including those classified as benign, likely benign or of uncertain significance (VUS), could also be recorded but this was not mandatory.

For this study, we used genome sequencing data to assign each proband to one of eight genetically inferred ancestry groups using a reference dataset curated from UK Biobank,^16^ selected for its broad representation of genetic diversity across the UK population. Full methodological details are provided in the Supplementary Appendix (p7). Briefly, proband genotypes at 55,706 high-quality, linkage disequilibrium-pruned SNVs were projected onto principal components derived from 21 reference groups using *bigsnpr*^17^ (version 1.8.1), and assignments were based on genetic similarity to these reference groups in principal component space. Overlapping reference groups were subsequently merged using a graph-based clustering approach, while very small groups (n < 100) and individuals not assigned to any reference group were combined into a “remaining participants” category, yielding eight final groups (Supplementary Table S3). We use *ancestry group* throughout this Article to refer to these genetic similarity-based groupings, whilst acknowledging that these are simplifications of complex, continuous patterns of genetic ancestry^18^ and that no individuals derive from a single ancestry in any meaningful sense.

Allele frequency estimates in external reference datasets (gnomADv2,^8^ 1000 Genomes^11^) used by the variant prioritisation pipeline are stratified by broad continental population groupings (e.g., ‘African’) that do not fully capture African genetic diversity^19^ or that of African diaspora communities in the UK. To explore the potential benefits of using more representative allele frequency estimates, we tested the application of additional allele frequency filters, stratified by the same eight ancestry groups described above (which include distinct East, West, and North African groups; Supplementary Table S3). These were derived from a UK cohort of 33,724 genetically unrelated individuals (exclusion of all 3rd degree relatives and above; Supplementary Table S4) recruited separately for the COVID-19 Genomics Study,^20^ that were not enriched for rare diseases. Dominant and recessive filtering allele frequencies were adjusted for group-specific sample sizes^21^ (Supplementary Table S5). We also evaluated additional filters derived from the latest gnomAD release (v4.1; Supplementary Table S6) comprising 730,947 exomes and 76,215 genomes, which includes a single ‘African/African American’ reference grouping. Further methodological details are provided in the Supplementary Appendix (p9).

Note, these additional analyses were conducted retrospectively for research purposes only. Results were not returned to NHS clinical teams and did not influence clinical reporting or diagnostic decisions.

### Statistics

From the broader cohort of 35,052 probands, we excluded 3543 whose genome sequencing data were aligned to the earlier GRCh37 reference build; 498 without pipeline results or outcome questionnaires available in the NGRL; and 606 whose questionnaires indicated a fully or partially solved case, but with no recorded diagnostic SNVs or indels, such as those based solely on structural variants or chromosomal abnormalities. This left 29,405 probands for downstream analysis. Sample size estimation, randomisation, and blinding were not performed in this analysis.

We used negative binomial mixed-effects regression to compare the number of prioritised variants across ancestry groups (with all unassigned probands grouped under *remaining participants*), accounting for overdispersion and including random intercepts for the proband's disease group and handling GMC. Probands with more than one clinically indicated disease group were grouped under *Multiple disorders*. Prespecified covariates, selected based on clinical relevance and prior evidence, included age at recruitment, sex, predicted consanguinity, family structure, and family affection status (Table 1). Probands for whom family member disease status was unavailable (2750 probands [9.3%]) were grouped as *unknown* and included in all models.

**Table 1:** Clinical and demographic characteristics for 29,405 analysed probands from the 100kGP rare diseases programme stratified by eight genetically inferred ancestry groups and remaining participants.

	European	East Asian	Middle Eastern	Ashkenazi	South Asian	North African	East African	West African	Remaining participants
**n (% of total)**	23,623 (80.3%)	124 (0.04%)	452 (1.5%)	250 (0.09%)	3030 (10.3%)	147 (0.4%)	159 (0.5%)	679 (2.3%)	941 (3.2%)
**Age at recruitment**									
Median (IQR)	26 (8–50)	30 (10–43)	26 (8–47)	51 (27–64)	14 (6–35)	17 (7–40)	20 (9–40)	33 (12–50)	13 (6–35)
**Reported sex**									
Female, n (%)	11,542 (48.9%)	63 (50.8%)	181 (40.0%)	130 (52.0%)	1319 (43.5%)	71 (48.3%)	64 (40.3%)	344 (50.7%)	419 (44.5%)
Male, n (%)	12,081 (51.1%)	61 (49.2%)	271 (60.0%)	120 (48.0%)	1711 (56.5%)	76 (51.7%)	95 (59.7%)	335 (49.3%)	522 (55.5%)
**Family structure**									
Singleton, n (%)	9303 (39.4%)	69 (55.6%)	199 (44.0%)	159 (63.6%)	939 (31.0%)	54 (36.7%)	70 (44.0%)	373 (54.9%)	288 (30.6%)
Family duo, n (%)	3363 (14.2%)	9 (7.3%)	45 (10.0%)	12 (4.8%)	399 (13.2%)	23 (15.6%)	30 (18.9%)	133 (19.6%)	199 (21.1%)
Family trio, n (%)	8018 (33.9%)	33 (26.6%)	143 (31.6%)	47 (18.8%)	1189 (39.2%)	49 (33.3%)	39 (24.5%)	121 (17.8%)	355 (37.7%)
Other^a^, n (%)	2939 (12.4%)	13 (10.5%)	65 (14.4%)	32 (12.8%)	503 (16.6%)	21 (14.3%)	20 (12.6%)	52 (7.7%)	99 (10.5%)
**Family disease affection**									
Relative affected, n (%)	6365 (26.9%)	33 (26.6%)	134 (29.6%)	81 (32.4%)	828 (27.3%)	44 (29.9%)	43 (27.0%)	166 (24.4%)	214 (22.7%)
Only proband affected, n (%)	14,901 (63.1%)	75 (60.5%)	286 (63.3%)	122 (48.8%)	2038 (67.3%)	93 (63.3%)	103 (64.8%)	454 (66.9%)	667 (70.9%)
Unknown, n (%)	2357 (10.0%)	16 (12.9%)	32 (7.1%)	47 (18.8%)	164 (5.4%)	10 (6.8%)	13 (8.2%)	59 (8.7%)	60 (6.4%)
**Consanguinity**^b^									
Consanguineous, n (%)	136 (0.6%)	<5	156 (34.5%)	<5	1176 (38.8%)	46 (31.3%)	29 (18.2%)	<5	21 (2.2%)
Possibly consanguineous, n (%)	45 (0.2%)	<5	41 (9.1%)	<5	200 (6.6%)	14 (9.5%)	11 (6.9%)	<5	12 (1.3%)
Nonconsanguineous, n (%)	23,442 (99.2%)	122 (98.4%)	255 (56.4%)	247 (98.8%)	1654 (54.6%)	87 (59.2%)	119 (74.8%)	676 (99.6%)	908 (96.5%)

IQR, interquartile range.

a Other family structures include larger family structures or those including non-parental relatives.

b Consanguinity was inferred using the fraction of the genome in long runs-of-homozygosity (ROH).^22^ Categories with less than five individuals (<5) are masked in line with Genomics England reporting practices.

We used logistic mixed-effects regression to compare ancestry group differences in three measures derived from the clinical scientist outcomes questionnaires. Models included random intercepts for disease group and the handling GMC and were adjusted for the same covariates listed above (Table 1). The measures were: (1) diagnostic yield (fully or partially solved case recorded vs none); (2) the proportion of prioritised variants that were recorded as diagnostic; and (3) the proportion of diagnostic variants that were prioritised (the denominator here included all recorded diagnostic variants, including those identified through extended review beyond the prioritised set). For brevity, we refer to these two variant-level measures as the positive predictive value (PPV) and sensitivity of prioritisation, respectively. These should be interpreted as operational measures of pipeline performance rather than standard diagnostic test-level metrics; in particular, “sensitivity” is conditional on known diagnostic variants and does not represent sensitivity with respect to all underlying causal variants, many of which may remain undiscovered.

Likelihood ratio tests (LRTs) assessed the overall significance of ancestry group by comparing multivariable models with ancestry group as a fixed effect against baseline models excluding this term. Adjusted incidence rate ratios (IRRs; for negative binomial models) and adjusted odds ratios (ORs; for logistic models) are reported with 95% confidence intervals (CIs), using the European ancestry group as reference. p values for individual ancestry-group coefficients, using the European ancestry group as reference, were obtained from Wald z tests of model regression coefficients.

Sensitivity analyses compared the PPV and sensitivity of prioritised variants using virtual gene panels applied at the time of sequencing to those using updated versions from January 2024. Stratified analyses examined ancestry group differences across variant consequence categories and inheritance models, as annotated by the variant prioritisation pipeline. Additional robustness analyses repeated the primary multivariable models after excluding the most peripheral 20% of individuals within each ancestry group in principal component space, and after balanced down sampling of each ancestry group to the size of the smallest included group (n = 124) across 500 iterations. Recorded VUS frequency across ancestry groups was also analysed using logistic mixed-effects regression. Full details of these analyses are in the Supplementary Appendix (p11–13).

All tests were two-sided, with p < 0.05 denoting statistical significance. Regression analyses were performed in R (version 4.1.1) using glmmTMB^23^ (version 1.1.2.3).

### Role of funders

Funders were not involved in study design, data collection, data analysis, interpretation or writing of the report.

## Results

We analysed 29,405 probands from the 100kGP rare disease programme (Methods). Disorders spanned 218 diseases across 20 broad disease groups. The most common group was neurodevelopmental disorders with 12,003 participants (40.8%) (Supplementary Table S1). Self-reported ethnic group representation broadly matched census data for England (Office of National Statistics 2021) given the cohort's age profile (Supplementary Appendix p6; Supplementary Figure S1).

Participants were assigned to one of eight genetically inferred ancestry groups to capture broad differences in genetic diversity within the cohort (Methods; Supplementary Table S3; Supplementary Figures S2–S6). Those not confidently assigned to any of these eight groups are labelled ‘remaining participants’. Ancestry group assignments and clinical and demographic characteristics included as covariates in downstream multivariable regression models (Methods) are summarised in Table 1.

Across the cohort, a total of 56,642 SNVs and indels were prioritised by the automated variant prioritisation pipeline (Methods) and returned to NHS clinical scientists for manual review (Fig. 1). A median of one prioritised variant was returned per proband (interquartile range [IQR] 0–2) with none returned for 11,615 probands (39.5%) (Supplementary Table S7; Supplementary Figure S7).

**Fig. 2:**
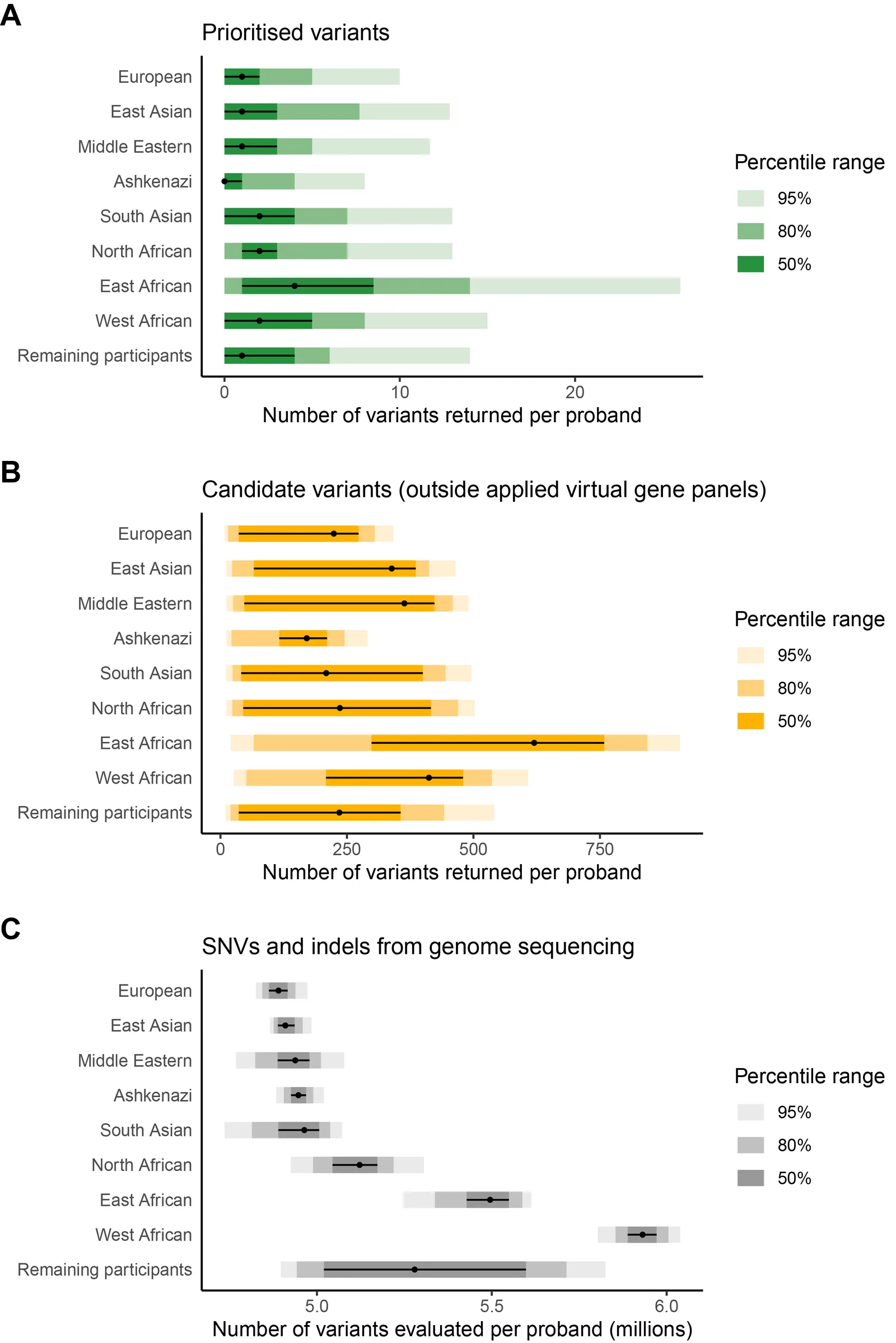
Distribution of variant counts per proband stratified by genetically inferred ancestry group. (**A**) Prioritised variants (green). (**B**) Candidate variants returned by the automated pipeline but appearing outside the applied virtual gene panels (orange). (**C**) Total numbers of SNVs and indels from genome sequencing evaluated by the pipeline (grey). Black dots indicate the median per–proband count and black horizontal lines the interquartile range. Shaded bars represent 50th, 80th and 95th percentile ranges. Summary statistics are reported in Supplementary Table S7.

The number of prioritised variants returned across ancestry groups ranged from a median of 0 (IQR 0–1) in the Ashkenazi group to 4 (IQR 1–8) in the East African group (Fig. 2A). In multivariable negative-binomial regression, ancestry group remained an independent predictor of prioritised variant counts (LRT χ^2^_8_ = 492; p < 0.0001). Compared to the European ancestry group, the adjusted IRR for prioritised variants for the Ashkenazi group was 0.75 (95% CI 0.63–0.89; multivariable negative binomial GLMM, Wald z-test on regression coefficients p = 0.0010), 2.77 (95% CI 2.33–3.29; p < 0.0001) for the East African group, and 1.29–1.79 (all p < 0.0015) for all other ancestry groups (Supplementary Table S8). These differences persisted when analysing 5.3 million candidate variants returned outside of the disease-relevant virtual gene panels (LRT χ^2^_8_ = 2684; p < 0.0001; Figs. 1 and 2B) and were not explained by differences in the number of SNVs and indels evaluated by the pipeline (Fig. 2C). In stratified analyses by variant consequence types and inheritance patterns, missense variants prioritised under inherited dominant models emerged as the principal driver of ancestry group differences (Supplementary Figures S8 and S9).

Of the probands analysed, 5662 (19.3%) received a full or partial genetic diagnosis based on SNVs or indels, regardless of whether the variants were prioritised by the automated pipeline or identified through alternative routes. Diagnostic yield varied from 18.0% (122/679 probands) in the West African ancestry group to 29.0% (36/124) in the East Asian ancestry group, with 18.3% (4335/23,623) in the European ancestry group (full results are shown in Supplementary Table S9). In multivariable logistic regression models, ancestry group was not independently associated with diagnostic yield (LRT χ^2^_8_ = 11.7; p = 0.1650). In this adjusted model, higher diagnostic yield was associated with younger age, male sex, consanguinity, availability of family trio data, and having an affected relative. Full effect estimates are given in Supplementary Table S10.

**Fig. 3:**
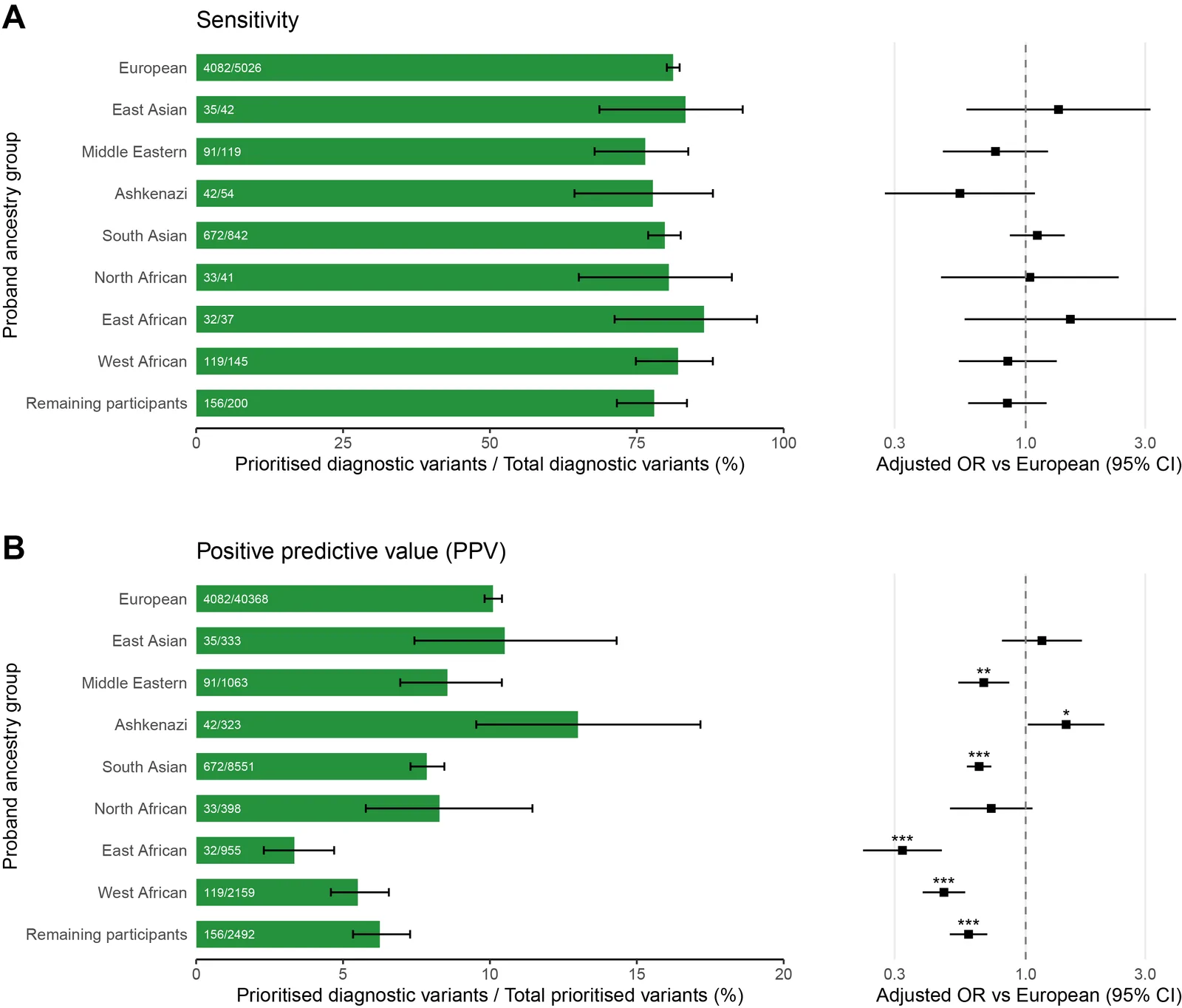
Sensitivity and positive predictive value of variants prioritised by the pipeline stratified by genetically inferred ancestry group. **(A)** Left: proportion of all recorded diagnostic SNVs and indels resulting from prioritised variants (sensitivity) shown as green bars with numerator/denominator labels inside each bar and 95% Clopper–Pearson CIs (whiskers). Right: adjusted-OR forest plot. **(B)** Left: proportion of prioritised variants recorded as diagnostic (PPV) shown as green bars with numerator/denominator labels inside each bar and 95% Clopper–Pearson CIs (whiskers). Right: adjusted-OR forest plot. Estimates of adjusted OR vs European are from multivariable mixed-effects logistic regression models adjusted for age, sex, family structure, family-member affection status, and predicted consanguinity, with random intercepts for disease group and the handling GMC. ∗∗∗p < 0.001; ∗∗p < 0.01; ∗p < 0.05 (Wald z test on regression coefficients). Full regression coefficients, 95% CIs, and p values for all covariates are provided in the Supplementary Tables S11 and S12.

Of 6506 diagnostic SNVs and indels recorded by clinical scientists, 5262 were among the 56,642 prioritised variants identified across the cohort, yielding an overall sensitivity of 80.9% (5262/6506) and a PPV of 9.3% (5262/56,642). Sensitivity of prioritised variants was broadly consistent across ancestry groups (range: 91/119 [76.5%] to 32/37 [86.2%]; LRT χ^2^_8_ = 8.4; p = 0.395) (Fig. 3A; Supplementary Table S11), but PPV varied markedly, ranging from 3.4% (32/955) in the East African group to 13.0% (42/323) in the Ashkenazi group (Fig. 3B left). Ancestry group remained an independent predictor of PPV in multivariable logistic regression models (LRT χ^2^_8_ = 189; p < 0.0001). Compared to the European ancestry group, the adjusted OR of a prioritised variant being diagnostic was lower in the East African (0.32; 95% CI 0.22–0.46; multivariable logistic GLMM, Wald z-test on regression coefficients p < 0.0001); West African (0.47; 0.39–0.57; p < 0.0001); Middle Eastern (0.68; 0.54–0.86; p < 0.0001); South Asian (0.65; 0.58–0.73; p < 0.0001); and remaining participant (0.59; 0.50–0.70; p < 0.0001) groups and higher in the Ashkenazi group (1.45; 1.02–2.06; p = 0.0382) (Fig. 3B right; Supplementary Table S12).

The distribution of inheritance patterns among prioritised diagnostic variants varied across ancestry groups, with higher proportions of recessive homozygous variants observed in groups with greater inferred consanguinity (Table 1; Supplementary Figure S10), consistent with increased autozygosity.

Overall, 4036 variants were classified as variants of uncertain significance (VUS), with at least one VUS recorded in 9.8% (2889/29,405) of probands. As VUS were not systematically recorded by clinical scientists in the outcomes questionnaire, these estimates should be interpreted cautiously. In multivariable logistic regression models, ancestry group remained associated with the probability of having at least one recorded VUS (LRT χ^2^_8_ = 38.8; p < 0.0001), with higher odds of at least one recorded VUS in several non-European ancestry groups (Supplementary Table S13; Supplementary Figure S11).

**Fig. 4:**
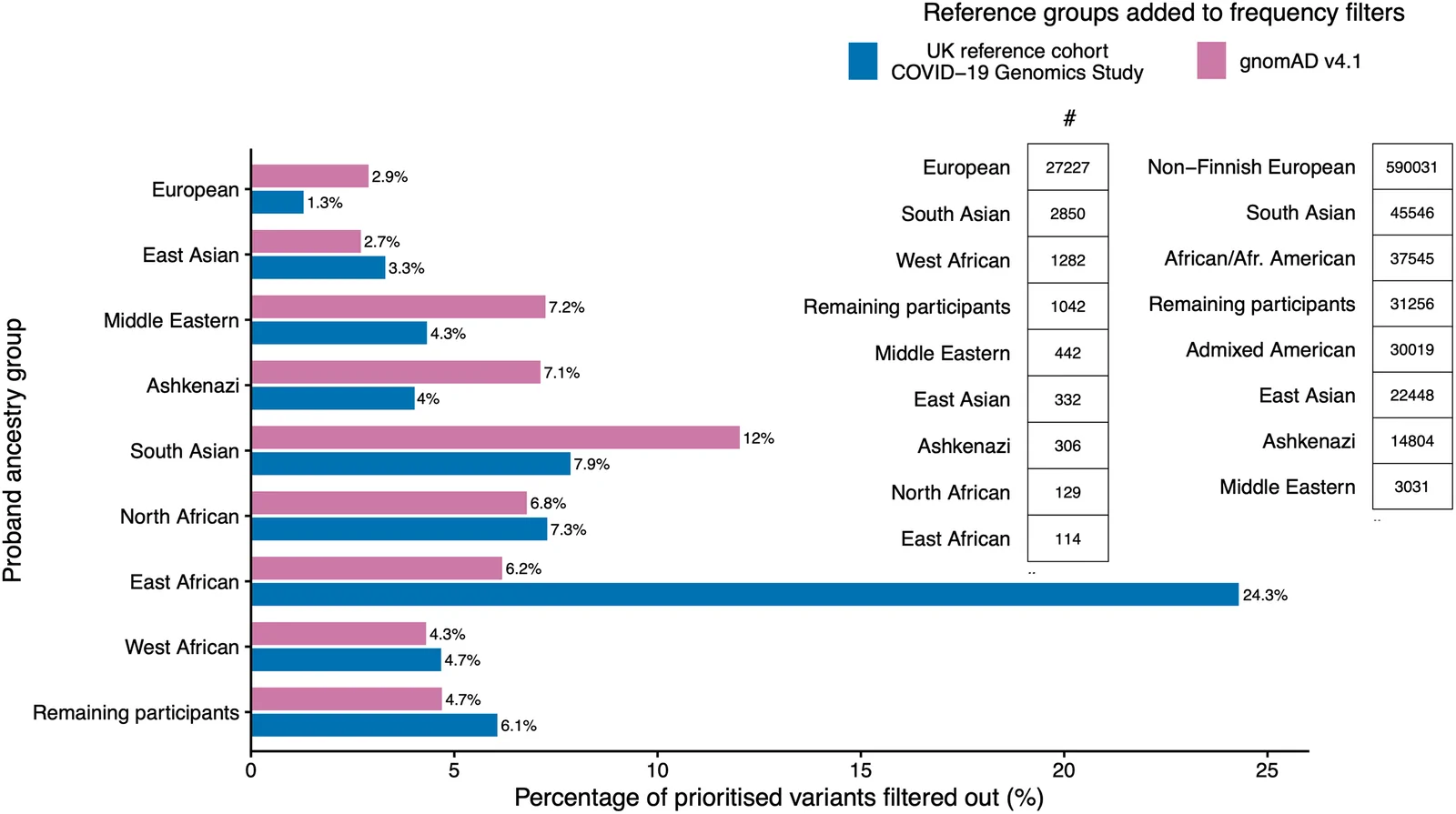
Impact of additional allele frequency filters on prioritised variants across genetically inferred ancestry groups. Blue bars show the percentage of prioritised variants removed in each proband ancestry group when additional allele frequency thresholds estimated from the UK COVID-19 Genomics Study (blue) were added to the automated pipeline filters (Methods). Magenta bars show the same for allele frequency thresholds derived from gnomADv4.1 (magenta). Sample sizes for each labelled group used in allele frequency estimation are in the inset tables. Full filter definitions including sample-size adjustments are shown in Supplementary Tables S5 and S6 Filtering summary statistics are provided in Supplementary Table S14.

We next assessed whether ancestry-stratified allele frequency estimates that reflect the genetic diversity of the 100kGP cohort could improve the PPV of prioritised variants. We re-ran the variant prioritisation pipeline using additional allele frequency filters derived from a relatively small number (33,724) of diverse genomes from the UK COVID-19 Genomics Study,^20^ stratified by the same eight ancestry groups considered throughout our analysis (Fig. 4 blue bars; Supplementary Table S5; Supplementary Figures S12–S18). This removed 3.1% of previously prioritised variants (1777/56,642) without loss of sensitivity (5262/5262, i.e. 100% of diagnostic variants retained). The largest effect was in the East African ancestry group (24.3% [232/955] of prioritised variants removed) and the smallest effect was in the European ancestry group (1.3% [522/40,368] removed). Other ancestry groups saw 3.3% (11/333) to 7.9% (672/8551) of variants removed (Fig. 4; Supplementary Table S14).

When substituting these frequency filters with those from gnomADv4.1,^8^ a substantially larger dataset that lacks an East African ancestry-specific reference group (Fig. 4 magenta bars; Supplementary Table S6), 2.7% (9/333) to 12.0% (1028/8551) of prioritised variants were removed across ancestry groups (Supplementary Table S14). Differences relative to the UK COVID-19 Genomics Study allele frequency filters were modest in most groups (4.1% fewer to 1.4% more removed), but markedly greater in the East African group, where 18.1% fewer prioritised variants were removed. Similar patterns were observed for candidate variants outside the applied virtual panels (Supplementary Figure S19).

46 VUS recorded in the outcome questionnaires were among those filtered out using the ancestry-stratified frequency thresholds from the UK COVID-19 Genomics Study (full list in Supplementary Table S15). 13 of these were identified in probands in the East African ancestry group; 29.5% (13/44) of all recorded VUS in this group. For example, a missense variant (13-114324819-A-G) in *CHAMP1*, initially prioritised under a dominant inheritance model in a proband with intellectual disability and developmental delay, was absent from gnomADv2, and reached a maximum frequency in gnomADv4 of 0.05% (3 of 6084 alleles) in the Middle Eastern group. It was also absent in other groups in the UK COVID-19 Genomics Study cohort but reached a frequency of 2.2% (5 of 228 alleles) in the East African group (Supplementary Table S15). Given the typically de novo and early-onset nature of *CHAMP1*-related disorders,^24^ this high frequency likely exceeds the maximum credible allele frequency for pathogenicity, enabling the use of evidence within the current clinical guidelines^14^ to support a benign classification (BS1).

## Discussion

Genome sequencing is increasingly used in the healthcare pathway for patients with rare diseases as a method to inform diagnosis, prognosis and treatment. The accuracy and precision of genomic approaches for rare disease diagnosis depends on the reliable identification and prioritisation of variants underpinning these conditions. In our analysis of data from the 100kGP, the world's largest cohort of rare disease patients with genome sequencing and linked clinical data, we demonstrate that genetic ancestry affects the performance of an automated variant prioritisation pipeline used to support genetic diagnosis. While diagnostic yield did not differ significantly by ancestry group in multivariable models, we observed significant disparities in the proportion of prioritised variants recorded as diagnostic following clinical review.

These disparities were most pronounced among patients of genetically inferred East African ancestry. For patients in this group, variants prioritised for clinical review were three times less likely to be accepted as diagnostic compared to those in the European ancestry group. Our findings suggest these discrepancies are partly driven by gaps in ancestry-specific allele frequency data in commonly used reference databases such as gnomAD, which aggregate across broad, continental categories that do not adequately capture African genetic diversity.^19^ This means that variants common in individuals with East African ancestries are more likely to appear superficially rare, increasing their likelihood of being prioritised and reducing the likelihood that any given prioritised variant will contribute to a diagnosis. Indeed, when we retrospectively applied new allele frequency filters, including those derived from a small East African ancestry-specific reference group (n = 114), we found that 24.3% of prioritised variants in this group could have been excluded from manual review without loss of diagnostic sensitivity. Generating larger, ancestry-matched allele frequency panels for such under-represented groups using cohorts such as UK Biobank^25^ could further improve resolution of allele frequency estimates, with federated approaches offering a practical route to cross-environment harmonisation.^26^

Previous studies have reported lower diagnostic yields for rare disease patients with non-European ancestries,^27^ whilst other studies have reported no significant ancestry-related differences.^28^ Despite observed differences in the number of variants prioritised and returned for clinical review, we found no evidence that genetic ancestry influenced the likelihood of obtaining a genetic diagnosis in our cohort after adjustment for clinical and demographic covariates. This may reflect the robustness of the manual variant review processes. However, we caution against overinterpreting this finding as the variance in diagnostic yield across the diverse spectrum of disease phenotypes represented in the cohort is large,^2^ and patient numbers for many conditions are small. Larger, more diverse rare disease cohorts, including the addition of data from patients referred for genome sequencing by the NHS Genomic Medicine Service,^6^ may provide the necessary statistical power to identify ancestry-related differences where they exist.

Our study has several limitations. Firstly, the genetically inferred ancestry groups used here do not fully capture the complex, continuous nature of genetic ancestry.^18^ This is particularly relevant for individuals classified as ‘remaining participants’, who do not map to any one ancestry group under our assignment criteria. Differences in variant prioritisation within remaining participants motivate the need for future analyses based on local ancestry or continuous genetic similarity. Furthermore, diagnostic outcomes may be influenced by unmodelled sociocultural, clinical, and environmental factors^27^ that could be correlated with these ancestry groups. Reported differences, or lack thereof, in diagnostic outcomes should be interpreted in this context. The predominance of patients of European ancestry and the wide range of rare disease phenotypes in the 100kGP also limit the ability to detect possible disease-specific ancestry biases. Moreover, recording of VUS in the outcomes questionnaires was not mandatory and may have varied between centres, so VUS counts may be under-ascertained and should be interpreted with caution. Finally, whilst this study focussed on protein-altering single nucleotide variants and small indels, emerging evidence highlights the role of non-coding and large structural variants in rare disease pathogenesis.^29^^,^^30^ Future studies should assess how genetic ancestry influences the prioritisation of all variant types captured by genome sequencing.

In conclusion, the effectiveness of variant prioritisation approaches using genome sequencing data from patients with rare disease remains limited by the lack of diversity in population reference datasets, and by the broad ancestry groupings used to stratify allele frequency estimates. This can lead to an increased number of false positives amongst prioritised variants, potentially resulting in a higher burden of VUS. This has consequences for clinical efficiency and patient care, since false positives can increase clinical scientist workload and associated costs, while unresolved VUS can delay decision-making and contribute to uncertainty around treatment and management.^31^ Increased representation of diverse genetic ancestries in genomic reference databases and use of ancestry-appropriate allele frequency estimation is essential for achieving equity in variant prioritisation for rare disease diagnosis.

## Contributors

S.T., K.K, L.M and M.B designed the study. S.T. carried out the analysis. L.M., K.K., M.J.S and M.B supervised the work. S.T. and L.M verified the underlying data. S.T., M.J.S., and K.K. wrote the first draft of the manuscript. M.M, T.N, Y.C. J.E, and D. K contributed to discussion of the results and reviewed and edited the manuscript. All authors read and approved the final version of the manuscript.

## Data sharing statement

The data supporting the findings of this study are available within the Genomics England Research Environment. To access genomic and clinical data within the Research Environment, researchers must first apply to become a member of either the Genomics England Research Network (academics/healthcare professionals) or the Discovery Forum (industry) via the Genomics England website (https://www.genomicsengland.co.uk/research). Analysis code is available online at https://gitlab.com/genomicsengland/Data_Diversity_Public/rare-diseases-ancestry-paper.

## Declaration of interests

All authors have completed the ICMJE uniform disclosure form and declare no support from any organisation for this work, other than the funding information provided. There are no financial relationships or other conflicts that could have influenced this manuscript and no competing interests exist.
