# Supplementary material for: Equity in genome sequencing for rare disease diagnosis: a cross-sectional analysis of data from the UK 100,000 Genomes Project
Source: eBioMedicine. 2026 Aug 26;131:106452. doi: 10.1016/j.ebiom.2026.106452 (PMC13543812; doi:10.1016/j.ebiom.2026.106452)
Supplement: Supplementary file 1 [file mmc1.pdf]

Supplementary Materials

Equity in genome sequencing for rare disease diagnosis: a cross-sectional analysis of data from the UK 100,000 Genomes Project

Sam Tallman[1]*, Loukas Moutsianas[1], Thuy Nguyen[1], Yoonsu Cho[1,2], Maxine Mackintosh[1,3], Dalia Kasperaviciute[1], Matthew A Brown[1,4], Jamie M. Ellingford[1,5,6], Karoline Kuchenbaecker[1,7]* and Matt J Silver[1,8,9]*

1. Genomics England, London, UK
2. Medical Research Council Integrative Epidemiology Unit, University of Bristol, Bristol, UK
3. Alan Turing Institute, London, UK
4. Department of Medical and Molecular Genetics, King's College London, London, UK
5. Division of Evolution, Infection and Genomic Sciences, School of Biological Sciences, Faculty of Biology, Medicine and Health, University of Manchester, Manchester, UK
6. Manchester Centre for Genomic Medicine, St Mary's Hospital, Manchester University NHS Foundation Trust, Health Innovation Manchester, Manchester, UK
7. Division of Psychiatry, University College London, UK
8. Department of Non-communicable Disease Epidemiology, London School of Hygiene & Tropical Medicine, UK
9. Medical Research Council Unit The Gambia at the London School of Hygiene and Tropical Medicine, Serrekunda, The Gambia

* Corresponding authors

Supplementary Materials

Contents

Supplementary Appendix

Genomics England's Small Variant Prioritisation Pipeline 5

Routes to identifying diagnostic SNVs and indels in the 100,000 Genomes Project 5

Genetic ancestry group inference 7

Construction of an ancestry-group stratified allele frequency reference panel and application of filtering allele frequency thresholds 8

Stratified analyses by variant consequence and inheritance patterns 11

Sensitivity analyses using updated virtual gene panel versions 11

Genetic heterogeneity within ancestry groups 12

Balanced down-sampling of ancestry groups 13

Ancestry group differences in frequency of recorded variants of uncertain significance (VUS) 13

Supplementary Tables

Supplementary Table 1 – Distribution of standardised disease groups across the cohort by genetically inferred ancestry groups 15

Supplementary Table 2 - Population allele frequency filters used by Genomics England's variant prioritisation pipeline (version 5) during the 100kGP 16

Supplementary Table 3 - Numbers of probands assigned to each genetically inferred ancestry group, the reference populations merged to create them, the place of birth of individuals that comprised that reference group (taken from Prive et al. 2022), and comparisons with 16+1 and other reported parental ethnicities. 17

Supplementary Table 4 - Within-group KING kinship coefficient summary statistics per ancestry group within the UK COVID-19 Genomics Study dataset following the exclusion of individuals with a kinship coefficient ≤ 0.0442 (removal of 3rd degree relatives and below). 19

Supplementary Table 5 - Population sizes and 95% filtering allele frequency (FAF) thresholds derived from the UK COVID-19 Genomics Study and retrospectively integrated into the automated variant prioritisation pipeline. 20

Supplementary Table 6 - Population sizes and 95% filtering allele frequency (FAF) thresholds derived from the external gnomADv4.1 reference database and retrospectively integrated into the automated variant prioritisation pipeline. 21
Supplementary Table 7 - Summary statistics of variant counts returned by the automated variant prioritisation pipeline by genetically inferred ancestry group. 22
Supplementary Table 8 - Negative binomial mixed effects regression model output (prioritised variants as counts variable). 23
Supplementary Table 9 - Summary statistics of outcomes recorded in the clinical scientist outcomes questionnaires (diagnostic variants, VUS) for 100kGP probands stratified by genetically inferred ancestry groups. 24
Supplementary Table 10 - Logistic mixed effects regression model output (diagnostic variant recorded = yes or no, as binary variable). 25
Supplementary Table 11 - Logistic mixed effects regression model output (variant level sensitivity; prioritised diagnostic variants / total diagnostic variants). 26
Supplementary Table 12 - Logistic mixed effects regression model output (variant level PPV; prioritised diagnostic variants / total prioritised variants). 27
Supplementary Table 13 - Logistic mixed effects regression model output (recorded VUS carrier status = yes or no, as binary variable). 28
Supplementary Table 14 - Number and proportion of variants in the 100,000 Genomes Project cohort, stratified by genetic ancestry group, variant set, and allele frequency filtering source. 29
Supplementary Table 15 - Classified VUS recorded in the outcomes questionnaire in that were initially prioritised and returned to clinical scientists during the 100kGP return of primary rare disease findings; yet were filtered out using the ancestry group stratified FAF filters from the UK COVID-19 Genomics Study. 31
Supplementary Table 16 - Number and proportion of diagnostic SNVs and indels in the 100,000 Genomes Project cohort, stratified by ancestry group and pipeline return status. 37
Supplementary Table 17 - Number and proportion of diagnostic SNVs and indels in the 100,000 Genomes Project cohort, stratified by genetic ancestry group, pipeline return status, and Exomiser ranking. 38

Supplementary Figures

Supplementary Figure 1 - Ethnicity distribution of 100kGP probands. 39
Supplementary Figure 2 - Projection of 100kGP probands onto UK Biobank PCs (2-8) 40
Supplementary Figure 3 - Projection of 100kGP probands onto the UK Biobank PCs (11-14) 41
Supplementary Figure 4 - Approximate genetic distance between 100KGP probands and UK Biobank reference groups. 42

Supplementary Materials

Supplementary Figure 5 - Pairwise overlap between UK Biobank reference groups among 100kGP probands assigned to one or more groups at an approximate F_{ST} threshold of < 0.005, before consolidation into broader ancestry groups 43
Supplementary Figure 6 – Graph-based consolidation of 100kGP probands into broader ancestry groups 44
Supplementary Figure 7 - Histograms showing the numbers of variants returned by the automated pipeline per probands per variant type...45
Supplementary Figure 8 - Heatmaps showing the mean number of variants returned by the automated pipeline per proband by ancestry group, inheritance pattern and molecular consequence. 46
Supplementary Figure 9 - Heatmaps showing the relative change in the mean number of variants returned by the automated pipeline per proband, compared with the European ancestry group, stratified ancestry group, inheritance pattern, and molecular consequence...47
Supplementary Figure 10 – Distribution of prioritised diagnostic variants per ancestry group by inheritance patterna and variant consequence categories 48
Supplementary Figure 11 – Differences in recorded variant of unknown significance (VUS) frequency, stratified by genetically inferred ancestry group. 49
Supplementary Figure 12 - Projection of UK COVID-19 Genomics Study participants onto UK Biobank PCs (2-8) 50
Supplementary Figure 13 - Projection of UK COVID-19 Genomics Study participants onto UK Biobank PCs (11-14) 51
Supplementary Figure 14 - Approximate genetic distance between UK COVID-19 Genomics Study participants and UK Biobank reference groups 52
Supplementary Figure 15 - Pairwise overlap between UK Biobank reference groups among UK COVID-19 Genomics Study participants assigned to one or more groups at an approximate F_{ST} threshold <0.005, before consolidation into broader ancestry groups. 53
Supplementary Figure 16 - Graph-based consolidation of UK COVID-19 Genomic Study participants into broader ancestry groups..54
Supplementary Figure 17 – Filtering allele frequency (FAF) distributions and statistics from the application of UK COVID-19 Genomic Study reference panel to prioritised variants in the 100kGP.55
Supplementary Figure 18 - Filtering allele frequency (FAF) distributions and statistics from the application of UK COVID-19 Genomic Study reference panel to candidate variants (outside virtual gene panels) in the 100kGP 56
Supplementary Figure 19 - Impact of additional population frequency filters on candidate variants outside virtual panels across ancestry groups. 57
Supplementary Figure 20 - Sensitivity and positive predictive value of prioritised variants (using updated virtual gene panels), stratified by genetically inferred ancestry group. 58

Supplementary Materials

Supplementary Figure 21 - Convex ancestry proportions of 100kGP rare disease probands estimated using UK Biobank reference groups, stratified by assigned genetic ancestry group. 59
Supplementary Figure 22 - Distribution of within-group genetic heterogeneity across ancestry groups in the 100kGP rare disease cohort 60
Supplementary Figure 23 - Adjusted incidence rate ratios (IRRs) and odds ratios (ORs) comparing genetically inferred ancestry groups with the European group under the full cohort and after exclusion of the most peripheral 20% of individuals within each ancestry group 61
Supplementary Figure 24 - Adjusted incidence rate ratios (IRRs) and odds ratios (ORs) comparing genetically inferred ancestry groups with the European reference group in the full cohort and after balanced down sampling to 124 probands per ancestry group across 500 iterations. 62

Supplementary Materials

Genomics England's Small Variant Prioritisation Pipeline

To aid NHS clinical scientist evaluation of primary findings for rare diseases in the 100,000 Genomes Project (100kGP)[1], single nucleotide variants (SNVs) and indels in families are filtered and prioritised using an automated, virtual panel-based variant prioritisation pipeline, formally referred to as the Rare Disease Small Variant Tiering Process in Genomics England's documentation. In this study, we analysed version 5.0 of the pipeline, specifically as applied to participants in the 100kGP rare disease programme whose genomes were aligned to the GRCh38 reference build.

Complete documentation is available online: https://re-docs.genomicsengland.co.uk/gel_rare_disease.pdf

Note, in Genomics England's documentation, returned variants are classified as:

- **Tier 1**: High-confidence de novo or predicted loss-of-function (LoF) variants within genes on the applied virtual gene panels, consistent with the specified inheritance model and passing filtering criteria.
- **Tier 2**: Other protein-altering or splice-region variants (e.g. missense, in-frame indels) in panel genes that pass filtering but do not meet Tier 1 criteria.
- **Tier 3**: Protein-altering variants outside the applied panels, yet also pass segregation pattern and frequency filters

In this study, we did not distinguish between Tier 1 and Tier 2 variants, referring to all variants returned within the applied virtual gene panel(s) by the automated pipeline as *prioritised variants* (see Figure 1 in main text). Tier 3 variants (i.e. outside panel) were analysed separately. Note, where a variant was returned multiple times for a given proband (e.g. under different applied virtual gene panels or plausible inheritance models), it was counted a single time.

Supplementary Table 7 summarises the total number of prioritised variants, including the number of unique variants, variant burden per proband, and the proportion of probands with >1 variant, stratified by genetically inferred ancestry group. Variant count distributions per proband across the analysis cohort are visualised in Supplementary Figure 7.

A Table summarising the population frequency filters used by the automated variant prioritisation pipeline (v5; GRCh38) is shown in Supplementary Table 2.

Routes to identifying diagnostic SNVs and indels in the 100,000 Genomes Project

Diagnostic SNVs and indels analysed in this study were those ultimately recorded as pathogenic or likely pathogenic variants involved in solving or partially solving a case in linked outcome questionnaires after the manual review by clinical scientists. The primary automated route by which clinical scientists recovered such variants in the 100kGP was through accessing the results returned through the Genomics England virtual panel-based prioritisation pipeline (see above and Methods; main text). However, because this approach is designed to reduce the number of variants requiring manual review, some diagnostic variants may not be returned within the

prioritised set —for example, because they fall outside the applied virtual gene panels, do not meet the pipeline's default thresholds for predicted consequence (e.g. non-coding), allele frequency, segregation pattern, or are not PASS within the FILTER field in one or more of the family VCFs. To reduce the possibility of missed or inefficiently prioritised diagnoses resulting from these filtering steps, additional complementary routes for manual variant review were also available.

First, all rare disease cases in the 100kGP were also processed through Exomiser[2], which prioritises rare coding SNVs and indels using pathogenicity, inheritance, and phenotype information (using recorded Human Phenotype Ontology [HPO] terms). Its ontology-driven phenotype matching can help identify diagnoses in patients with atypical clinical presentations. Exomiser returned ranked variants, where higher-ranked variants represent those most likely to explain the proband's phenotype given the applied criteria. These rankings were displayed in the Interpretation Browser alongside variants returned by the automated panel-based prioritisation pipeline. Exomiser rankings could therefore apply both to variants also returned by the panel-based pipeline and to additional variants outside that set.

Beyond these outputs, clinical scientists could use the Interpretation Browser to review read-level support, inspect candidate variants returned outside the applied virtual gene panels, apply custom filters to search for variants beyond those returned by the pipeline, and download family-level VCF files for extended manual inspection. This enabled recovery of diagnostic SNVs and indels not captured within the prioritised set, including variants in genes newly associated with disease after the original panel selection or variants identified through broader case-level review of family VCF data.

The relative contribution of these different routes is summarised in Supplementary Table 16. Overall, 80·9% of diagnostic SNVs and indels were prioritised variants returned by Genomics England's virtual panel-based pipeline, 11·4% were those returned by the pipeline as candidate variants outside the applied virtual gene panels, and 7·7% were not returned by the automated prioritisation pipeline. Supplementary Table 17 further shows the extent to which diagnostic variants from each of these categories were also ranked within the top five Exomiser results returned during interpretation.

Self-reported ethnicity

Self-reported ethnicity labels were collected for all probands and family members in the 100kGP at recruitment, using the standard 16+1 categories defined in the NHS data dictionaries (https://www.datadictionary.nhs.uk). Among the analysis cohort, these labels were available for 14.6% (4292/29405), with the remainder recorded as 'Not known' or not stated. Additionally, free-text columns for "other" parental ethnicities were available for a subset of participants (2612/29,405; 8.9%).

We assessed the representativeness of self-reported ethnic groups in the cohort relative to the general population in England, by comparing with the 2021 Office for National Statistics (ONS) data (https://www.ons.gov.uk/peoplepopulationandcommunity/culturalidentity/ethnicity/datasets/populationestimatesbyethnicgroupenglandandwales).

Supplementary Materials

To harmonise categories, ONS 2021 ethnicity labels were updated to match 16+1 nomenclature (e.g., “Asian, Asian British, or Asian Welsh” → “Asian or Asian British”). ONS categories not directly mapped to the 16+1 set—specifically “White: Gypsy or Irish Traveller,” “White: Roma,” and “Other ethnic group: Arab”—were merged into “White: Any other White background,” “White: Any other White background,” and “Other ethnic groups: Any other ethnic group,” respectively. These mappings were chosen to align with the 16+1 system, but comparisons between ONS data should be considered approximate.

To account for the strong age–ethnicity structure in England, we stratified both datasets by decade (from ages 0 to 70+) and computed the proportion of individuals within each ethnic group per age stratum. Individuals in the 100kGP cohort with unknown or unstated ethnicity were excluded. Broad agreement was observed across age-stratified trends between the study cohort and ONS data (Supplementary Figure 1). A notable exception was over-representation of “Asian or Asian British: Pakistani” individuals in deciles under age 30, consistent with elevated consanguinity rates and associated recessive disease burden reported among British Pakistanis. Other modest deviations may reflect demographic or recruitment biases and warrant further investigation beyond the scope of this analysis.

Genetic ancestry group inference

We assigned all 29 405 rare disease probands in our 100kGP analysis cohort (see Methods; main text) to one of eight ancestry groups using externally defined, data-driven reference groups previously curated using data from the UK Biobank[3] described in Privé *et al* 2022[4]. This panel was selected because it captures genetic structure present within the UK population at scale and is independent of the commonly used reference databases that were also used for frequency filtering within the automated small variant prioritisation pipeline (gnomADv2[5], 1000 Genomes Project[6]).

The 63,523 sites used for genetic ancestry inference were common, high-quality, linkage disequilibrium (LD) pruned single nucleotide variants (SNVs) previously curated by the Genomics England bioinformatics team. Sites were selected based on the following criteria: (1) biallelic SNVs; (2) minor allele frequency >5% in both the 1000 Genomes Phase III[6] and the Genomics England multi-sample variant aggregate (aggV2; https://re-docs.genomicsengland.co.uk/aggv2/) (3) median genotype quality >30; (4) allele balance ratio >0.9; (5) genotype call rate >90%; (6) exclusion of SNVs within regions of long-range LD; (7) exclusion of ambiguous A/T or G/C SNPs; (8) LD pruning using PLINK[7] v1.9 with an r^2 threshold of 0.1 in 500 kb windows; and (9) Hardy-Weinberg Equilibrium p-value >1e^{-5}. After applying these filters, 63,523 SNPs were retained. Of these, 55,706 overlapped with the UK Biobank reference dataset from Privé *et al* 2022, which included ~4 million sites, and were used for principal component (PC) projection.

The *bigsnpr*[8] R package was first used to project proband genotypes and allele frequencies from an initial set of 21 reference groups across these 55,706 intersecting sites onto the top 16 linkage LD-scaled PCs created from the reference panel (Supplementary Figures 2 and 3). Squared Euclidean distances on the PC space (normalised to approximate F_{ST}) were then calculated between each proband and these 21 reference groups (Supplementary Figure 4), and probands were assigned to any group if F_{ST} <0.005, following the threshold used in the *bigsnpr*

documentation (https://privefl.github.io/bigsnpr/articles/ancestry.html). This allowed individuals to be assigned to multiple reference groups where genetic similarity overlapped (89.3% of individuals assigned to multiple reference groups at $F_{ST} < 0.005$).

As mentioned in Privé *et al* 2022[4], this reference dataset contains relatively granular reference groups, particularly within Europe, reflecting its sampling composition. To avoid over-fragmentation and simplify reporting, we therefore applied a secondary consolidation step to merge genetically similar reference groups. Using a custom R script, an edge was drawn between two groups when at least 50% of individuals in the 100kGP rare diseases cohort assigned to one group were also assigned to the other at $F_{ST} < 0.005$ (directional "containment"; Supplementary Figure 5B), and final clusters were defined as connected components in the resulting graph, allowing transitive merging of overlapping groups. Because directional overlap criteria can lead to absorption of smaller but distinguishable groups into larger neighbouring groups, we also examined symmetric overlap using the Jaccard index (intersection divided by union; Supplementary Figure 5A). This was particularly relevant for the Middle Eastern reference group, for which approximately 72% of assigned individuals were also assigned to the Italy reference group at approximate $F_{ST} <0.005$, despite low symmetric overlap (maximum Jaccard overlap with Italy 4%). We therefore retained the Middle Eastern ancestry group as distinct in the consolidated classification. For the remaining reference groups, Jaccard thresholds of >8% to >36% supported the same broad merged structure, with the minor exception of Finland (n=6), which would have remained distinct under a strict symmetric overlap criterion but was retained within the broader European group because of its very small size. This process yielded 11 merged groups (Supplementary Figure 6).

From this set of merged groups, those with fewer than 100 probands were combined into a single "remaining participants" category to avoid reporting imprecise group-level estimates for very small clusters. In practice, this applied to the South America (n=27), Philippines (n=56), and Japan (n=8) groups. This category also included probands who could not be assigned to any of the 21 reference groups using the above criteria, likely reflecting recent admixture or divergence from the available reference groups due to under-representation in the reference dataset.

This resulted in a final set of eight ancestry groups, plus a remaining participant group. Further details are provided in Supplementary Table 3, including the reference groups that were merged, the place of birth of individuals in the reference groups, as well as comparisons with 16+1 ethnicity available additional self-reported parental ethnicities where available.

Construction of an ancestry-group stratified allele frequency reference panel and application of filtering allele frequency thresholds

We constructed an allele frequency reference panel using a multi-sample aggregate of SNVs and small indels from 34,701 whole-genome sequenced participants without rare diseases, curated as part of a UK COVID-19 Genomics study as described in Kousathanas et al. 2021[9].

Supplementary Materials

To derive a set of genetically unrelated individuals, we applied a kinship coefficient threshold of ≥0.0442 (exclusion of all 3rd degree relatives and below), using estimates previously generated by the Genomics England bioinformatics team via the PLINK2 implementation of the KING-robust algorithm[10]. More distant relatedness is therefore not systematically removed, owing to the challenges of inferring kinship coefficients >3rd degree. This yielded a set of 33,724 individuals.

These individuals were then assigned to eight genetic ancestry groups (plus a remaining participants group), following the ancestry inference strategy described for the rare disease cohort (Supplementary Appendix p7). Applying the same assignment threshold and merging framework yielded the same 11 merged groups in the UK COVID-19 Genomics Study cohort (Supplementary Figures 12-16). For consistency with the 100kGP cohort, Philippines, Japan, South America, and unassigned groups were collapsed into a single "remaining participants" group. However, we note the Philippines group was more highly represented in the COVID-19 Genomics Study (n=390) than in the 100kGP cohort (n=59), likely reflecting differences in recruitment and sampling. Final group sizes in the reference panel as shown in Supplementary Tables 4 and 5.

We additionally summarised within-group pairwise KING-robust kinship coefficients across ancestry groups by extracting all pairwise comparisons in which both participants belonged to the same group, and calculating the mean, maximum, and number of within-group comparisons. Mean within-group kinship coefficients were low across all ancestry groups (Supplementary Table 4). However, because KING kinship estimates are intended for inference of close relatedness and are not reliable for interpretation at lower levels of relatedness, these results were interpreted descriptively only.

To generate ancestry-stratified allele frequency estimates, we used aggregated SNV and indel variant calls from Genomics England's release v5 of the UK COVID-19 Genomics Study, spanning the autosomes and X chromosome. Multi-allelic variants were decomposed into biallelic form. Genotypes were masked if they failed the following thresholds:

- Autosomal + female X genotypes: depth (DP) < 10, genotype quality (GQ) < 20, or allele balance (AB) binomial test $p < 10^{-3}$
- Male X (non-PAR)**:** DP < 5 or GQ < 20

In total, the aggregated dataset included 505,993,047 SNVs and small indels across 33,724 participants. For all SNVs and indels, we calculated allele counts (AC) and allele numbers (AN) stratified by ancestry group using PLINK2, separately for the autosomes and X chromosome. In non-pseudo autosomal regions of the X, male genotypes were counted as haploid. Only variants PASS variants with greater than >90% coverage across the dataset were included.

Using this dataset, we calculated conservative, ancestry group-specific estimates of allele frequency using the filtering allele frequency (FAF) approach described by Whiffin et al 2017[11]. We defined FAF as the lower bound of the 95% confidence interval for the true population allele frequency, given the observed allele count and sample size**,** using a Poisson model, with singletons (AC = 1) conservatively assigned a FAF of 0. This provides a cautious

estimate of how common a variant could plausibly be, helping to avoid misclassifying variants as too common to be disease-causing when they may only appear at a high frequency due to limited sampling size within a given ancestry group.

We evaluated the potential of this diverse, ancestry-matched reference panel by retrospectively integrating the derived FAF thresholds into the automated variant prioritisation pipeline used in the 100kGP (Supplementary Appendix p5). Specifically, across all 5,719,061 variants (1,974,633 unique; whether within the applied virtual panels or not) that passed the standard filtering criteria during 100kGP—including population frequency thresholds from gnomAD v2, 1000 Genomes, and custom controls (Supplementary Table 2) we additionally applied FAF thresholds of 0.01 for variants flagged under recessive inheritance models and 0.001 for those flagged under dominant models (see Supplementary Table 14). To maintain consistency with the variant prioritisation pipeline versions currently used by the NHS Genomic Medicine Service (GMS) in the UK (https://genomicsengland.gitlab.io/rare_disease_analysis/rare-disease-analysis-guide/Mira/), we retained all variants classified as "Pathogenic" or "Likely Pathogenic" in ClinVar[12] (as of March 2024), regardless of their observed frequencies. 100kGP cohort-wide FAF distributions of variants originally returned by the pipeline, and the number of such variants that failed these thresholds, are shown in Supplementary Figures 17 and 18, stratified by reference group and whether the variant was prioritised based on its appearance within the applied virtual gene panel.

Of note, 46 previously prioritised variants that were recorded as variants of uncertain clinical significance (VUS) in clinical scientist-issued outcomes questionnaires were filtered out using these ancestry group-specific FAF thresholds. Full details of these variants, including their maximum observed frequencies across ancestry groups, are provided in Supplementary Table 15.

For comparison, we also derived FAF thresholds from the combined gnomAD[13] v4.1 dataset comprising 730 947 exomes and 76 215 genomes into seven populations (the Finnish and Amish groups in the gnomAD v4.1 release were excluded from this analysis). Variants failing quality control (QC) filters, showing highly discrepant frequencies between exomes and genomes, or with callable coverage in less than 50% of individuals in the dataset were excluded to minimise the inclusion of technical artefacts and improve the reliability of allele frequency estimates. Using the same Poisson-based approach applied to the UK COVID-19 Genomics Study panel, we calculated population-specific FAFs across SNVs and indels in gnomAD v4.1 spanning the autosomes and X chromosome. These FAF-based thresholds - 0.001 for dominant and 0.01 for recessive inheritance models (Supplementary Table 6) - were then applied in addition to the standard filtering criteria used by the automated variant prioritisation pipeline applied to each family.

Figure 5 (main text) illustrates the impact of applying these updated frequency thresholds - derived from either the UK COVID-19 Genomics Study reference panel or gnomAD v4.1 - on the number of variants prioritised relative to the standard pipeline, stratified by the assigned ancestry group of the probands these variants were identified in. An equivalent plot for candidate variants outside the applied virtual gene panels is provided in Supplementary Figure 19.

Supplementary Materials

Stratified analyses by variant consequence and inheritance patterns

We assessed whether the number and characteristics of variants prioritised during the 100kGP rare disease programme - both those returned by the automated pipeline and those subsequently confirmed as diagnostic - varied across genetically inferred ancestry groups when stratified by molecular consequence and inferred inheritance pattern.

Variant consequences were grouped into the following categories based on Sequence Ontology terms recorded by the pipeline:

- Protein-truncating variants: stop-gained, frameshift, stop-lost, initiator codon, and start-lost (SO:0001587, SO:0001589, SO:0001578, SO:0001582, SO:0002012).
- Canonical splice-site variants: splice donor or acceptor sites (SO:0001574, SO:0001575)
- Missense variants: altered the amino acid sequence without introducing a stop (SO:0001583)
- In-frame indels: insertions or deletions that did not disrupt the reading frame (SO:0001821, SO:0001822)
- Splice-region variants: near but not within canonical splice sites (SO:0001630)
- Varies by transcript: reflecting transcript-specific differences in predicted consequence

Inheritance patterns were inferred from segregation tags generated by the pipeline and grouped as:

- Dominant *(including inherited autosomal dominant and imprinted)*
- Recessive (homozygous)
- Recessive (compound heterozygous)
- De novo
- X-linked
- Mitochondrial
- Multiple plausible *(more than one plausible model supported)*

These groupings were used to stratify two outcomes across genetically inferred ancestry groups: (1) the average number of prioritised variants per proband, and (2) the distribution of confirmed diagnostic variants (among those prioritised) across consequence types and inheritance patterns. Results are shown in Supplementary Figures 8/9 and Supplementary Figure 10, respectively.

Sensitivity analyses using updated virtual gene panel versions

During the 100kGP analysis of primary clinical findings for rare diseases, virtual gene panels used to prioritise variants for manual review were selected by clinicians based on pre-test phenotypic information and applied using the then-current versions of Genomics England's PanelApp resource (https://panelapp.genomicsengland.co.uk/), a dynamic, crowd-sourced platform that is continuously updated with evidence on gene–disease associations – including throughout the initial application of the automated variant prioritisation pipeline following sequencing.

Supplementary Materials

To assess the robustness of ancestry-associated differences in pipeline performance - specifically the sensitivity and positive predictive value (PPV) of prioritised variants reported in the main text - we repeated key regression analyses using an updated set of virtual gene panels.

For this, we re-annotated variants previously returned outside of virtual gene panels using a set of updated PanelApp panel versions from March 1st, 2024 (https://gitlab.com/genomicsengland/Data_Diversity_Public/rare-diseases-ancestry-paper). We restricted to high-confidence ('Green') genes and matched panel names and inheritance models to those originally applied for each proband. This process identified 29 075 additional variants located within relevant disease genes, increasing the total number of prioritised variants to 85 717 across the cohort.

All multivariable regression models evaluating ancestry group differences in PPV and sensitivity (as described in the *Statistical Analysis* section of the main text) were re-run using this updated set of prioritised variants. Results are shown Supplementary Figure 20.

Genetic heterogeneity within ancestry groups

Human genetic ancestry is continuous, and any assignment of individuals to discrete ancestry groups is an analytical simplification[14,15]. Because our primary analyses used ancestry groups as categorical exposures in regression models, we undertook two supplementary analyses to characterise within-group heterogeneity and the extent of mixed inferred genetic ancestries among participants.

First, we estimated convex genetic ancestry proportions (α) for each 100kGP rare disease participant relative to the UK Biobank reference groups using the quadratic programming approach described by Privé et al. 2022[4]. Coefficients were constrained to be non-negative and to sum to 1 across reference groups and were then aggregated across the consolidated ancestry groups used in the main analyses (Supplementary Table 3). The resulting profiles are shown in Supplementary Figure 21. As expected, participants assigned to each of the eight ancestry groups generally showed dominant contributions from their assigned group, but with varying degrees of secondary contributions from other groups. Heterogeneity was most evident among participants labelled as remaining participants, whose ancestry proportion profiles showed substantial contributions from multiple inferred ancestries.

Second, we quantified within-group dispersion in the projected principal component (PC) space used for ancestry assignment by calculating the Euclidean distance from each 100kGP rare disease participant to the PC centroid of their assigned ancestry group (Supplementary Figure 22). Within-group heterogeneity was greatest among the remaining participants group, with more modest but variable levels of dispersion across the other ancestry groups. To evaluate whether the main findings were sensitive to this heterogeneity, we repeated the primary multivariable analyses (see Methods: main text) after excluding the most peripheral 20% of individuals within each group, defined as those in the highest quintile of distance from the group-specific PC centroid. As shown in Supplementary Figure 23, effect estimates for the number of prioritised variants, diagnostic yield, sensitivity of prioritisation, and the proportion of prioritised variants recorded as diagnostic were concordant with results using

the full cohort. These findings indicate that the principal associations were not driven by peripheral individuals within more heterogeneous groups.

Nonetheless, these analyses also reinforce that discrete ancestry groupings cannot fully capture the continuum of genetic diversity, and that future approaches based on local ancestry or continuous genetic similarity may improve interpretation.

Balanced down-sampling of ancestry groups

To evaluate the potential impact of unequal ancestry group sample sizes on the primary results, we performed balanced down sampling analyses for the main multivariable models. In each iteration, participants were randomly subsampled within each ancestry group to the size of the smallest included group (n=124 per group), the fully adjusted model was refitted, and this procedure was repeated 500 times. For the sensitivity of prioritisation outcome, defined as the proportion of recorded diagnostic variants that were prioritised, an additional minimum-count restriction was applied. Because this measure is conditional on recorded diagnostic variants, balanced down sampling can produce very small denominators, or denominators of zero, in some ancestry groups, leading to model-fitting failure. We therefore fitted this model only in iterations in which each ancestry group contained at least 10 recorded diagnostic variants. For each ancestry group coefficient, we summarised the mean and empirical 95% interval of the exponentiated effect estimates across iterations and compared these with the corresponding estimates from the full-cohort model.

Balanced down sampling produced effect estimates that were broadly concordant with those from the full-cohort analyses (Supplementary Figure 24). In particular, the significant ancestry group effects remained consistent in direction and magnitude, although confidence intervals widened as expected under reduced sample size. This widening was most evident for more modest differences between larger groups (e.g. European vs South Asian) and is consistent with reduced precision and statistical power rather than material change in the underlying effect estimates. Overall, these analyses suggest that the principal ancestry group associations were not driven by sample-size imbalance.

Ancestry group differences in frequency of recorded variants of uncertain significance (VUS)

Unlike diagnostic SNVs and indels, which are systematically recorded by clinical scientists in the outcomes questionnaire, variants of uncertain significance (VUS) are not captured systematically. Instead, VUS may be entered optionally by clinical scientists if identified and documented during manual review. Accordingly, this analysis concerns the frequency of *recorded* VUS rather than the complete underlying burden of VUS identified in each proband.

Recorded VUS were analysed as a binary outcome indicating whether a proband had at least one recorded VUS in the outcomes questionnaire. We used this approach rather than modelling the total number of VUS per proband because the empirical distribution of recorded VUS was sparse and zero-inflated, with 9.8% of probands having at least one recorded VUS only 2.6% of probands having more than one VUS recorded (Supplementary Table 7).

Supplementary Materials

A binary outcome therefore provided a more robust and interpretable measure, reducing sensitivity to unstable variation in low counts.

We fitted mixed-effects logistic regression models analogous to the main analyses, first unadjusted and then adjusted for age at consent, sex, family structure, family disease affection, and predicted consanguinity, with random intercepts for disease group and handling GMC. Compare to the European ancestry group, the adjusted model showed higher odds of having at least one recorded VUS in the East African (OR 1.90, 95% CI 1.17–3.10; p=0.0097), South Asian (1.35, 1.15–1.59; p=0.0003), West African (1.36, 1.05–1.77; p=0.0217), and remaining participant groups (1.45, 1.17–1.78; p=0.0005). Full effect estimates are provided in Supplementary Table 13 and group differences plotted in Supplementary Figure 11.

Supplementary Materials

Supplementary Table 1 – Distribution of standardised disease groups across the cohort by genetically inferred ancestry groups

	European	East Asian	Middle Eastern	Ashkenazi	South Asian	North African	East African	West African	Remaining participants
N total	23623	124	452	250	3030	147	159	679	941
**Disease group**									
Neurology and neurodevelopmental disorders	9729 (41.2%)	44 (35.5%)	162 (35.8%)	99 (39.6%)	1220 (40.3%)	62 (42.2%)	72 (45.3%)	219 (32.3%)	396 (42.1%)
Cardiovascular disorders	2835 (12.0%)	7 (5.6%)	29 (6.4%)	25 (10.0%)	214 (7.1%)	9 (6.1%)	<5	84 (12.4%)	74 (7.9%)
Renal and urinary tract disorders	2253 (9.5%)	24 (19.4%)	80 (17.7%)	39 (15.6%)	308 (10.2%)	24 (16.3%)	19 (11.9%)	123 (18.1%)	124 (13.2%)
Ophthalmological disorders	1778 (7.5%)	18 (14.5%)	61 (13.5%)	19 (7.6%)	397 (13.1%)	18 (12.2%)	26 (16.4%)	111 (16.3%)	70 (7.4%)
Ultra-rare disorders	1280 (5.4%)	7 (5.6%)	20 (4.4%)	8 (3.2%)	187 (6.2%)	10 (6.8%)	7 (4.4%)	17 (2.5%)	42 (4.5%)
Tumour syndromes	1273 (5.4%)	<5	9 (2.0%)	12 (4.8%)	47 (1.6%)	<5	<5	19 (2.8%)	22 (2.3%)
Skeletal disorders	599 (2.5%)	-	12 (2.7%)	8 (3.2%)	66 (2.2%)	<5	<5	10 (1.5%)	23 (2.4%)
Endocrine disorders	542 (2.3%)	5 (4.0%)	6 (1.3%)	8 (3.2%)	86 (2.8%)	<5	<5	11 (1.6%)	38 (4.0%)
Hearing and ear disorders	460 (1.9%)	<5	18 (4.0%)	5 (2.0%)	103 (3.4%)	<5	<5	11 (1.6%)	35 (3.7%)
Haematological and immunological disorders	510 (2.2%)	<5	9 (2.0%)	5 (2.0%)	44 (1.5%)	5 (3.4%)	<5	12 (1.8%)	10 (1.1%)
Metabolic disorders	400 (1.7%)	<5	12 (2.7%)	<5	103 (3.4%)	<5	<5	13 (1.9%)	24 (2.6%)
Dysmorphic and congenital abnormality syndromes	407 (1.7%)	<5	10 (2.2%)	<5	45 (1.5%)	<5	<5	10 (1.5%)	19 (2.0%)
Multiple disorders	388 (1.6%)	<5	6 (1.3%)	<5	54 (1.8%)	-	5 (3.1%)	10 (1.5%)	14 (1.5%)
Dermatological disorders	208 (0.9%)	-	<5	<5	67 (2.2%)	<5	<5	16 (2.4%)	19 (2.0%)
Respiratory disorders	267 (1.1%)	-	<5	6 (2.4%)	12 (0.4%)	-	<5	<5	5 (0.5%)
Ciliopathies	213 (0.9%)	<5	9 (2.0%)	<5	41 (1.4%)	<5	<5	<5	< 5
Rheumatological disorders	211 (0.9%)	<5	<5	<5	12 (0.4%)	<5	<5	<5	6 (0.6%)
Growth disorders	137 (0.6%)	-	-	-	10 (0.3%)	<5	<5	<5	6 (0.6%)
Gastroenterological disorders	85 (0.4%)	-	<5	<5	13 (0.4%)	<5	-	<5	7 (0.7%)
Other disorders	48 (0.2%)	<5	-	-	<5	<5	<5	<5	< 5

Supplementary Materials

Supplementary Table 2 - Population allele frequency filters used by Genomics England's variant prioritisation pipeline (version 5) during the 100kGP

Database	Sequencing	Population	#	AF threshold Recessive	AF threshold Dominant	AF threshold Mitochondrial
gnomAD v2.1	Exomes	African / African American (AFR)	8,128	0.01	0.001	_
gnomAD v2.1	Exomes	Admixed American (AMR)	17,296	0.01	0.001	_
gnomAD v2.1	Exomes	East Asian (EAS)	9,197	0.01	0.001	_
gnomAD v2.1	Exomes	Finnish European (FIN)	10,824	0.01	0.001	_
gnomAD v2.1	Exomes	Non-Finnish European (NFE)	56,885	0.01	0.001	_
gnomAD v2.1	Exomes	Ashkenazi (ASJ)	5,040	0.01	0.001	_
gnomAD v2.1	Exomes	Other (OTH)	3,070	0.01	0.002	_
1000 Genomes	Genomes	African (AFR)	669	0.01	0.002	0.002
1000 Genomes	Genomes	Admixed American (AMR)	352	0.01	0.002	0.002
1000 Genomes	Genomes	East Asian (EAS)	515	0.01	0.002	0.002
1000 Genomes	Genomes	European (EUR)	505	0.01	0.002	0.002
1000 Genomes	Genomes	South Asian (SAS)	494	0.01	0.002	0.002
Genomics England internal frequencies	Genomes	GEL	6628	0.01	0.001	_

Note, the South Asian (SAS) population from gnomADv2 was excluded from these frequency filters due to a known technical error.

Supplementary Materials

Supplementary Table 3 - Numbers of probands assigned to each genetically inferred ancestry group, the reference populations merged to create them, the place of birth of individuals that comprised that reference group (taken from Prive et al. 2022), and comparisons with 16+1 and other reported parental ethnicities.

Ancestry group	#	Initial reference groups merged	Place of birth of reference individuals	Proband reported 16+1 ethnicities	Other reported parental ethnicities
European	23623	Scandinavia, United Kingdom, Ireland, Finland, Europe South West, Europe South East, Europe North East, Italy	United Kingdom, Ireland, Denmark, Norway, Sweden, Germany, Netherlands, Iceland, Poland, Ukraine, Latvia, Germany, Kazakhstan, Lithuania, Romania, Serbia/Montenegro, Bosnia and Herzegovina, Bulgaria, Croatia, Hungary, Italy, Macedonia, Russia, Spain, Portugal, France, Gibraltar	British, Irish, Any other ethnic group, Any other mixed background, Any other White background, White and Asian, White and Black African, White and Black Caribbean	Albanian, American, Australian, British, Bulgarian, Canadian, Cyprus, Danish, Dutch, Eastern European, European, French, German, Greek, Greek Cypriot, Hungarian, Irish, Italian, Jewish, Kosovo, Latvian, Lithuanian, Maltese, New Zealand, Northern Irish, Norwegian, Polish, Portuguese, Romanian, Russian, Scottish, Slovakian, South African, Spanish, Swedish, Turkish, Ukraine, Welsh
South Asian	3030	Bangladesh, Pakistan, Sri Lanka	Bangladesh, Pakistan, Sri Lanka	Bangladeshi, British, Indian, Pakistani, White and Asian, Any other Asian background, Any other ethnic group, Any other mixed background	Afghanistan, Gujarati, Indian, Kashmir, Mauritius, Sri Lanka
Remaining participants	941	Unassigned, Japan, Philippines, South America	Japan, Philippines, Peru	African, Any other Asian background, Any other Black background, Any other ethnic group, Any other mixed background, Any other White background, British, Caribbean, White and Asian, White and Black African, White and Black Caribbean	Jamaican, Japanese, Philippines

Supplementary Materials

Ancestry group	#	Initial reference groups merged	Place of birth of reference individuals	Proband reported 16+1 ethnicities	Other reported parental ethnicities
West African*	679	Africa West, Africa South	Zimbabwe, Uganda, South Africa, Zambia, Malawi, Tanzania, Congo, Nigeria, Ghana, Sierra Leone, Caribbean, Ivory Coast, Liberia, The Guianas, Togo	African, Any other Black background, Any other ethnic group, Caribbean, White and Black African, White and Black Caribbean	Barbados, Ghana, Jamaica, Jamaican, Nigeria
Middle Eastern	452	Middle East	Iraq, Iran, Turkey, Syria	Any other Asian background, Any other ethnic group, Any other mixed background, Any other White background, British, White and Asian	Cypriot, Cyprus, Iran, Iranian, Iraq, Iraqi, Kurdish, Lebanese, Romanian, Syrian, Turkish
Ashkenazi	250	Ashkenazi	United Kingdom, USA, Israel, Hungary, Ireland, France, Canada, Russia	Any other White background, British	Ashkenazi Jewish, German, Jewish, Russian
East African	159	Africa East	Somalia, Ethiopia, Sudan, Eritrea	African, Any other Black background, Any other ethnic group	Somalian, Sudan, Ethiopian, Eritrean
North African	147	Africa North	Algeria, Morocco, Egypt, Libya, Tunisia	Any other Asian background, Any other ethnic group, Any other White background	Algerian, Libya, Yemen
East Asian	124	Asia East	Hong Kong, Malaysia, China, Singapore, Vietnam, Indonesia, Taiwan, Thailand, Brunei, Macau (Macao)	Chinese, Any other Asian background, Any other ethnic group	Hong Kong

*West African was retained as an ancestry group label despite the broad geographic distribution of reference groups, reflecting shared genetic ancestry arising from historical migrations out of West African across sub-Saharan Africa (e.g. the Bantu expansion).

Supplementary Table 4 - Within-group KING kinship coefficient summary statistics per ancestry group within the UK COVID-19 Genomics Study dataset following the exclusion of individuals with a kinship coefficient ≤ 0.0442 (removal of 3rd degree relatives and below).

Ancestry group	#	Mean within-group kinship coefficient (sd.)	Max. within-group kinship coefficient	Number of pairwise comparisons
European	27227	-0.0032 (0.00951)	0.04399	370641151
South Asian	2850	-0.0297 (0.03474)	0.0433	4059825
West African	1282	-0.013 (0.01101)	0.04302	821121
Remaining participants	1042	-0.0901 (0.06776)	0.04129	542361
Middle Eastern	442	-0.0277 (0.02792)	0.04065	97461
East Asian	332	-0.0115 (0.0133)	0.04156	54946
Ashkenazi	306	-0.0022 (0.00529)	0.02634	46665
North African	129	-0.0213 (0.03013)	0.01297	8256
East African	114	-0.0229 (0.02014)	0.02427	6441

Supplementary Materials

Supplementary Table 5 - Population sizes and 95% filtering allele frequency (FAF) thresholds derived from the UK COVID-19 Genomics Study and retrospectively integrated into the automated variant prioritisation pipeline.

Sequencing	Ancestry group	#	FAF threshold Dominant	Effective AF threshold* Dominant	FAF threshold Recessive	Effective AF threshold* Recessive
Genomes	European	27227	0.001	0.001249	0.01	0.010725
Genomes	South Asian	2850	0.001	0.00193	0.01	0.012456
Genomes	West African	1282	0.001	0.00234	0.01	0.013651
Genomes	Remaining participants	1042	0.001	0.002879	0.01	0.014395
Genomes	Middle Eastern	442	0.001	0.004525	0.01	0.016968
Genomes	East Asian	332	0.001	0.004518	0.01	0.018072
Genomes	Ashkenazi	306	0.001	0.004902	0.01	0.017974
Genomes	North African	129	0.001	0.007752	0.01	0.023256
Genomes	East African	114	0.001	0.008772	0.01	0.026316

*Effective allele frequency (AF) thresholds shown here correspond to the observed AF at which the FAF falls below the filtering thresholds for dominant and recessive inheritance models. Thresholds were applied per ancestry group as shown.

Supplementary Table 6 - Population sizes and 95% filtering allele frequency (FAF) thresholds derived from the external gnomADv4.1 reference database and retrospectively integrated into the automated variant prioritisation pipeline.

Sequencing	Population	#	FAF threshold Dominant	Effective AF threshold* Dominant	FAF_{95} threshold Recessive	Effective AF Threshold* Recessive
Genomes & Exomes	Non-Finnish European	590031	0.001	0.001049	0.01	0.010153
Genomes & Exomes	South Asian	45548	0.001	0.001186	0.01	0.010561
Genomes & Exomes	Remaining participants	31256	0.001	0.001212	0.01	0.010614
Genomes & Exomes	African/African American	37545	0.001	0.001232	0.01	0.010686
Genomes & Exomes	Admixed American	30019	0.001	0.001233	0.01	0.010693
Genomes & Exomes	East Asian	22448	0.001	0.00127	0.01	0.010803
Genomes & Exomes	Ashkenazi	14804	0.001	0.001351	0.01	0.011011
Genomes & Exomes	Middle Eastern	3031	0.001	0.001815	0.01	0.012372

*Effective allele frequency (AF) thresholds shown here correspond to the observed AF at which the FAF falls below the filtering thresholds for dominant and recessive inheritance models. Thresholds were applied per ancestry group as shown.

Supplementary Table 7 - Summary statistics of variant counts returned by the automated variant prioritisation pipeline by genetically inferred ancestry group.

Outcomes	Overall	European	East Asian	Middle Eastern	Ashkenazi	South Asian	North African	East African	West African	Remaining participants
**Prioritised variants**										
Total	56642	40368	333	1063	323	8551	398	955	2159	2492
Unique*	37870	26320	327	1026	266	6858	387	858	1921	2294
median (IQR) per proband	1 (0–2)	1 (0–2)	1 (0–3)	1 (0–3)	0 (0–1)	2 (0–4)	2 (1–3)	4 (1–8)	2 (0–5)	1 (0–4)
min-max per proband	0–38	0–38	0–29	0–18	0–11	0–32	0–21	0–38	0–23	0–29
Probands with >0 / total probands (%)	17790 / 29405 (60.5%)	13665 / 23623 (57.8%)	90 / 124 (72.6%)	326 / 452 (72.1%)	124 / 250 (49.6%)	2238 / 3030 (73.9%)	112 / 147 (76.2%)	129 / 159 (81.1%)	477 / 679 (70.3%)	629 / 941 (66.8%)
**Candidate variants** (outside virtual panels)										
Total	5662419	4192244	33236	123066	39356	700966	36741	82551	241934	212325
Unique*	1955566	1379100	25324	86526	18893	348833	28082	46573	137909	147636
median (IQR) per proband	226 (43–283)	224 (36–273)	338 (66–386)	364 (47–423)	170 (116–211)	209 (41–400)	236 (45–416)	620 (298–758)	412 (208–480)	235 (36–356)
min-max per proband	0–1073	0–582	6–502	3–561	3–344	0–582	5–525	4–953	5–1073	1–980
Probands with >0 / total probands (%)	29398 / 29405 (~100.0%)	23618 / 23623 (~100.0%)	124 / 124 (100.0%)	452 / 452 (100.0%)	250 / 250 (100.0%)	3028 / 3030 (99.9%)	147 / 147 (100.0%)	159 / 159 (100.0%)	679 / 679 (100.0%)	941 / 941 (100.0%)

Supplementary Table 8 - Negative binomial mixed effects regression model output (prioritised variants as counts variable).

	Number of prioritised variants returned			
	Unadjusted model		Adjusted model	
	IRR (95% CI)	p value	IRR (95% CI)	p value
**Ancestry group**				
European	1 (ref)	—	1 (ref)	—
Ashkenazi	0.75 (0.62, 0.89)	0.0015	0.75 (0.63, 0.89)	0.001
East African	3.13 (2.60, 3.75)	<0.0001	2.77 (2.33, 3.29)	<0.0001
East Asian	1.52 (1.21, 1.90)	0.0002	1.41 (1.14, 1.74)	0.0015
Middle Eastern	1.34 (1.19, 1.51)	<0.0001	1.29 (1.15, 1.45)	<0.0001
North African	1.52 (1.24, 1.86)	<0.0001	1.38 (1.13, 1.68)	0.0012
Remaining participants	1.47 (1.35, 1.60)	<0.0001	1.41 (1.30, 1.53)	<0.0001
South Asian	1.55 (1.47, 1.63)	<0.0001	1.47 (1.39, 1.56)	<0.0001
West African	1.88 (1.71, 2.07)	<0.0001	1.79 (1.63, 1.96)	<0.0001
**Age at consent (per year)**				
—	—	—	0.99 (0.99, 0.99)	<0.0001
**Sex**				
Male	—	—	1 (ref)	—
Female	—	—	0.93 (0.90, 0.96)	<0.0001
**Family structure**				
Singleton	—	—	1 (ref)	—
Family duo	—	—	0.88 (0.84, 0.93)	<0.0001
Family trio	—	—	0.52 (0.49, 0.54)	<0.0001
Other	—	—	0.60 (0.56, 0.63)	<0.0001
**Family disease affection**				
Only proband affected	—	—	1 (ref)	—
Unknown	—	—	1.89 (1.79, 1.99)	<0.0001
Family member affected	—	—	0.94 (0.90, 0.98)	0.0015
**Predicted consanguinity**				
Nonconsanguineous	—	—	1 (ref)	—
Consanguineous	—	—	1.25 (1.16, 1.34)	<0.0001
Possibly consanguineous	—	—	1.13 (0.98, 1.29)	0.0865
**Random effects**				
• Disease group (σ)	0.55	—	0.56	—
• Handling GMC (σ)	0.11	—	0.14	—

Supplementary Table 9 - Summary statistics of outcomes recorded in the clinical scientist outcomes questionnaires (diagnostic variants, VUS) for 100kGP probands stratified by genetically inferred ancestry groups.

Outcomes	European	East Asian	Middle Eastern	Ashkenazi	South Asian	North African	East African	West African	Remaining participants
**Diagnostic Yield**									
Diagnosis* recorded (%)	4335 (18.4%)	36 (29.0%)	107 (23.7%)	48 (19.2%)	764 (25.2%)	38 (25.9%)	32 (20.1%)	122 (18.0%)	180 (19.1%)
No diagnosis recorded (%)	19288 (81.6%)	88 (71.0%)	345 (76.3%)	202 (80.8%)	2266 (74.8%)	109 (74.1%)	127 (79.9%)	557 (82.0%)	761 (80.9%)
**VUS**									
median VUS recorded (IQR)	0 (0-0)	0 (0-0)	0 (0-0)	0 (0-0)	0 (0-0)	0 (0-0)	0 (0-0)	0 (0-0)	0 (0-0)
min-max per proband	0-9	0-4	0-4	0-2	0-6	0-2	0-5	0-6	0-7
VUS recorded (%)	2232 (9.4%)	13 (10.5%)	37 (8.2%)	8 (3.2%)	375 (12.4%)	8 (5.4%)	22 (13.8%)	74 (10.9%)	120 (12.8%)
No VUS recorded (%)	21391 (90.6%)	111 (89.5%)	415 (91.8%)	242 (96.8%)	2655 (87.6%)	139 (94.6%)	137 (86.2%)	605 (89.1%)	821 (87.2%)

Supplementary Table 10 - Logistic mixed effects regression model output (diagnostic variant recorded = yes or no, as binary variable).

	Diagnostic yield			
	**Unadjusted model**		**Adjusted model**	
	**OR (95% CI)**	**p value**	**OR (95% CI)**	**p value**
**Ancestry group**				
European	1 (ref)	—	1 (ref)	—
Ashkenazi	1.16 (0.84, 1.61)	0.3643	1.20 (0.87, 1.67)	0.2641
East African	1.03 (0.69, 1.53)	0.8767	0.92 (0.62, 1.38)	0.6921
East Asian	1.76 (1.18, 2.62)	0.0053	1.77 (1.18, 2.64)	0.0054
Middle Eastern	1.28 (1.02, 1.60)	0.0324	1.05 (0.83, 1.33)	0.7085
North African	1.47 (1.01, 2.15)	0.0452	1.18 (0.80, 1.74)	0.4112
Remaining participants	1.02 (0.86, 1.20)	0.8466	0.97 (0.82, 1.15)	0.7366
South Asian	1.38 (1.26, 1.51)	<0.0001	1.10 (0.97, 1.23)	0.1258
West African	0.92 (0.75, 1.13)	0.4216	0.95 (0.77, 1.16)	0.5925
**Age at consent (per year)**				
—	—	—	1.00 (0.99, 1.00)	0.0002
**Sex**				
Male	—	—	1 (ref)	—
Female	—	—	1.12 (1.05, 1.19)	0.0003
**Family structure**				
Singleton	—	—	1 (ref)	—
Family duo	—	—	1.04 (0.93, 1.16)	0.5325
Family trio	—	—	1.44 (1.29, 1.60)	<0.0001
Other	—	—	0.83 (0.74, 0.93)	0.0016
**Family disease affection**				
Only proband affected	—	—	1 (ref)	—
Unknown	—	—	0.87 (0.76, 0.99)	0.0376
Family member affected	—	—	1.72 (1.59, 1.87)	<0.0001
**Predicted consanguinity**				
Nonconsanguineous	—	—	1 (ref)	—
Consanguineous	—	—	1.48 (1.28, 1.71)	<0.0001
Possibly consanguineous	—	—	1.32 (1.01, 1.72)	0.0404
**Random effects**				
• Disease group (σ)	0.59	—	0.60	—
• Handling GMC (σ)	0.17	—	0.17	—

Supplementary Table 11 - Logistic mixed effects regression model output (variant level sensitivity; prioritised diagnostic variants / total diagnostic variants).

	Sensitivity of prioritised variants			
	Unadjusted model		Adjusted model	
	OR (95% CI)	p value	OR (95% CI)	p value
**Ancestry group**				
European	1 (ref)	—	1 (ref)	—
Ashkenazi	0.61 (0.31, 1.19)	0.1478	0.55 (0.27, 1.09)	0.0859
East African	1.44 (0.55, 3.76)	0.4585	1.51 (0.57, 3.98)	0.4076
East Asian	1.34 (0.58, 3.10)	0.4974	1.35 (0.58, 3.15)	0.4846
Middle Eastern	0.69 (0.44, 1.09)	0.1118	0.76 (0.47, 1.23)	0.2607
North African	0.94 (0.42, 2.08)	0.8706	1.04 (0.46, 2.35)	0.9264
Remaining participants	0.80 (0.56, 1.14)	0.2142	0.85 (0.59, 1.21)	0.3604
South Asian	1.01 (0.83, 1.22)	0.9182	1.11 (0.86, 1.43)	0.4065
West African	0.85 (0.54, 1.32)	0.4589	0.85 (0.54, 1.33)	0.4747
**Age at consent (per year)**				
—	—	—	1.00 (1.00, 1.01)	0.0447
**Sex**				
Male	—	—	1 (ref)	—
Female	—	—	1.01 (0.89, 1.15)	0.8418
**Family structure**				
Singleton	—	—	1 (ref)	—
Family duo	—	—	0.78 (0.61, 1.00)	0.0535
Family trio	—	—	0.68 (0.54, 0.86)	0.0016
Other	—	—	0.48 (0.38, 0.62)	<0.0001
**Family disease affection**				
Only proband affected	—	—	1 (ref)	—
Unknown	—	—	1.28 (0.89, 1.84)	0.1892
Family member affected	—	—	1.14 (0.96, 1.37)	0.1451
**Predicted consanguinity**				
Nonconsanguineous	—	—	1 (ref)	—
Consanguineous	—	—	0.95 (0.70, 1.28)	0.7274
Possibly consanguineous	—	—	1.15 (0.65, 2.03)	0.6287
**Random effects**				
• Disease group (σ)	0.41	—	0.50	—
• Handling GMC (σ)	0.58	—	0.56	—

Supplementary Table 12 - Logistic mixed effects regression model output (variant level PPV; prioritised diagnostic variants / total prioritised variants).

	Positive predictive value (PPV) of prioritised variants			
	Unadjusted model		Adjusted model	
	OR (95% CI)	p value	OR (95% CI)	p value
**Ancestry group**				
European	1 (ref)	—	1 (ref)	—
Ashkenazi	1.31 (0.93, 1.84)	0.1222	1.45 (1.02, 2.06)	0.0382
East African	0.29 (0.20, 0.42)	<0.0001	0.32 (0.22, 0.46)	<0.0001
East Asian	1.09 (0.76, 1.57)	0.6272	1.16 (0.80, 1.68)	0.4253
Middle Eastern	0.70 (0.56, 0.88)	0.0021	0.68 (0.54, 0.86)	0.0013
North African	0.74 (0.51, 1.07)	0.1089	0.73 (0.50, 1.06)	0.1017
Remaining participants	0.60 (0.50, 0.71)	<0.0001	0.59 (0.50, 0.70)	<0.0001
South Asian	0.71 (0.65, 0.77)	<0.0001	0.65 (0.58, 0.73)	<0.0001
West African	0.44 (0.36, 0.54)	<0.0001	0.47 (0.39, 0.57)	<0.0001
**Age at consent (per year)**				
—	—	—	1.01 (1.00, 1.01)	<0.0001
**Sex**				
Male	—	—	1 (ref)	—
Female	—	—	1.18 (1.11, 1.25)	<0.0001
**Family structure**				
Singleton	—	—	1 (ref)	—
Family duo	—	—	1.25 (1.12, 1.39)	<0.0001
Family trio	—	—	2.69 (2.43, 2.97)	<0.0001
Other	—	—	1.46 (1.31, 1.63)	<0.0001
**Family disease affection**				
Only proband affected	—	—	1 (ref)	—
Unknown	—	—	0.48 (0.42, 0.55)	<0.0001
Family member affected	—	—	1.69 (1.57, 1.83)	<0.0001
**Predicted consanguinity**				
Nonconsanguineous	—	—	1 (ref)	—
Consanguineous	—	—	0.92 (0.80, 1.06)	0.2499
Possibly consanguineous	—	—	0.91 (0.70, 1.17)	0.4652
**Random effects**				
• Disease group (σ)	1.01	—	1.01	—
• Handling GMC (σ)	0.15	—	0.17	—

Supplementary Materials

Supplementary Table 13 - Logistic mixed effects regression model output (recorded VUS carrier status = yes or no, as binary variable).

	Recorded VUS frequency (per proband)			
	**Unadjusted model**		**Adjusted model**	
	**OR (95% CI)**	**p value**	**OR (95% CI)**	**p value**
**Ancestry group**				
European	1 (ref)	—	1 (ref)	—
Ashkenazi	0.63 (0.31, 1.31)	0.2188	0.66 (0.32, 1.35)	0.2530
East African	2.18 (1.34, 3.55)	0.0016	1.90 (1.17, 3.10)	0.0097
East Asian	1.31 (0.72, 2.42)	0.3789	1.28 (0.69, 2.36)	0.4308
Middle Eastern	1.23 (0.86, 1.75)	0.2584	1.04 (0.72, 1.51)	0.8342
North African	0.71 (0.34, 1.49)	0.3685	0.62 (0.30, 1.29)	0.2017
Remaining participants	1.49 (1.21, 1.84)	0.0001	1.45 (1.17, 1.78)	0.0005
South Asian	1.60 (1.41, 1.82)	<0.0001	1.35 (1.15, 1.59)	0.0003
West African	1.48 (1.14, 1.92)	0.0032	1.36 (1.05, 1.77)	0.0217
**Age at consent (per year)**				
—	—	—	1.00 (0.99, 1.00)	0.0016
**Sex**				
Male	—	—	1 (ref)	—
Female	—	—	0.90 (0.83, 0.98)	0.0167
**Family structure**				
Singleton	—	—	1 (ref)	—
Family duo	—	—	1.10 (0.95, 1.26)	0.2065
Family trio	—	—	0.64 (0.55, 0.74)	<0.0001
Other	—	—	0.66 (0.56, 0.78)	<0.0001
**Family disease affection**				
Only proband affected	—	—	1 (ref)	—
Unknown	—	—	1.06 (0.90, 1.25)	0.5035
Family member affected	—	—	1.10 (0.99, 1.23)	0.0775
**Predicted consanguinity**				
Nonconsanguineous	—	—	1 (ref)	—
Consanguineous	—	—	1.44 (1.18, 1.77)	0.0004
Possibly consanguineous	—	—	1.38 (0.96, 1.99)	0.0846
**Random effects**				
• Disease group (σ)	0.33	—	0.31	—
• Handling GMC (σ)	1.14	—	1.15	—

Supplementary Table 14 - Number and proportion of variants in the 100,000 Genomes Project cohort, stratified by genetic ancestry group, variant set, and allele frequency filtering source.

	Overall	European	East Asian	Middle Eastern	Ashkenazi	South Asian	North African	East African	West African	Remaining participants
**Prioritised variants**										
N total variants	56642	40368	333	1063	323	8551	398	955	2159	2492
N total filtered using COVID-19 Genomic Study AFs (% of total)	1777 (3.1%)	522 (1.3%)	11 (3.3%)	46 (4.3%)	13 (4.0%)	672 (7.9%)	29 (7.3%)	232 (24.3%)	101 (4.7%)	151 (6.1%)
N total filtered using gnomADv4.1 AFs (% of total)	2600 (4.6%)	1167 (2.9%)	9 (2.7%)	77 (7.2%)	23 (7.1%)	1028 (12.0%)	27 (6.8%)	59 (6.2%)	93 (4.3%)	117 (4.7%)
N total filtered by both sets of AFs (% of total)	1187 (2.1%)	353 (0.9%)	2 (0.6%)	32 (3.0%)	12 (3.7%)	622 (7.3%)	15 (3.8%)	35 (3.7%)	57 (2.6%)	59 (2.4%)
N total unique variants	37870	26320	327	1026	266	6858	387	858	1921	2294
N unique variants filtered using COVID-19 Genomic Study AFs (% of total unique)	899 (2.4%)	264 (1.0%)	10 (3.1%)	42 (4.1%)	12 (4.5%)	338 (4.9%)	29 (7.5%)	195 (22.7%)	76 (4.0%)	134 (5.8%)
N unique variants filtered using gnomADv4.1 AFs (% of total unique)	1019 (2.7%)	413 (1.6%)	9 (2.8%)	69 (6.7%)	21 (7.9%)	542 (7.9%)	26 (6.7%)	54 (6.3%)	65 (3.4%)	97 (4.2%)
N unique variants filtered by both sets of AFs (% of total unique)	492 (1.3%)	154 (0.6%)	2 (0.6%)	28 (2.7%)	11 (4.1%)	295 (4.3%)	15 (3.9%)	31 (3.6%)	37 (1.9%)	51 (2.2%)
**Candidate variants** (outside applied panels)										
N total variants	5706800	4223650	33478	123936	39611	707908	37041	83354	243521	214301
N total filtered using COVID-19 Genomic Study AFs (% of total)	237405 (4.2%)	86022 (2.0%)	1170 (3.5%)	8249 (6.7%)	2005 (5.1%)	71096 (10.0%)	3777 (10.2%)	26371 (31.6%)	20525 (8.4%)	18190 (8.5%)
N total filtered using gnomADv4.1 AFs (% of total)	351498 (6.2%)	177618 (4.2%)	1558 (4.7%)	11644 (9.4%)	3632 (9.2%)	106103 (15.0%)	4052 (10.9%)	7530 (9.0%)	23503 (9.7%)	15858 (7.4%)

Supplementary Materials

	Overall	European	East Asian	Middle Eastern	Ashkenazi	South Asian	North African	East African	West African	Remaining participants
N total filtered by both sets of AFs (% of total)	154146 (2.7%)	52988 (1.3%)	587 (1.8%)	5264 (4.2%)	1570 (4.0%)	64218 (9.1%)	1934 (5.2%)	5018 (6.0%)	14330 (5.9%)	8237 (3.8%)
N total unique variants	1969846	1389157	25537	87183	19028	351953	28308	46922	138811	149138
N unique variants filtered using COVID-19 Genomic Study AFs (% of total unique)	32292 (1.6%)	14197 (1.0%)	845 (3.3%)	5154 (5.9%)	1024 (5.4%)	11611 (3.3%)	2853 (10.1%)	10097 (21.5%)	5494 (4.0%)	10448 (7.0%)
N unique variants filtered using gnomADv4.1 AFs (% of total unique)	35008 (1.8%)	18649 (1.3%)	840 (3.3%)	6853 (7.9%)	1660 (8.7%)	19097 (5.4%)	2872 (10.1%)	3444 (7.3%)	5098 (3.7%)	8242 (5.5%)
N unique variants filtered by both sets of AFs (% of total unique)	13815 (0.7%)	7258 (0.5%)	328 (1.3%)	3122 (3.6%)	727 (3.8%)	8811 (2.5%)	1379 (4.9%)	1981 (4.2%)	2542 (1.8%)	4182 (2.8%)

Counts are presented separately for all variants (total occurrences across participants) and unique variants (distinct variant sites counted once irrespective of the number of carriers). Variants are classified according to whether they exceed filtering thresholds based on allele frequencies derived from the COVID-19 Genomic Study, gnomAD v4.1, or both.

Supplementary Materials

Supplementary Table 15 - Classified VUS recorded in the outcomes questionnaire in that were initially prioritised and returned to clinical scientists during the 100kGP return of primary rare disease findings; yet were filtered out using the ancestry group stratified FAF filters from the UK COVID-19 Genomics Study.

Variant	Consequence	Gene	Applied PanelApp virtual gene panel(s)	Inheritance model	COVID19 Study Maximum AF	gnomADv4 Maximum AF	GEL Pipeline Maximum AF
chr1:11982112-AAAGT-A	Canonical splice-site (SO:0001575)	MFN2	Hereditary neuropathy	Dominant	0.033333 (East African)	–	–
chr1:173909791-G-T	Missense (SO:0001583)	SERPINC1	Inherited bleeding disorders	Dominant	0.007898 (South Asian)	0.007938 (South Asian)	0.000679 (GEL)
chr1:201060785-C-T	Missense (SO:0001583)	CACNA1S	Congenital myopathy	Dominant	0.006024 (East Asian)	0.000334 (East Asian)	0.000261 (gnomADv2 AFR)
chr1:201077002-C-G	Missense (SO:0001583)	CACNA1S	Congenital myopathy	Dominant	0.009117 (Remaining participants)	0.000816 (Remaining participants)	0.000377 (GEL)
chr1:241504239-G-C	Missense (SO:0001583)	FH	Adult solid tumours for rare disease	Dominant	0.002105 (South Asian)	0.001515 (South Asian)	–
chr2:47475052-A-G	Missense (SO:0001583)	MSH2	Adult solid tumours for rare disease, GI tract tumours	Dominant	0.011628 (North African)	0.008743 (Middle Eastern)	0.001276 (gnomADv2 OTH)
chr2:165162595-T-A	Missense (SO:0001583)	SCN3A	Epileptic encephalopathy, Familial Focal Epilepsies, Genetic epilepsy syndromes, Intellectual disability	Dominant	0.007752 (North African)	0.001972 (Middle Eastern)	6e-05 (gnomADv2 AMR)

Variant	Consequence	Gene	Applied PanelApp virtual gene panel(s)	Inheritance model	COVID19 Study Maximum AF	gnomADv4 Maximum AF	GEL Pipeline Maximum AF
chr2:178565038-T-C	Missense (SO:0001583)	TTN	Dilated Cardiomyopathy and conduction defects	Dominant	0.013158 (East African)	0.00024 (African/African American)	0.000226 (GEL)
chr2:178636754-T-C	Missense (SO:0001583)	TTN	Dilated Cardiomyopathy and conduction defects	Dominant	0.004535 (Middle Eastern)	0.002489 (Middle Eastern)	0.001 (1000G EUR)
chr2:190999619-C-A	Splice-region (SO:0001630)	STAT1	Primary immunodeficiency	Dominant	0.013158 (East African)	0.000509 (African/African American)	7.5e-05 (GEL)
chr2:203870802-G-A	Missense (SO:0001583)	CTLA4	Primary immunodeficiency disorders	Dominant	0.005656 (Middle Eastern)	0.002309 (Middle Eastern)	0.000379 (gnomADv2 NFE)
chr2:203870802-G-A	Missense (SO:0001583)	CTLA4	Primary immunodeficiency	Dominant	0.005656 (Middle Eastern)	0.002309 (Middle Eastern)	0.000379 (gnomADv2 NFE)
chr2:229808318-C-T	Missense (SO:0001583)	TRIP12	Intellectual disability	Dominant	0.017544 (East African)	0.000134 (African/African American)	7.5e-05 (GEL)
chr4:150872712-C-T	Missense (SO:0001583)	LRBA	A- or hypo-gammaglobulinaemia, Agranulocytosis, Combined B and T cell defect, Congenital neutropaenia, Multi-organ autoimmune diabetes, SCID	Recessive (compound het.)	0.109649 (East African)	0.002973 (Middle Eastern)	0.0023 (1000G AFR)

Variant	Consequence	Gene	Applied PanelApp virtual gene panel(s)	Inheritance model	COVID19 Study Maximum AF	gnomADv4 Maximum AF	GEL Pipeline Maximum AF
chr6:26092897-G-A	Missense (SO:0001583)	HFE	Undiagnosed metabolic disorders	Recessive	0.017719 (South Asian)	0.026338 (South Asian)	0.003772 (GEL)
chr6:152350676-A-G	Missense (SO:0001583)	SYNE1	Limb girdle muscular dystrophy	Dominant	0.017544 (East African)	0.000574 (Ashkenazi)	0.000813 (gnomADv2 ASJ)
chr7:156892978-C-T	Missense (SO:0001583)	LMBR1	Skeletal dysplasia	Dominant	0.001506 (European)	0.001212 (European)	0.000825 (gnomADv2 NFE)
chr8:10608115-G-C	Missense (SO:0001583)	RP1L1	Posterior segment abnormalities	Dominant	0.003619 (South Asian)	0.003148 (South Asian)	0.000679 (GEL)
chr8:10622700-C-T	Missense (SO:0001583)	RP1L1	Retinal disorders	Dominant	0.013158 (East African)	0.000164 (Middle Eastern)	3e-05 (gnomADv2 AMR)
chr9:127819664-C-T	Splice-region (SO:0001630)	ENG	Hereditary haemorrhagic telangiectasia	Dominant	0.008772 (East African)	0.000611 (Ashkenazi)	0.000628 (gnomADv2 ASJ)
chr10:100993505-A-C	Missense (SO:0001583)	TWNK	Inherited white matter disorders, Mitochondrial disorders, Undiagnosed metabolic disorders	Dominant	0.04386 (East African)	0.001155 (Middle Eastern)	8e-04 (1000G AFR)
chr11:19188209-C-A	Missense (SO:0001583)	CSRP3	Hypertrophic Cardiomyopathy	Dominant	0.005656 (Middle Eastern)	0.00022 (South Asian)	1.8e-05 (gnomADv2 NFE)
chr11:61962823-G-A	Missense (SO:0001583)	BEST1	Retinal disorders	Dominant	0.01938 (North African)	0.005774 (Middle Eastern)	0.001094 (gnomADv2 OTH)

Variant	Consequence	Gene	Applied PanelApp virtual gene panel(s)	Inheritance model	COVID19 Study Maximum AF	gnomADv4 Maximum AF	GEL Pipeline Maximum AF
chr11:62692395-C-T	Missense (SO:0001583)	BSCL2	Genetic epilepsy syndromes, Intellectual disability	Recessive	0.039474 (East African)	0.00076 (African/African American)	0.0015 (1000G AFR)
chr13:114324819-A-G	Missense (SO:0001583)	CHAMP1	Intellectual disability	Dominant	0.02193 (East African)	0.000493 (Middle Eastern)	–
chr15:31040113-C-T	Splice-region (SO:0001630)	TRPM1	Retinal disorders	Recessive (compound het.)	0.048246 (East African)	0.002225 (African/African American)	0.002222 (gnomADv2 AFR)
chr15:31061436-G-T	Splice-region (SO:0001630)	TRPM1	Retinal disorders	Recessive (compound het.)	0.048246 (East African)	0.001852 (African/African American)	0.002027 (gnomADv2 AFR)
chr16:2103764-G-A	Missense (SO:0001583)	PKD1	Cystic kidney disease	Recessive (compound het.)	0.012293 (European)	0.011498 (European)	0.008564 (gnomADv2 NFE)
chr16:2103764-G-A	Missense (SO:0001583)	PKD1	Cystic kidney disease	Recessive (compound het.)	0.012293 (European)	0.011498 (European)	0.008564 (gnomADv2 NFE)
chr16:2103764-G-A	Missense (SO:0001583)	PKD1	Cystic kidney disease, Rare multisystem ciliopathy disorders	Recessive (compound het.)	0.012293 (European)	0.011498 (European)	0.008564 (gnomADv2 NFE)
chr16:2103764-G-A	Missense (SO:0001583)	PKD1	Cystic kidney disease	Recessive (compound het.)	0.012293 (European)	0.011498 (European)	0.008564 (gnomADv2 NFE)
chr16:71067297-T-C	Missense (SO:0001583)	HYDIN	Primary ciliary disorders	Recessive (compound het.)	0.070093 (East African)	0.006721 (African/African American)	0.004074 (GEL)

Variant	Consequence	Gene	Applied PanelApp virtual gene panel(s)	Inheritance model	COVID19 Study Maximum AF	gnomADv4 Maximum AF	GEL Pipeline Maximum AF
chr16:89283717-C-T	Missense (SO:0001583)	ANKRD11	Intellectual disability	Dominant	0.013158 (East African)	0.000347 (Middle Eastern)	–
chr17:35101308-G-A	Missense (SO:0001583)	RAD51D	Adult solid tumours for rare disease, Familial breast cancer	Dominant	0.010181 (Middle Eastern)	0.000812 (South Asian)	0.000453 (GEL)
chr17:38328267-C-T	Missense (SO:0001583)	GPR179	Posterior segment abnormalities	Recessive (compound het.)	0.030702 (East African)	0.00033 (Middle Eastern)	–
chr17:38328600-C-T	Missense (SO:0001583)	GPR179	Posterior segment abnormalities	Recessive (compound het.)	0.030702 (East African)	0.00033 (Middle Eastern)	–
chr20:46726198-C-T	Missense (SO:0001583)	SLC2A10	Thoracic aortic aneurysm or dissection	Dominant	0.008772 (East African)	0.00066 (Middle Eastern)	0.000365 (gnomADv2 OTH)
chr20:62836323-C-T	Missense (SO:0001583)	COL9A3	Multiple Epiphyseal Dysplasia, Unexplained skeletal dysplasia	Dominant	0.008772 (East African)	0.00099 (Middle Eastern)	0.001 (1000G EUR)
chr21:46003588-C-T	Missense (SO:0001583)	COL6A1	Ehlers-Danlos syndromes	Dominant	0.003839 (Remaining participants)	0.000557 (East Asian)	8e-04 (1000G AFR)
chr22:26616165-G-T	Missense (SO:0001583)	CRYBB1	Cataracts	Dominant	0.003158 (South Asian)	0.003942 (South Asian)	0.000528 (GEL)
chrX:64193102-C-A	Missense (SO:0001583)	AMER1	Intellectual disability	Dominant	0.019868 (East African)	0.000689 (Middle Eastern)	0.0015 (1000G AFR)

Supplementary Materials

Variant	Consequence	Gene	Applied PanelApp virtual gene panel(s)	Inheritance model	COVID19 Study Maximum AF	gnomADv4 Maximum AF	GEL Pipeline Maximum AF
chrX:101397946-T-C	Missense (SO:0001583)	GLA	Hypertrophic Cardiomyopathy, Undiagnosed metabolic disorders	Dominant	0.004712 (South Asian)	0.004608 (South Asian)	0.000528 (GEL)
chrX:119872005-G-C	Missense (SO:0001583)	NDUFA1	Intellectual disability, Mitochondrial disorders, Structural basal ganglia disorders, Undiagnosed metabolic disorders	Recessive	0.011099 (European)	0.010282 (European)	0.009441 (gnomADv2 NFE)
chrX:154031004-A-G	Missense (SO:0001583)	MECP2	Intellectual disability	Dominant	0.032895 (East African)	0.000157 (African/African American)	0.000247 (gnomADv2 OTH)

Applied virtual gene panel name refers to the name of the PanelApp panel applied that this variant was originally prioritised under, whilst inheritance models refer to the underlying models (segregation patterns) that the variant was originally prioritised under. (GEL = Genomics England).

Supplementary Table 16 - Number and proportion of diagnostic SNVs and indels in the 100,000 Genomes Project cohort, stratified by ancestry group and pipeline return status.

Diagnostic variant(s)	Overall	European	East Asian	Middle Eastern	Ashkenazi	South Asian	North African	East African	West African	Remaining participants
**Prioritised variants**, N (% of group total)	5262 (80.9%)	4082 (81.2%)	35 (83.3%)	91 (76.5%)	42 (77.8%)	672 (79.8%)	33 (80.5%)	32 (86.5%)	119 (82.1%)	156 (78.0%)
**Candidate variants outside virtual panels**, N (% of group total)	741 (11.4%)	554 (11.0%)	6 (14.3%)	18 (15.1%)	<5	113 (13.4%)	<5	<5	14 (9.7%)	28 (14.0%)
**Not returned by the pipeline** N (% of group total)	503 (7.7%)	390 (7.8%)	<5	10 (8.4%)	9 (16.7%)	57 (6.8%)	5 (12.2%)	<5	12 (8.3%)	16 (8.0%)

Supplementary Table 17 - Number and proportion of diagnostic SNVs and indels in the 100,000 Genomes Project cohort, stratified by genetic ancestry group, pipeline return status, and Exomiser ranking.

Diagnostic variant(s)	Overall	European	East Asian	Middle Eastern	Ashkenazi	South Asian	North African	East African	West African	Remaining participants
**Prioritised variants**										
Exomiser top 5	4150 (78.9%)	3218 (78.8%)	29 (82.9%)	69 (75.8%)	34 (81.0%)	536 (79.8%)	27 (81.8%)	21 (65.6%)	91 (76.5%)	125 (80.1%)
Outside of Exomiser top 5	1112 (21.1%)	864 (21.2%)	6 (17.1%)	22 (24.2%)	8 (19.0%)	136 (20.2%)	6 (18.2%)	11 (34.4%)	28 (23.5%)	31 (19.9%)
**Candidate variants** (outside virtual panels)										
Exomiser top 5	494 (66.7%)	370 (66.8%)	<5	10 (55.6%)	<5	79 (69.9%)	<5	<5	8 (57.1%)	20 (71.4%)
Outside of Exomiser top 5	247 (33.3%)	184 (33.2%)	<5	8 (44.4%)	<5	34 (30.1%)	<5	<5	6 (42.9%)	8 (28.6%)
**Not returned by the pipeline**										
Exomiser top 5	119 (23.7%)	84 (21.5%)	_	5 (50.0%)	<5	16 (28.1%)	<5	_	<5	5 (31.2%)
Outside of Exomiser top 5	384 (76.3%)	306 (78.5%)	<5	5 (50.0%)	6	41 (71.9%)	<5	<5	9	11 (68.8%)

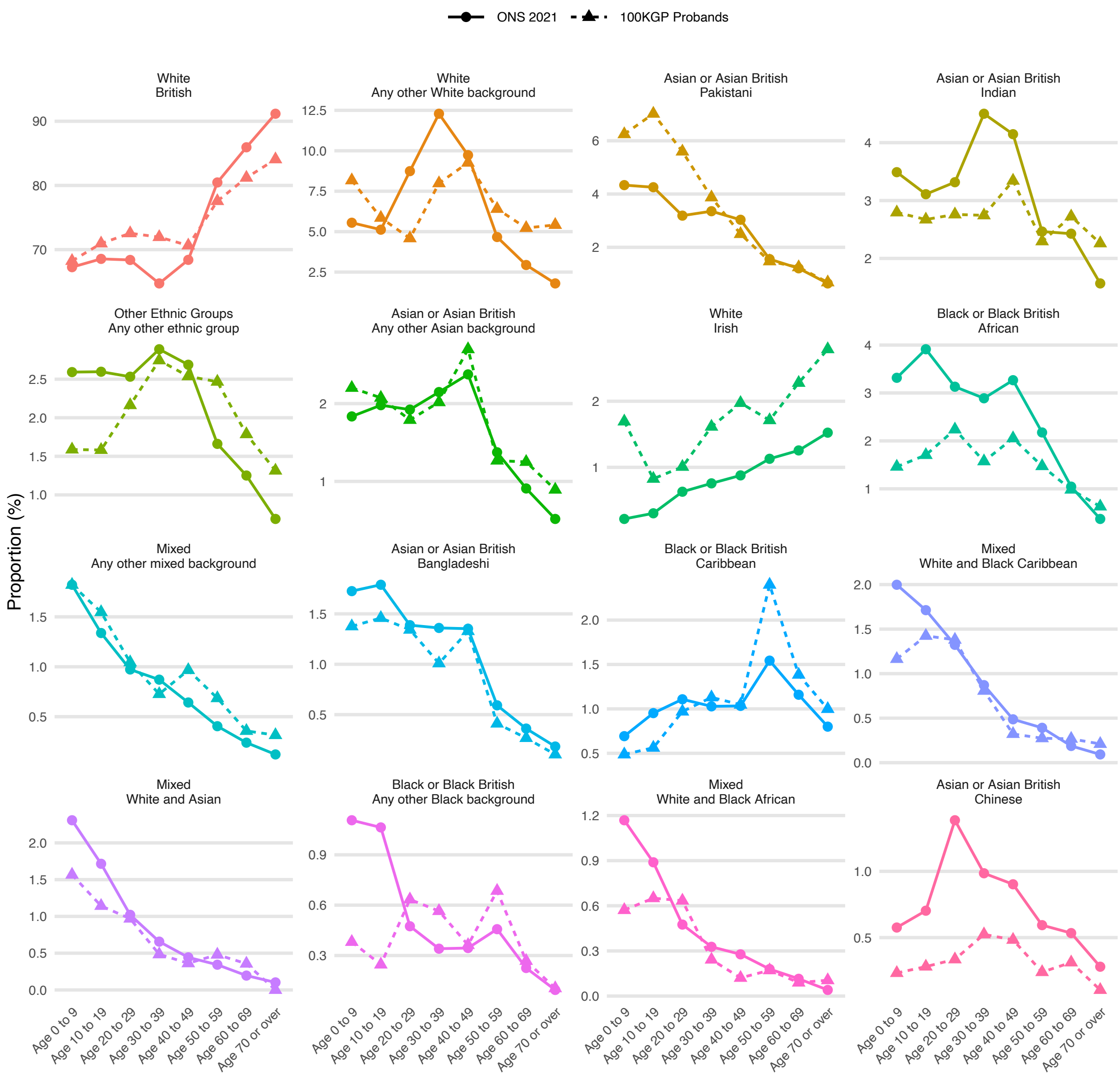

Supplementary Figure 1 - Ethnicity distribution of 100kGP probands.

Proportion of probands in the 100kGP reporting to each of 16+1 ethnic group categories (excluding individuals whose ethnic group was 'Not known' or was not stated) (dashed line) alongside the proportion of individuals self-reporting to each ethnic group as recorded in the ONS 2021 Census for England, stratified by age (in decades).

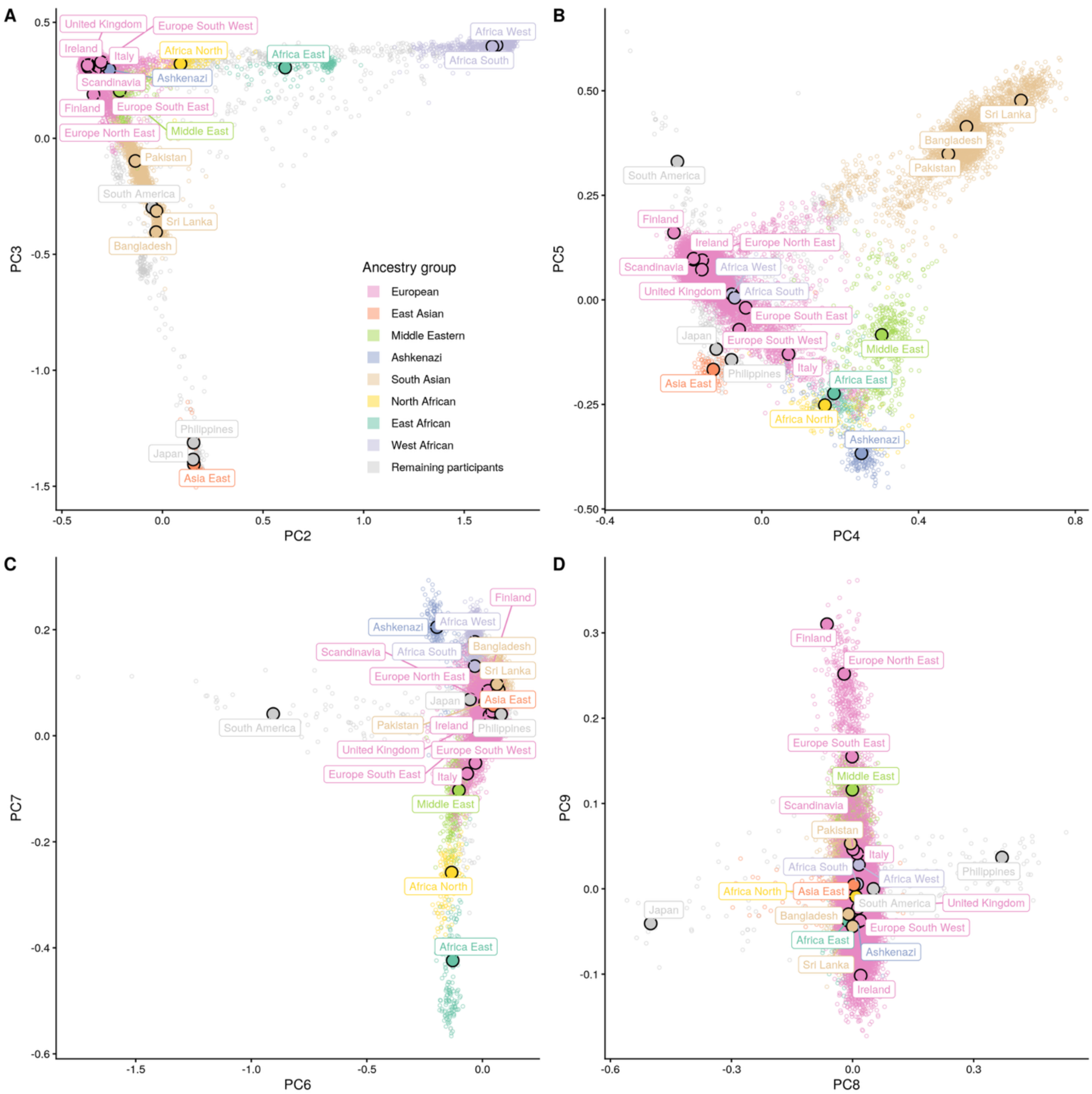

Supplementary *Figure 2* - Projection of 100kGP probands onto UK Biobank PCs (2-8)

Projection of 100kGP rare disease probands onto UK Biobank genetic principal component axes, coloured by assigned genetic ancestry group. Participants from the 100kGP are shown as points and were projected onto the principal component space derived from UK Biobank reference data, as described by Privé et al 2022[4]. Black outlined points with labels indicate the positions of 21 UK Biobank reference groups, projected onto the same axes using their allele frequency profiles. Reference groups merged for downstream analyses are labelled individually but coloured according to the broader merged ancestry group. Panels show PC2 versus PC3 (A), PC4 versus PC5 (B), PC6 versus PC7 (C), and PC8 versus PC9 (D).

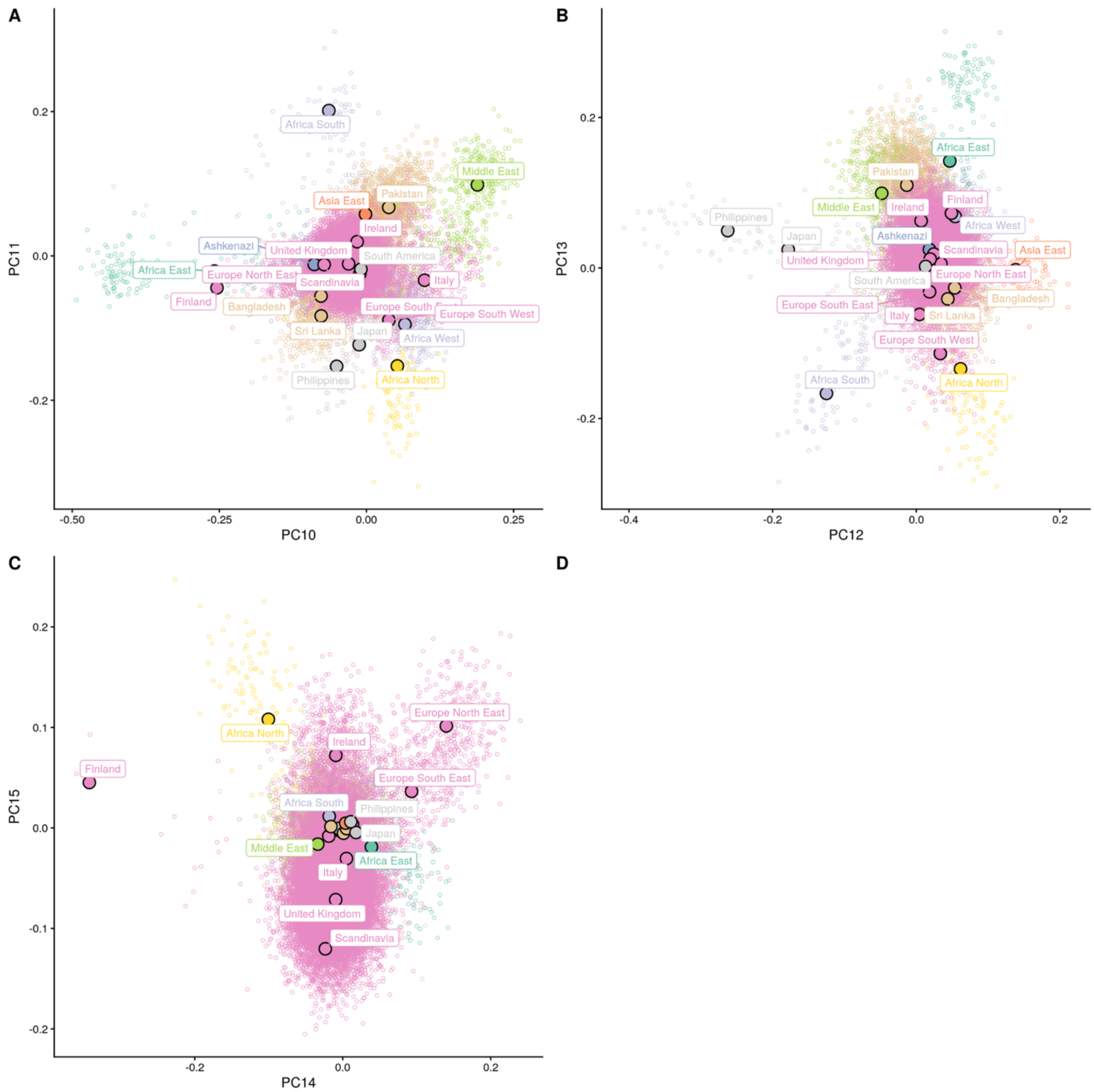

Supplementary Figure 3 - Projection of 100kGP probands onto the UK Biobank PCs (11-14)

Projection of 100kGP rare disease probands onto UK Biobank genetic principal component axes, coloured by assigned genetic ancestry group. Participants from the 100kGP are shown as points and were projected onto the principal component space derived from UK Biobank reference data, as described by Privé et al 2022[4]. Black outlined points with labels indicate the positions of 21 UK Biobank reference groups, projected onto the same axes using their allele frequency profiles. Reference groups merged for downstream analyses are labelled individually but coloured according to the broader merged ancestry group. Panels show PC10 versus PC11 (A), PC12 versus PC13 (B), and PC14 versus PC15 (C).

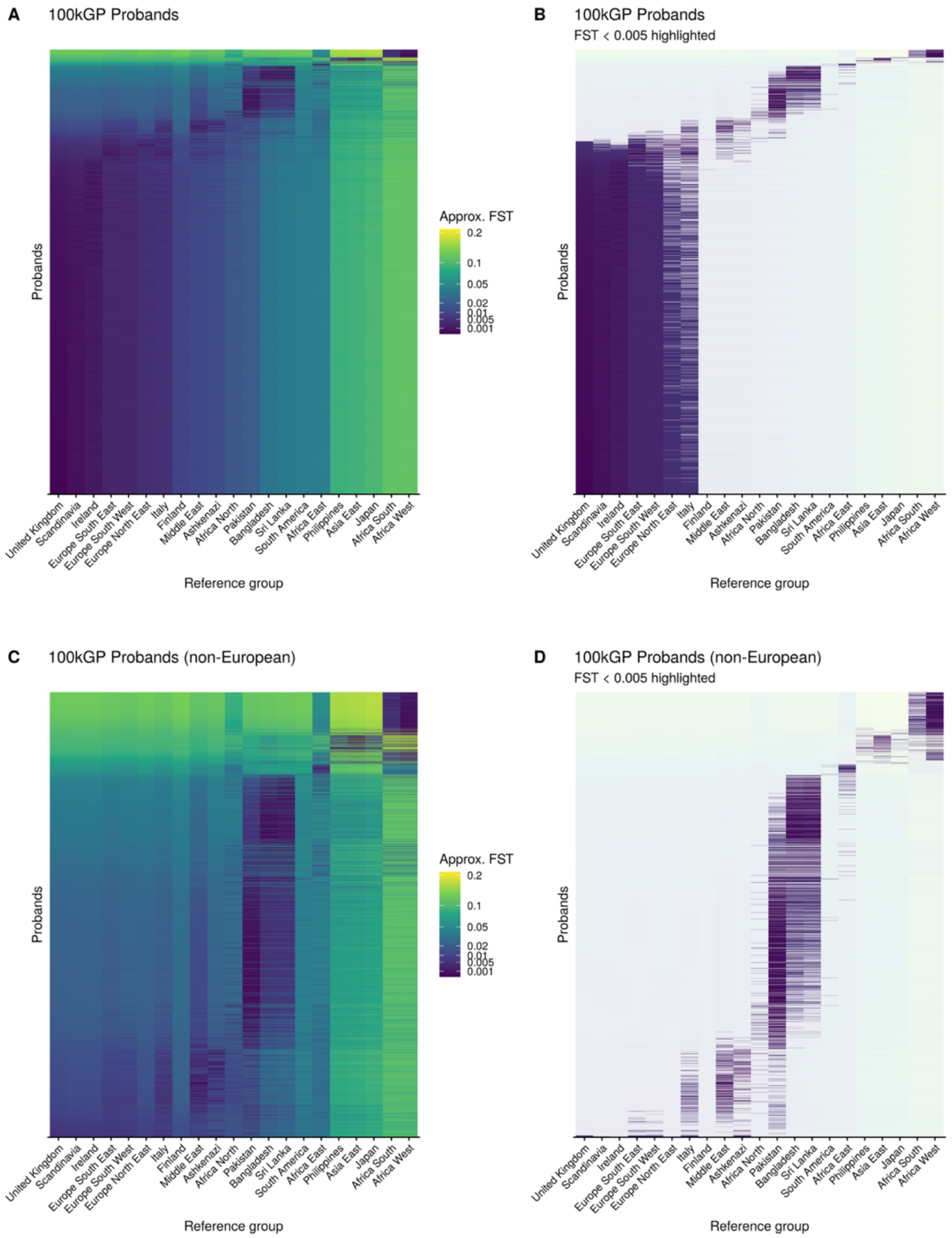

Supplementary Figure 4 - Approximate genetic distance between 100KGP probands and UK Biobank reference groups

For each proband, distances to 21 UK Biobank reference groups were calculated as squared Euclidean distances in principal component space to reference group centres, normalised by the maximum pairwise distance between reference groups and scaled to approximate F_{ST}. Rows represent probands and columns represent reference groups, with both ordered by increasing distance to the United Kingdom reference group. (A) Heatmap of approximate distance values across all proband–reference group comparisons. (B) Same data with cells corresponding to values < 0.005 highlighted, representing the threshold used for assignment to one or more reference groups prior to group consolidation. (C/D) as in A/B but excluding participants assigned to the European ancestry group (following consolidation).

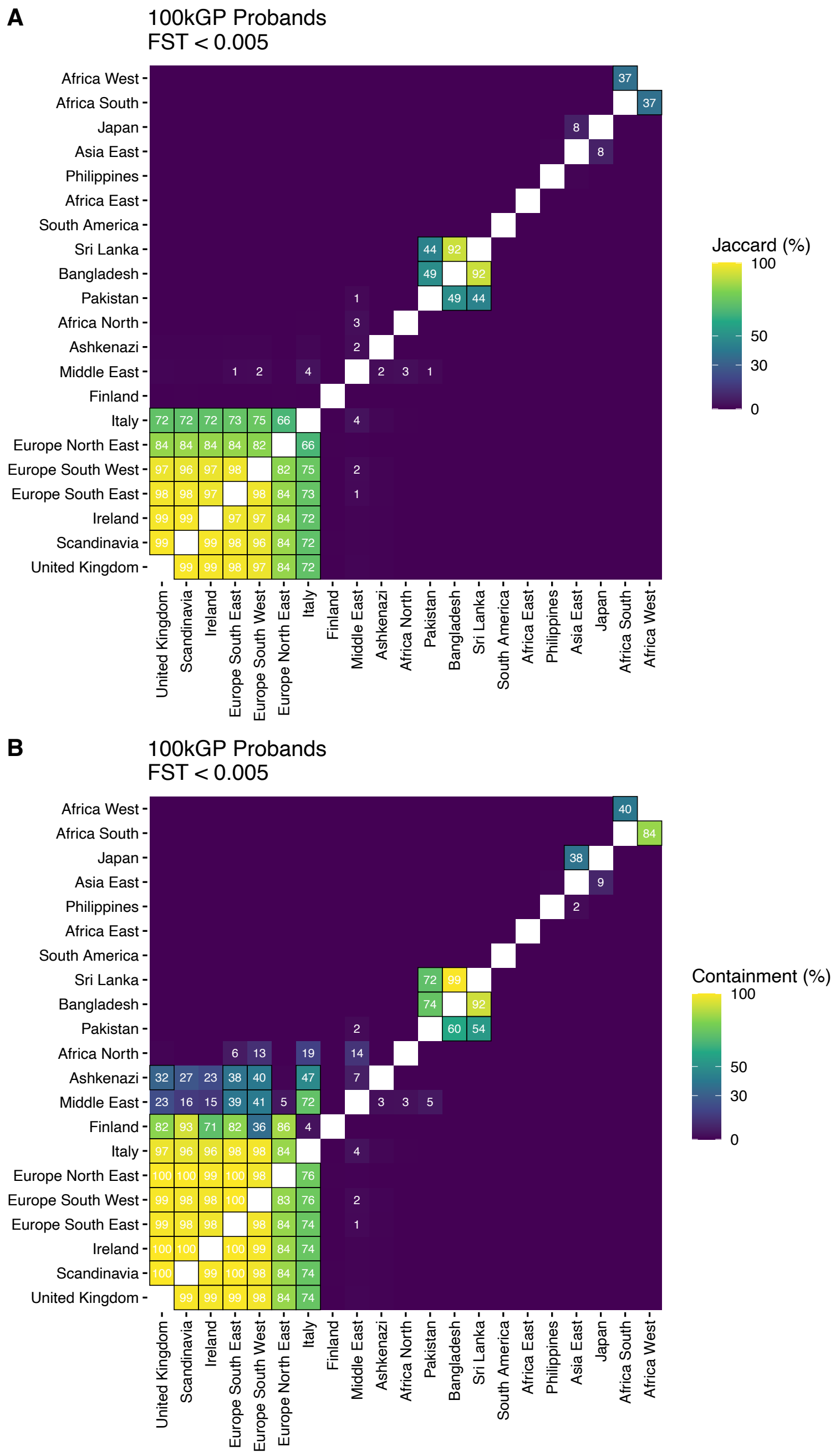

Supplementary Figure 5 - Pairwise overlap between UK Biobank reference groups among 100kGP probands assigned to one or more groups at an approximate F_{ST} threshold of < 0.005, before consolidation into broader ancestry groups

For each reference group, individuals with approximate $F_{ST} < 0.005$ were treated as a set. (A) Symmetric pairwise overlap measured using the Jaccard index, calculated as the percentage of individuals shared between two reference-group sets divided by the size of their union. (B) Directional pairwise containment, showing the percentage of individuals assigned to the reference group on the y-axis who were also assigned to the reference group (contained within) on the x-axis.

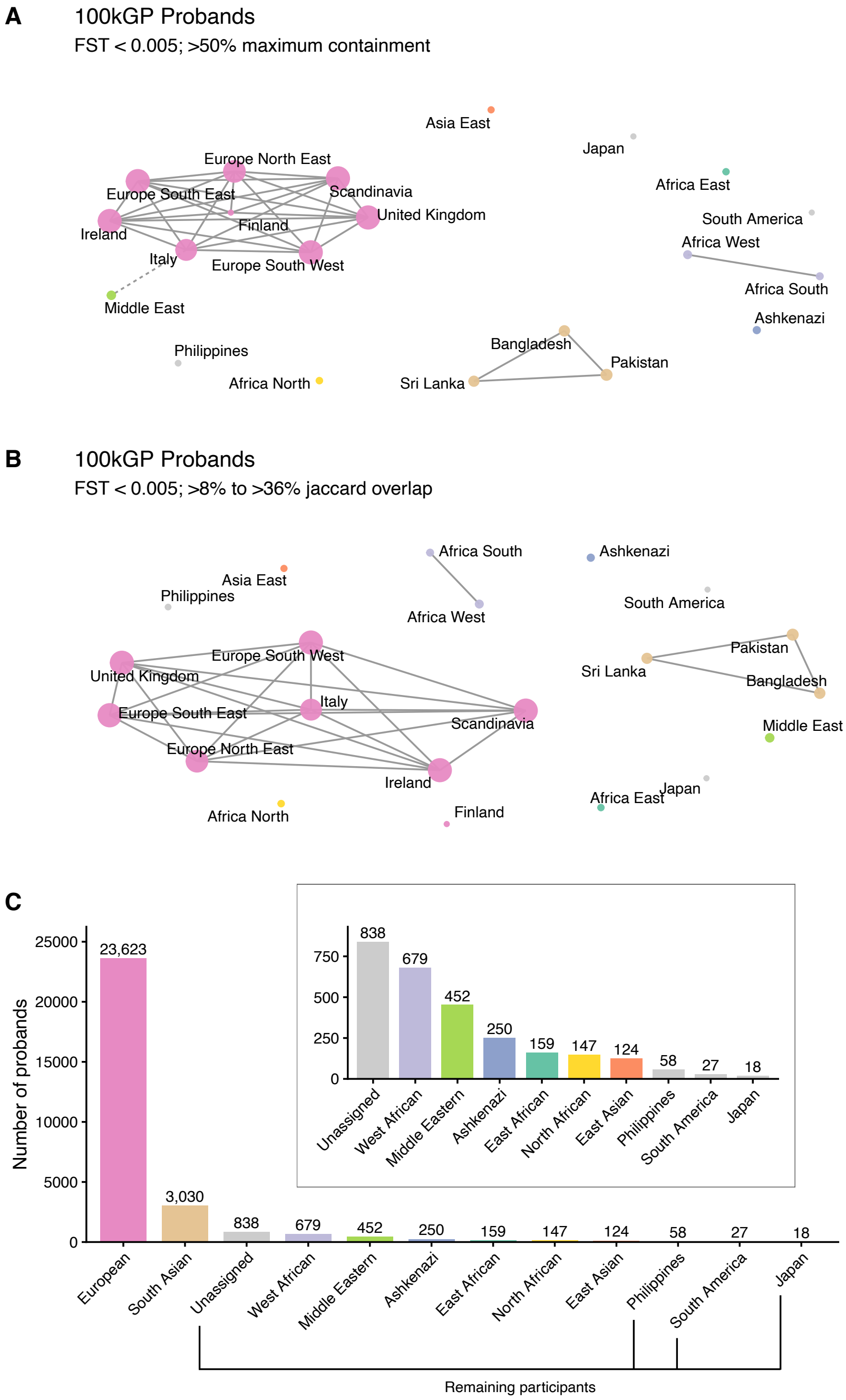

Supplementary Figure 6 – Graph-based consolidation of 100kGP probands into broader ancestry groups

(A) Network constructed using a maximum containment threshold (>50%) between groups following assignment at $F_{ST} < 0.005$. Nodes represent groups, with node size proportional to the number of individuals assigned. Edges connect sub-groups exceeding the specified containment threshold (in either direction). Dashed edges denote connections involving the Middle East group, which is retained as a separate group due to low Jaccard overlap despite >50% containment. (B) Network constructed using pairwise Jaccard overlap thresholds (8%–36%) under the same $F_{ST} < 0.005$ assignment. Edges connect subpopulations exceeding the specified overlap threshold. (C) Distribution of probands across 11 ancestry groups (and unassigned) following consolidation (merging of connected subgroups). Smaller groups (n<100) and unassigned individuals were combined into a single "Remaining participants" category.

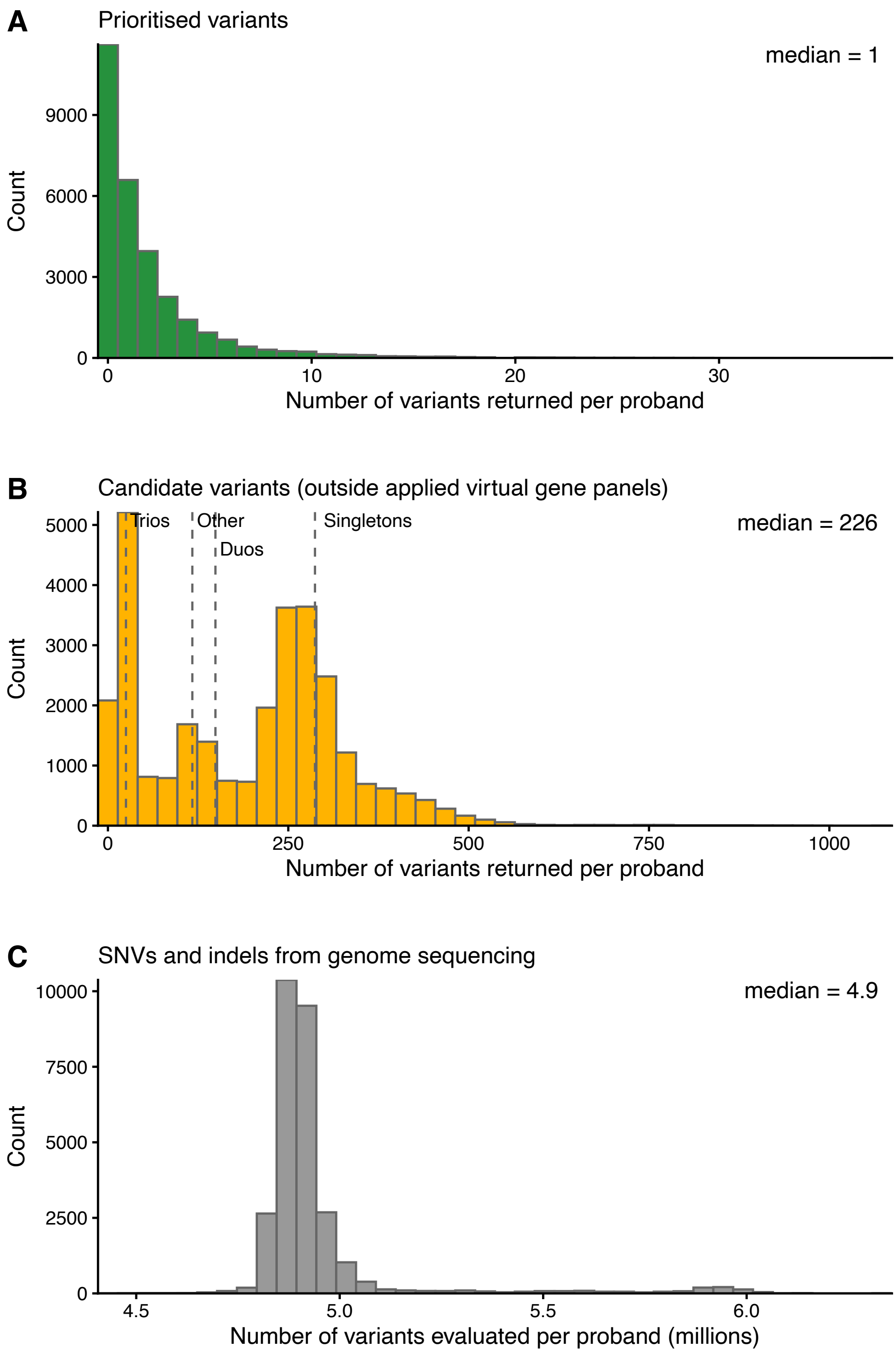

Supplementary Figure 7 - Histograms showing the numbers of variants returned by the automated pipeline per probands per variant type

(A) prioritised variants. (B) Candidate variants returned outside of the virtual gene panels (C) Total SNVs and indels evaluated by the pipeline.

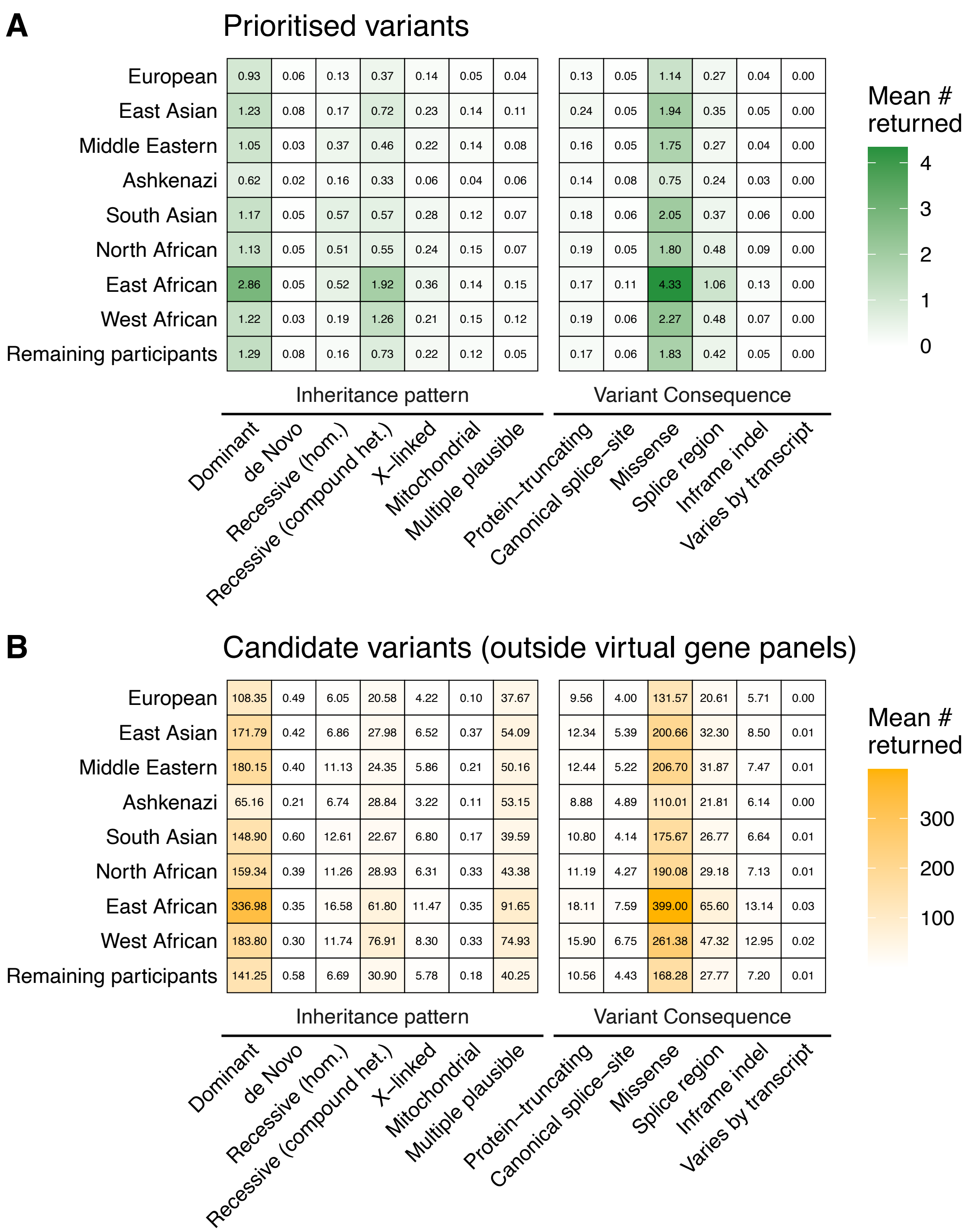

Supplementary Figure 8 - Heatmaps showing the mean number of variants returned by the automated pipeline per proband by ancestry group, inheritance pattern and molecular consequence.

(A) variants prioritised within the applied virtual gene panels, and (B) candidate variants outside those panels.

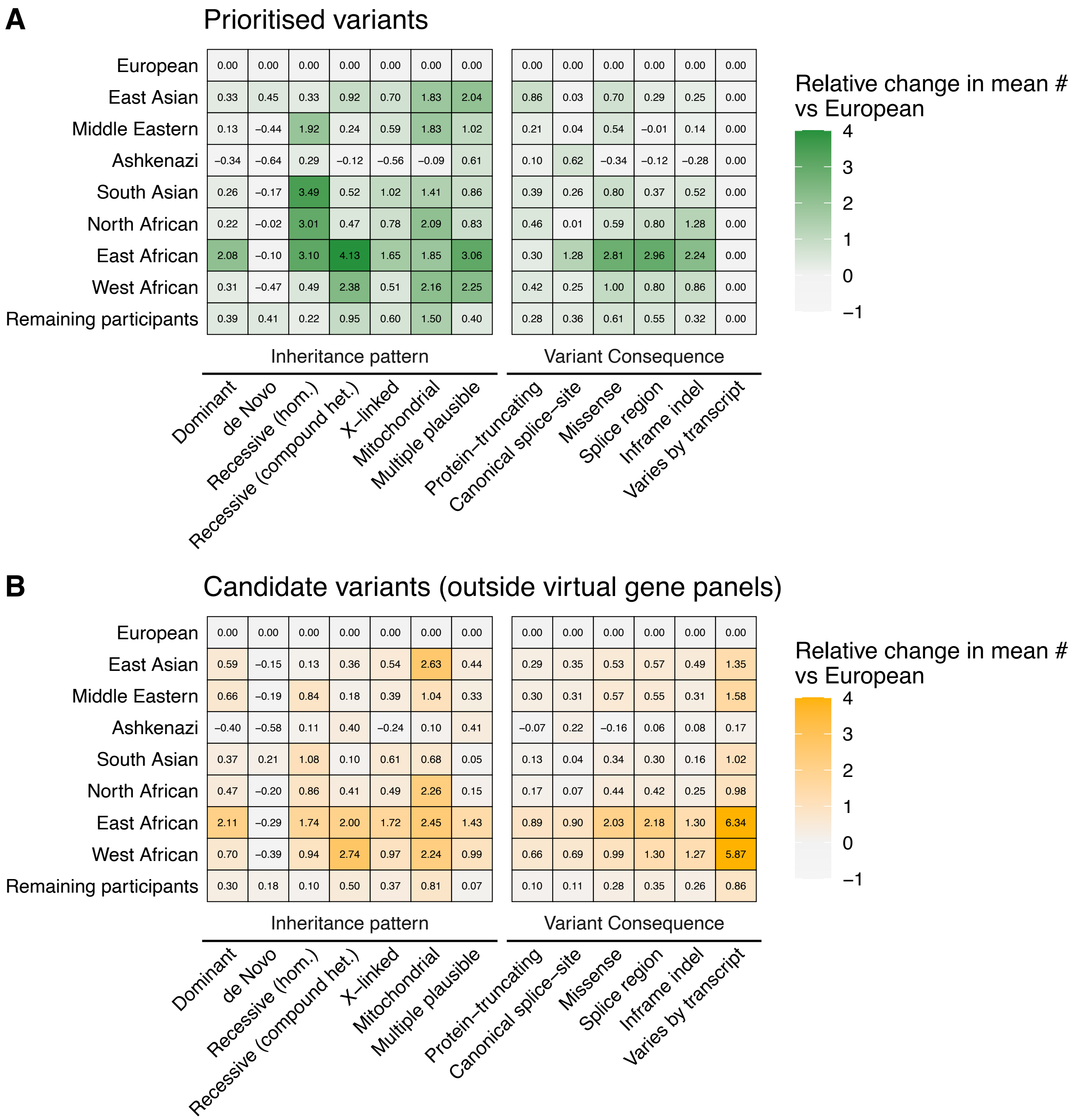

Supplementary Figure 9 - Heatmaps showing the relative change in the mean number of variants returned by the automated pipeline per proband, compared with the European ancestry group, stratified ancestry group, inheritance pattern, and molecular consequence.

(A) variants prioritised within the applied virtual gene panels, and (B) candidate variants outside those panels.

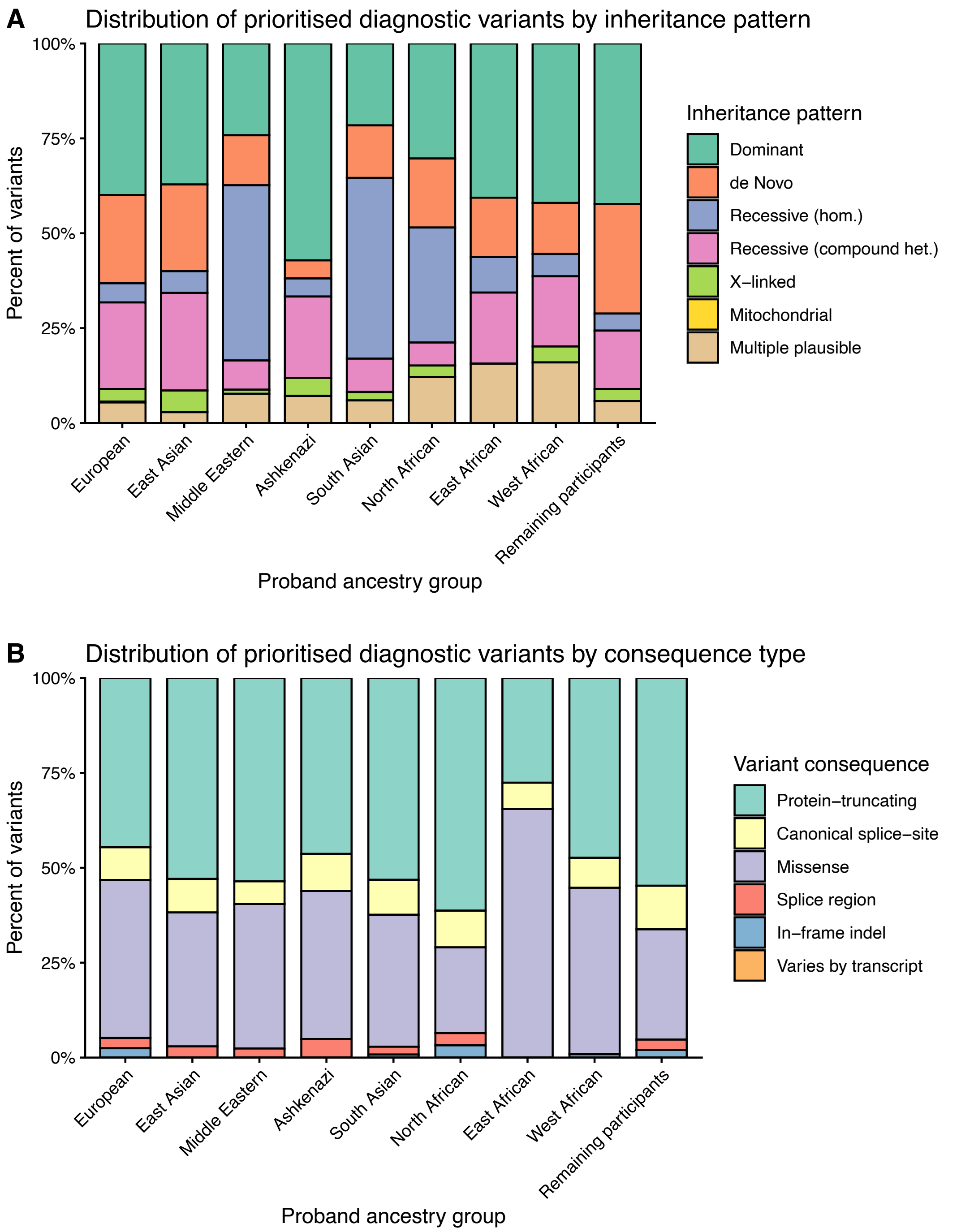

Supplementary Figure 10 – Distribution of prioritised diagnostic variants per ancestry group by inheritance patterna and variant consequence categories

Stacked bar chart showing, for each genetically inferred ancestry group, the proportion of reported diagnostic variants prioritised by the pipeline that fall into (A) each inferred inheritance pattern and (B) each variant consequence category.

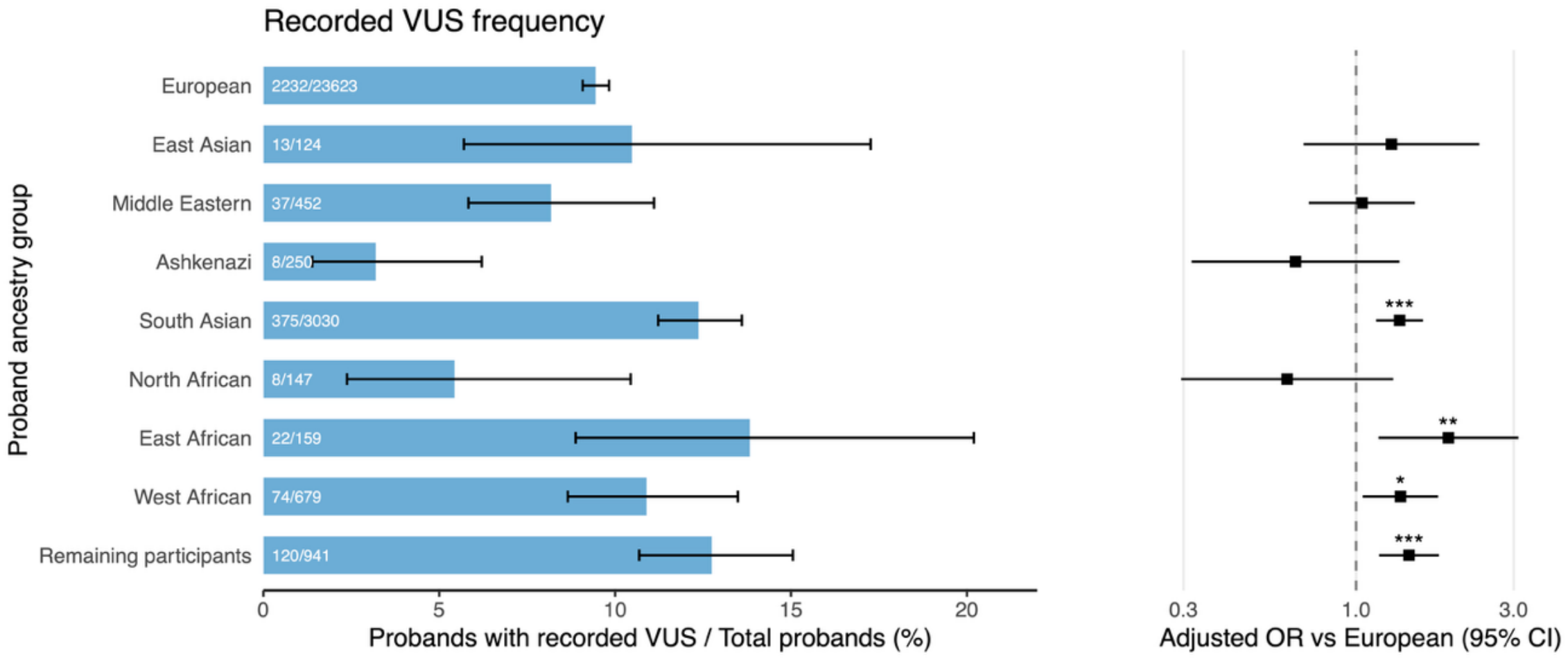

Supplementary Figure 11 – Differences in recorded variant of unknown significance (VUS) frequency, stratified by genetically inferred ancestry group.

Left: percentage of probands with at least one VUS (light blue bars), with numerator/denominator labels and 95% Clopper–Pearson confidence intervals (CIs; whiskers). Right: Forest plot with adjusted odds ratios (ORs) and 95% CIs from multivariable mixed-effects logistic regression of ancestry group on VUS carrier status, compared to the European ancestry group. Models adjusted for age, sex, family structure, family-member affection status, and consanguinity, with random intercepts for disease group and handling GMC (Methods). *** p<0·001; ** p<0·01; * p<0·05).

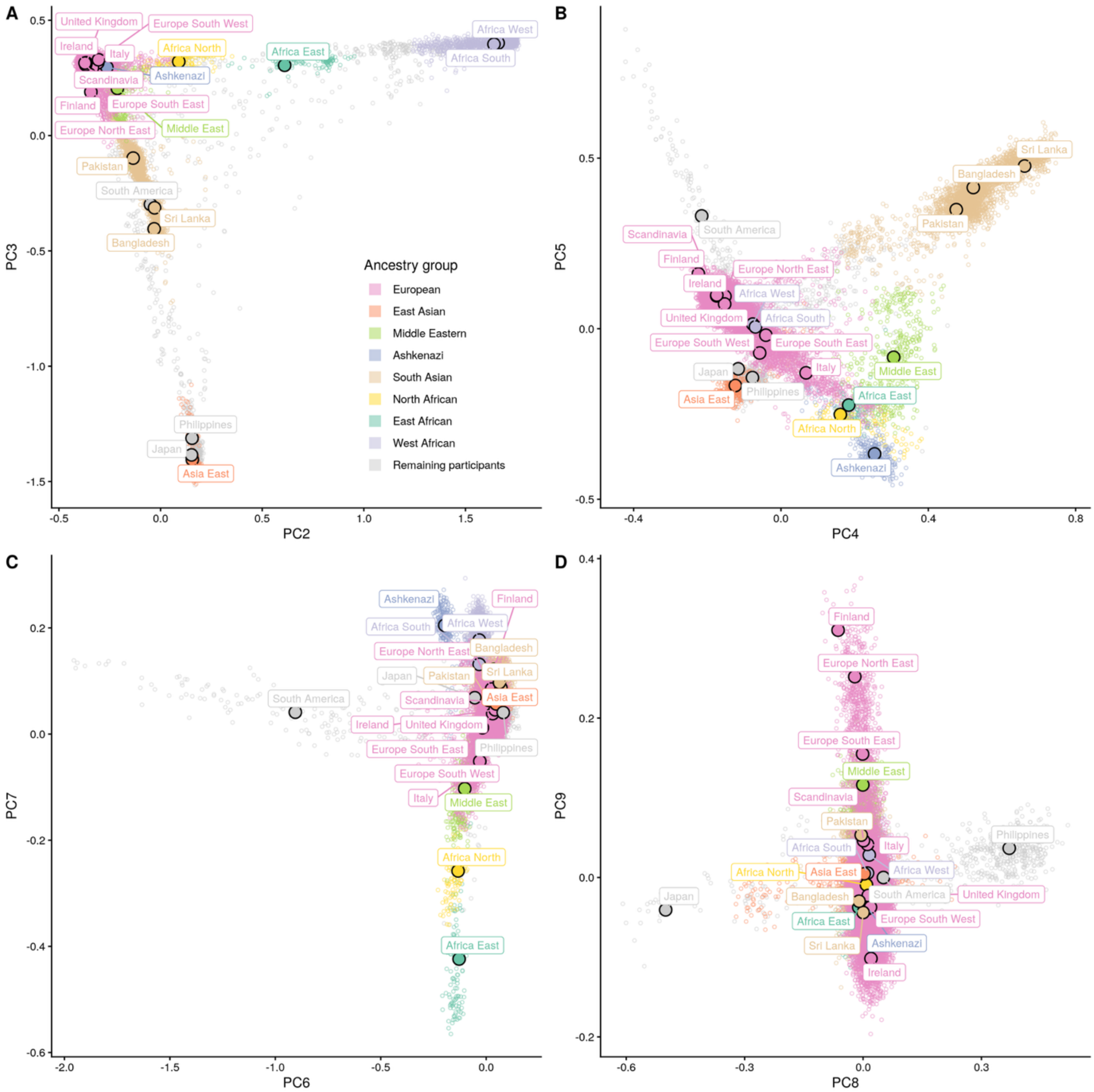

Supplementary Figure 12 - Projection of UK COVID-19 Genomics Study participants onto UK Biobank PCs (2-8)

Projection of UK COVID-19 Genomics Study participants (3rd degree and below relatives removed) onto UK Biobank genetic principal component axes, coloured by assigned genetic ancestry group. Participants from the UK COVID-19 Genomics Study are shown as points and were projected onto the principal component space derived from UK Biobank reference data, as described by Privé et al 2022[4]. Black outlined points with labels indicate the positions of 21 UK Biobank reference groups, projected onto the same axes using their allele frequency profiles. Reference groups merged for downstream analyses are labelled individually but coloured according to the broader merged ancestry group. Panels show PC2 versus PC3 (A), PC4 versus PC5 (B), PC6 versus PC7 (C), and PC8 versus PC9 (D).

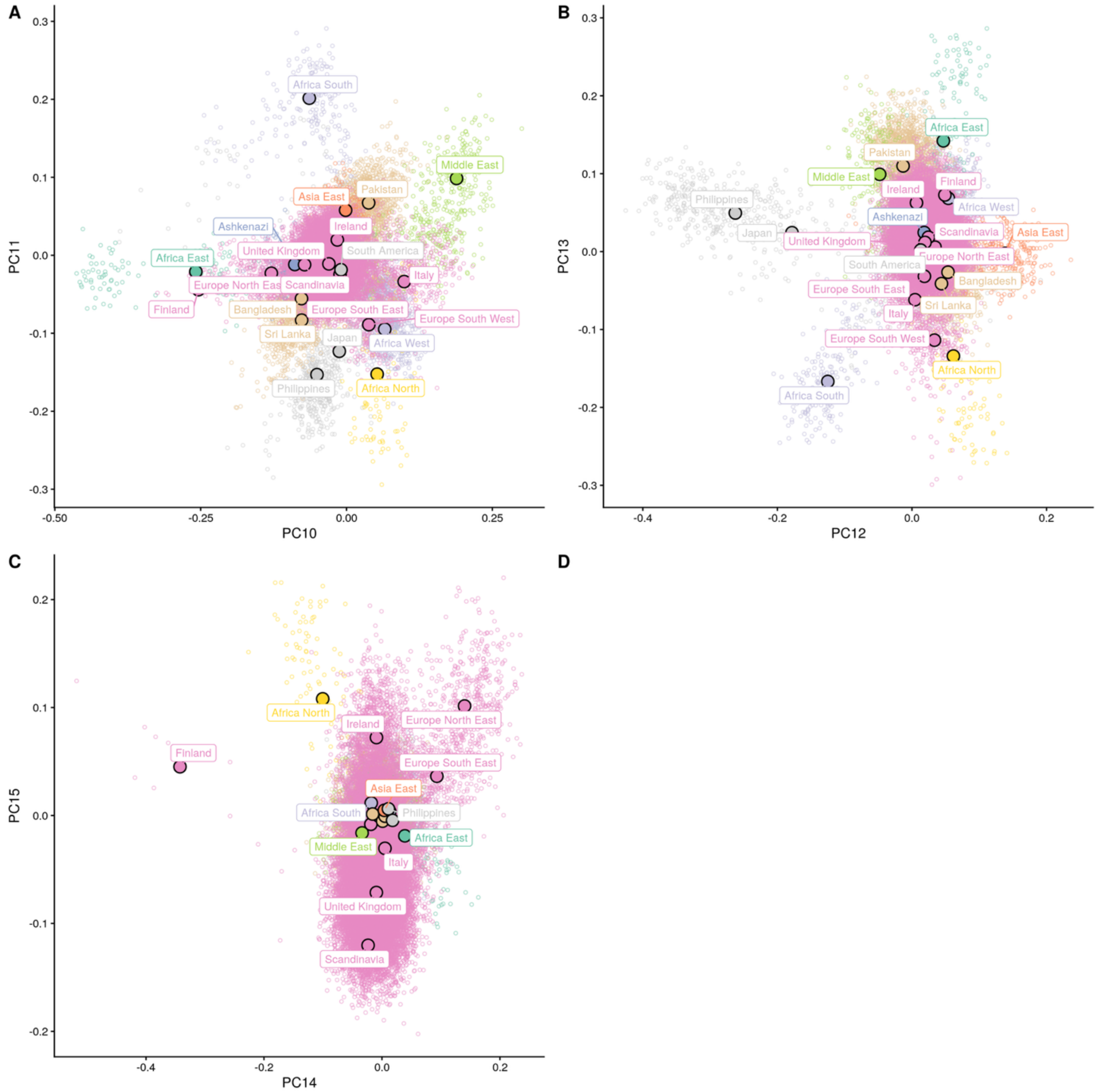

Supplementary Figure 13 - Projection of UK COVID-19 Genomics Study participants onto UK Biobank PCs (11-14)

Projection of **UK COVID-19 Genomics Study participants** (3rd degree and below relatives removed) onto UK Biobank genetic principal component axes, coloured by assigned genetic ancestry group. Participants from the UK COVID-19 Genomics Study are shown as points and were projected onto the principal component space derived from UK Biobank reference data, as described by Privé et al 2022[4]. Black outlined points with labels indicate the positions of 21 UK Biobank reference groups, projected onto the same axes using their allele frequency profiles. Reference groups merged for downstream analyses are labelled individually but coloured according to the broader merged ancestry group. Panels show PC10 versus PC11 (A), PC12 versus PC13 (B), and PC14 versus PC15 (C).

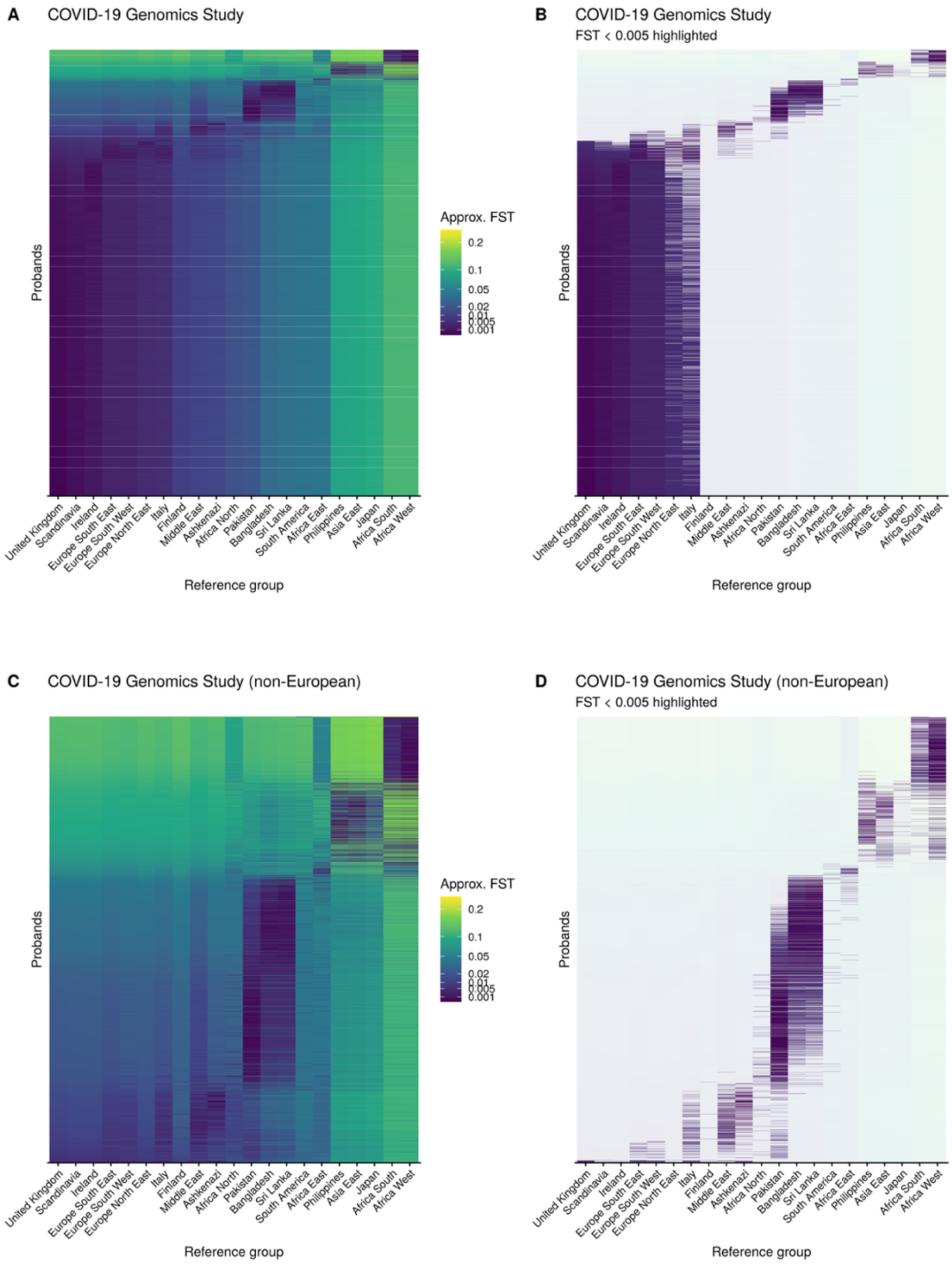

Supplementary Figure 14 - Approximate genetic distance between UK COVID-19 Genomics Study participants and UK Biobank reference groups

For each participant, distances to 21 UK Biobank reference groups were calculated as squared Euclidean distances in principal component space to reference group centres, normalised by the maximum pairwise distance between reference groups and scaled to approximate F_{ST}. Rows represent probands and columns represent reference groups, with both ordered by increasing distance to the United Kingdom reference group. (A) Heatmap of approximate distance values across all proband–reference group comparisons. (B) Same data with cells corresponding to values < 0.005 highlighted, representing the threshold used for assignment to one or more reference groups prior to group consolidation. (C/D) as in A/B but excluding participants assigned to the European ancestry group (following consolidation).

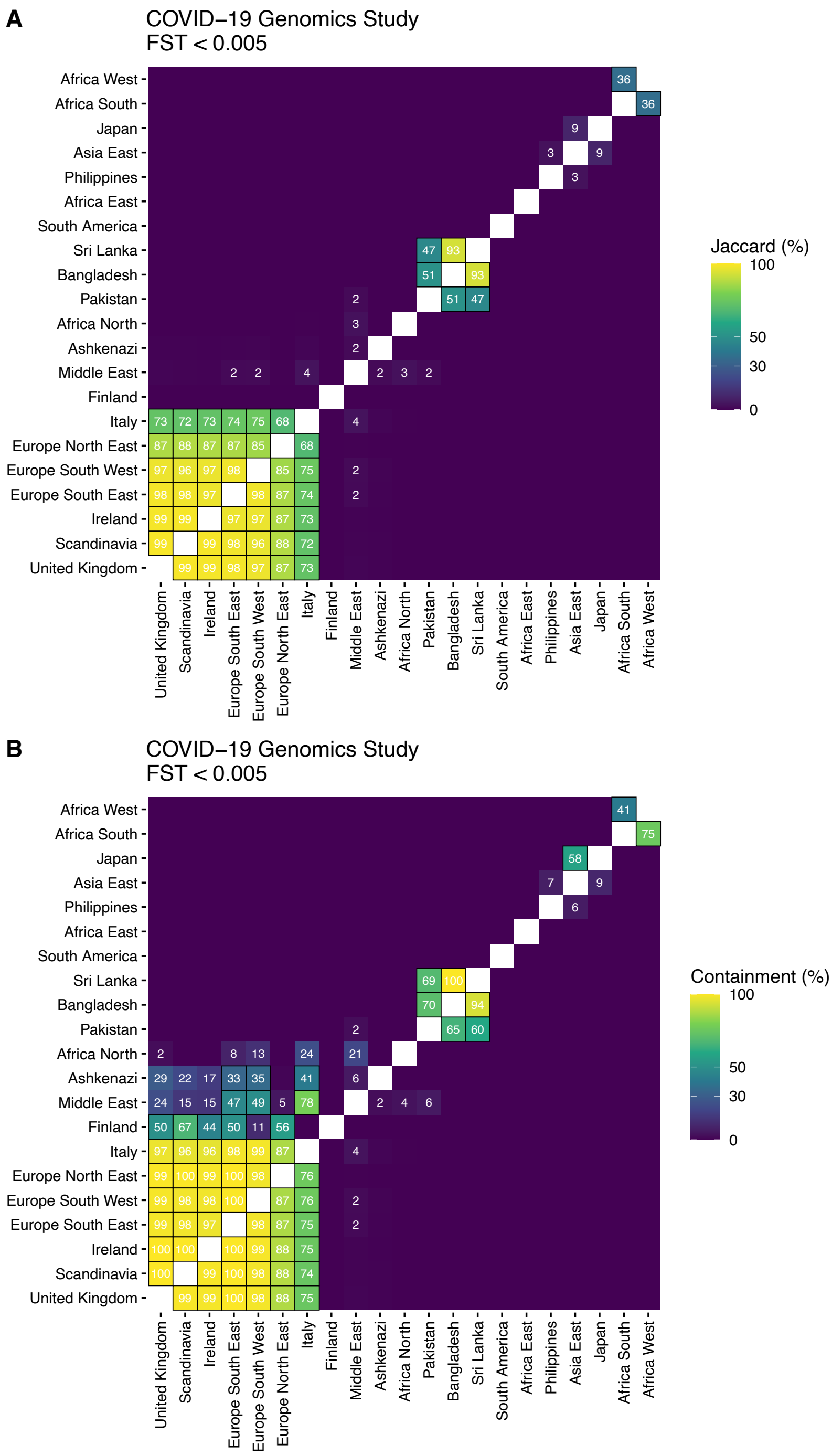

Supplementary Figure 15 - Pairwise overlap between UK Biobank reference groups among UK COVID-19 Genomics Study participants assigned to one or more groups at an approximate F_{ST} threshold <0.005, before consolidation into broader ancestry groups.

For each reference group, individuals with approximate $F_{ST} < 0.005$ were treated as a set. (A) Symmetric pairwise overlap measured using the Jaccard index, calculated as the percentage of individuals shared between two reference-group sets divided by the size of their union. (B) Directional pairwise containment, showing the percentage of individuals assigned to the reference group on the y-axis who were also assigned to the reference group on the x-axis.

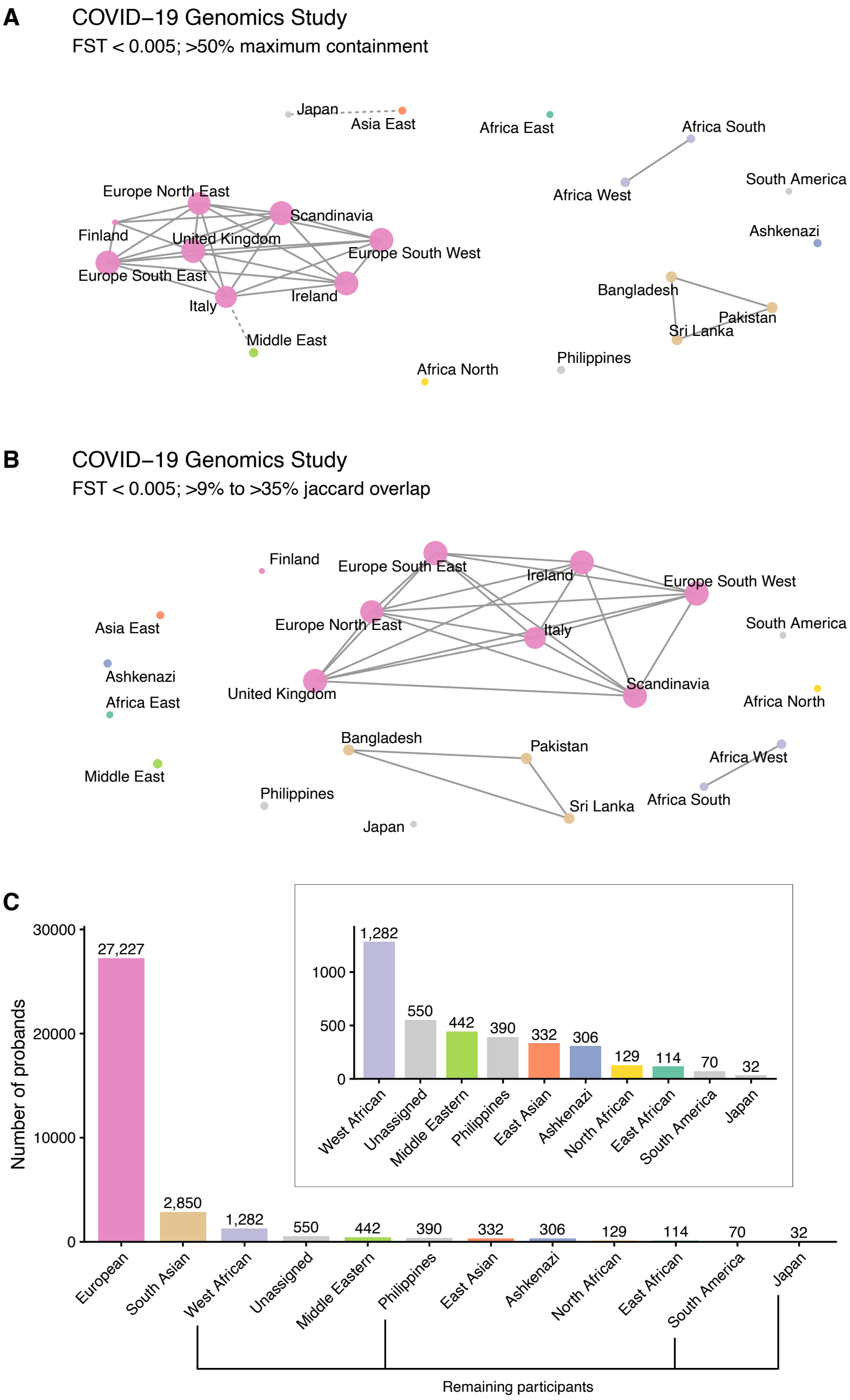

Supplementary Figure 16 - Graph-based consolidation of UK COVID-19 Genomic Study participants into broader ancestry groups

(A) Network constructed using a maximum containment threshold (>50%) between groups following assignment at $F_{ST} < 0.005$. Nodes represent groups, with node size proportional to the number of individuals assigned. Edges connect sub-groups exceeding the specified containment threshold (in either direction). Dashed edges denote connections involving the Middle East/Japanese groups, which are retained as separate groups due to low Jaccard overlap despite >50% containment. (B). Network constructed using pairwise Jaccard overlap thresholds (8%–36%) under the same $F_{ST} < 0.005$ assignment. Edges connect subpopulations exceeding the specified overlap thresholds. (C) Distribution of probands across 11 ancestry groups (and unassigned) following consolidation (merging of connected subgroups). Groups were combined into a single “Remaining participants” category to align with the procedure applied to the 100kGP rare disease probands.

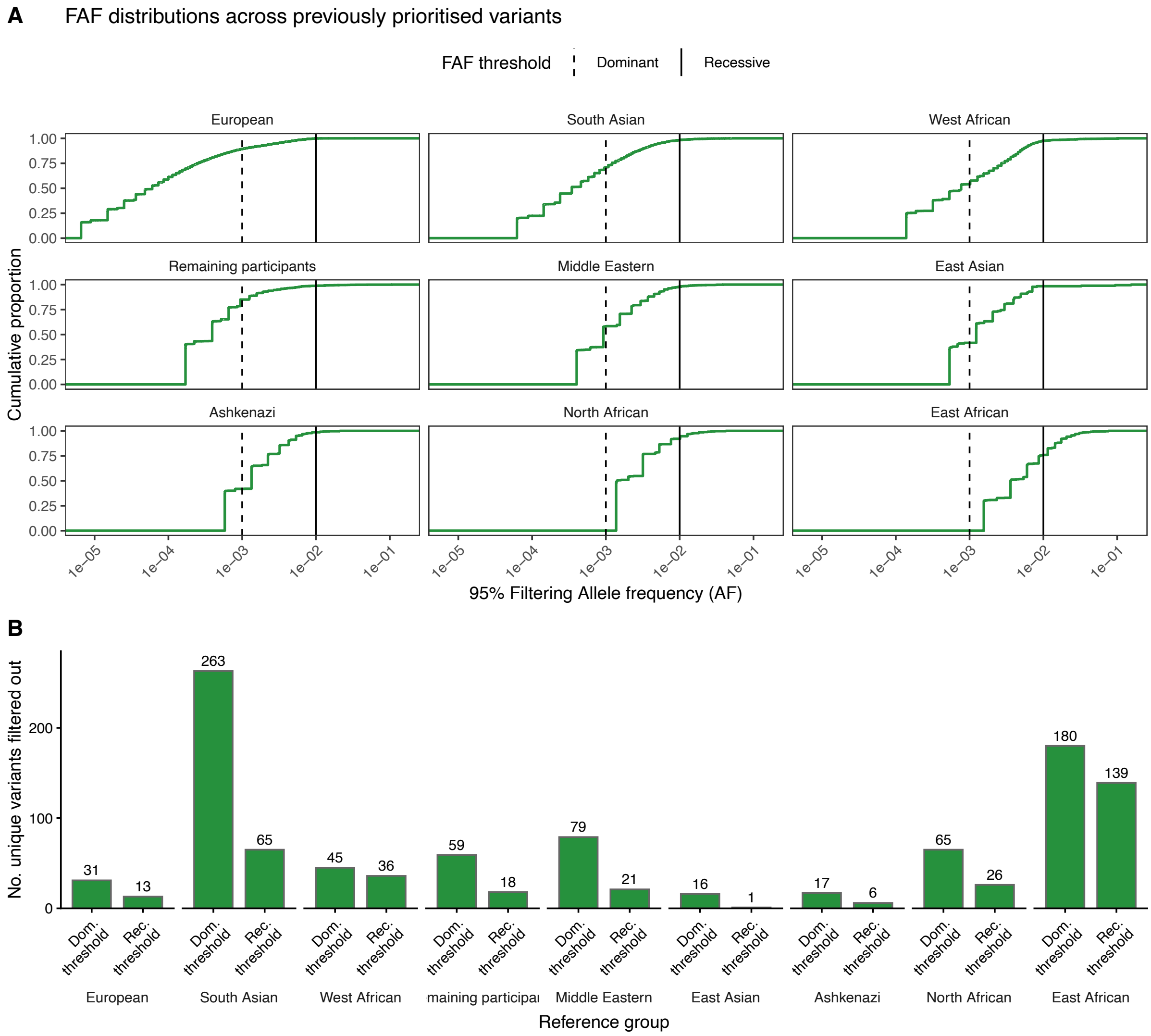

Supplementary Figure 17 – Filtering allele frequency (FAF) distributions and statistics from the application of UK COVID-19 Genomic Study reference panel to prioritised variants in the 100kGP

(A) Ancestry group specific FAF distributions from the UK COVID-19 Genomics Study reference panel for all previously prioritised variants in the 100 000 Genomes Project rare diseases cohort. FAF thresholds are shown for dominant and recessive inheritance models annotated by the pipeline (B) For each reference ancestry group, the number of (unique) prioritised variants that were filtered out in each of the reference ancestry groups at each threshold.

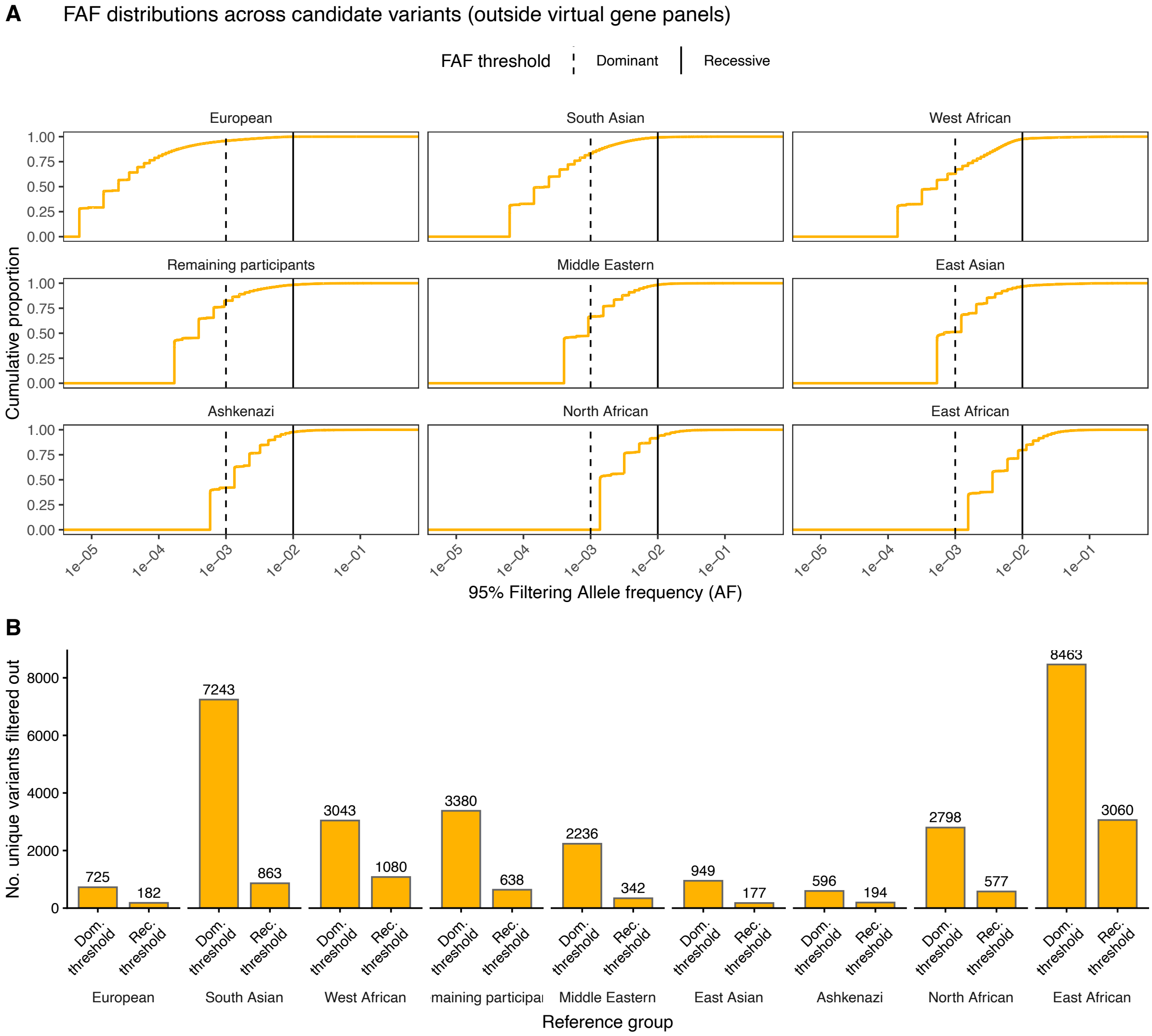

Supplementary Figure 18 - Filtering allele frequency (FAF) distributions and statistics from the application of UK COVID-19 Genomic Study reference panel to candidate variants (outside virtual gene panels) in the 100kGP

(A) Ancestry group specific FAF distributions from the UK COVID-19 Genomics Study reference panel for all previous candidate variants outside the virtual panels in the 100 000 Genomes Project rare diseases cohort. FAF thresholds are shown for dominant and recessive inheritance models annotated by the pipeline (B) For each reference ancestry group, the number of (unique) candidate variants outside panels that were filtered out in each of the reference ancestry groups at each threshold.

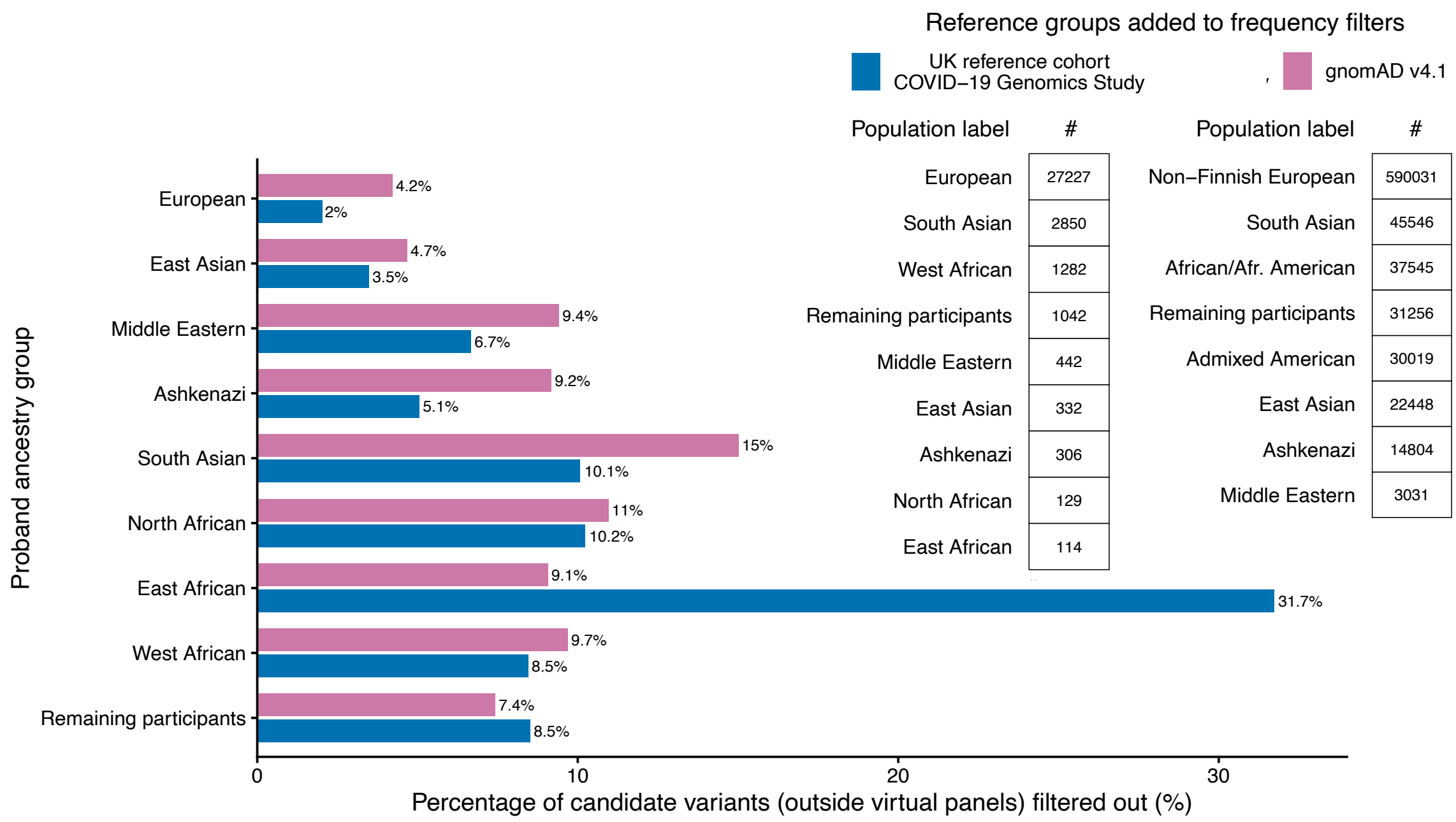

Supplementary Figure 19 - Impact of additional population frequency filters on candidate variants outside virtual panels across ancestry groups.

Blue bars show the percentage of candidate variants outside the applied panels removed for each ancestry group, when additional allele frequency thresholds estimated from the UK COVID-19 Genomics Study (blue) were added to standard pipeline allele frequency filters. Magenta bars show the same for allele frequencies derived from reference groups in gnomADv4.1 (magenta). Sample sizes for each labelled reference group are shown in the inset tables.

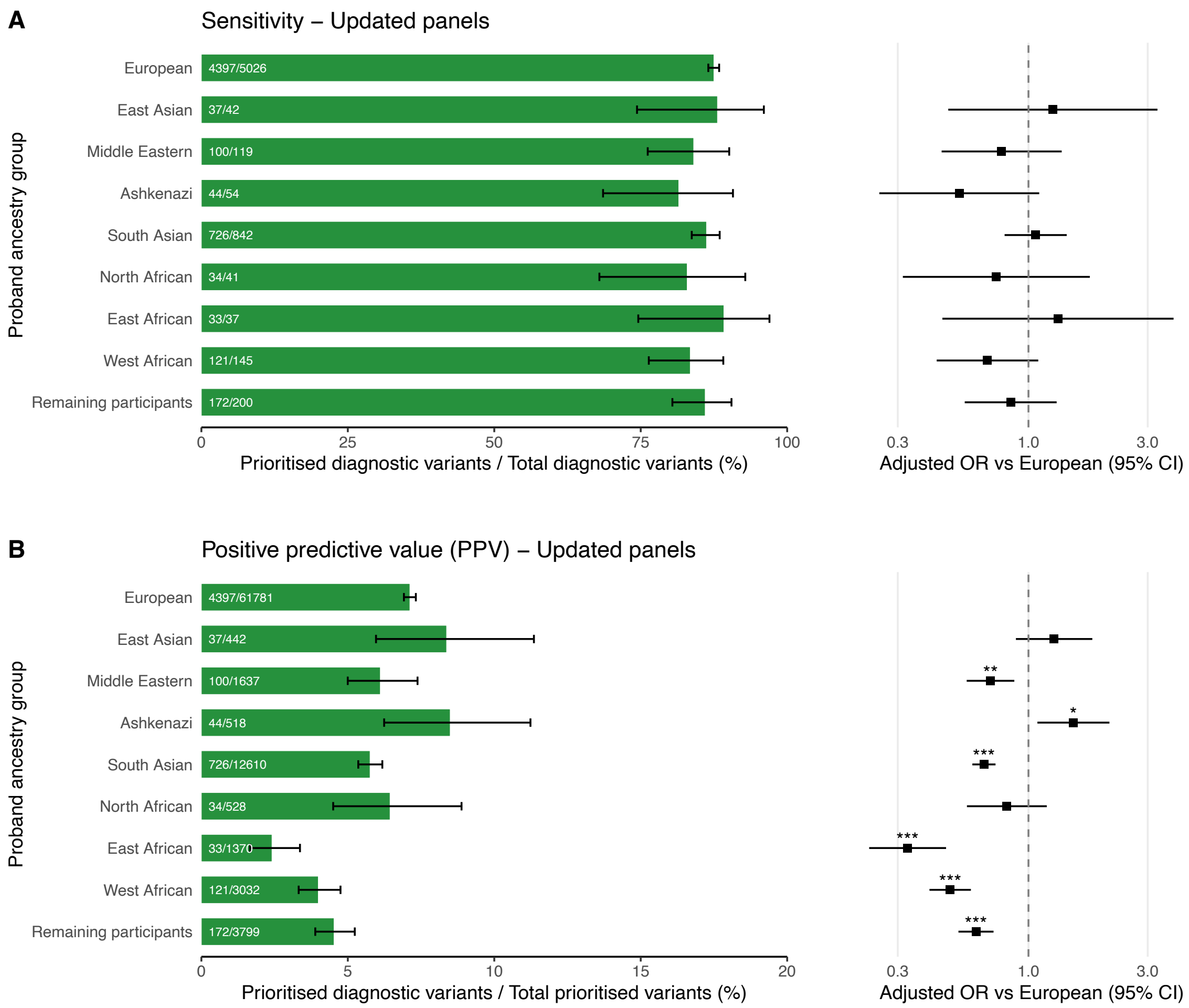

Supplementary Figure 20 - Sensitivity and positive predictive value of prioritised variants (using updated virtual gene panels), stratified by genetically inferred ancestry group.

(A) Left: proportion of reported diagnostic SNVs and indels intersecting with variants retrospectively prioritised using the updated virtual gene panels (sensitivity) shown as green bars with numerator/denominator labels inside each bar and 95% Clopper–Pearson CIs (whiskers). Right: adjusted-OR forest plot. (B) Left: proportion of these updated panel SNVs and indels reported as diagnostic (PPV) shown as green bars with numerator/denominator labels inside each bar and 95% Clopper–Pearson CIs (whiskers). Right: adjusted-OR forest plot. Estimates of adjusted OR vs European are from multivariable mixed-effects logistic regression models adjusted for age, sex, family structure, family-member affection status, and predicted consanguinity, with random intercepts for disease category and the handling GMC. *** p<0·001; ** p<0·01; * p<0·05).

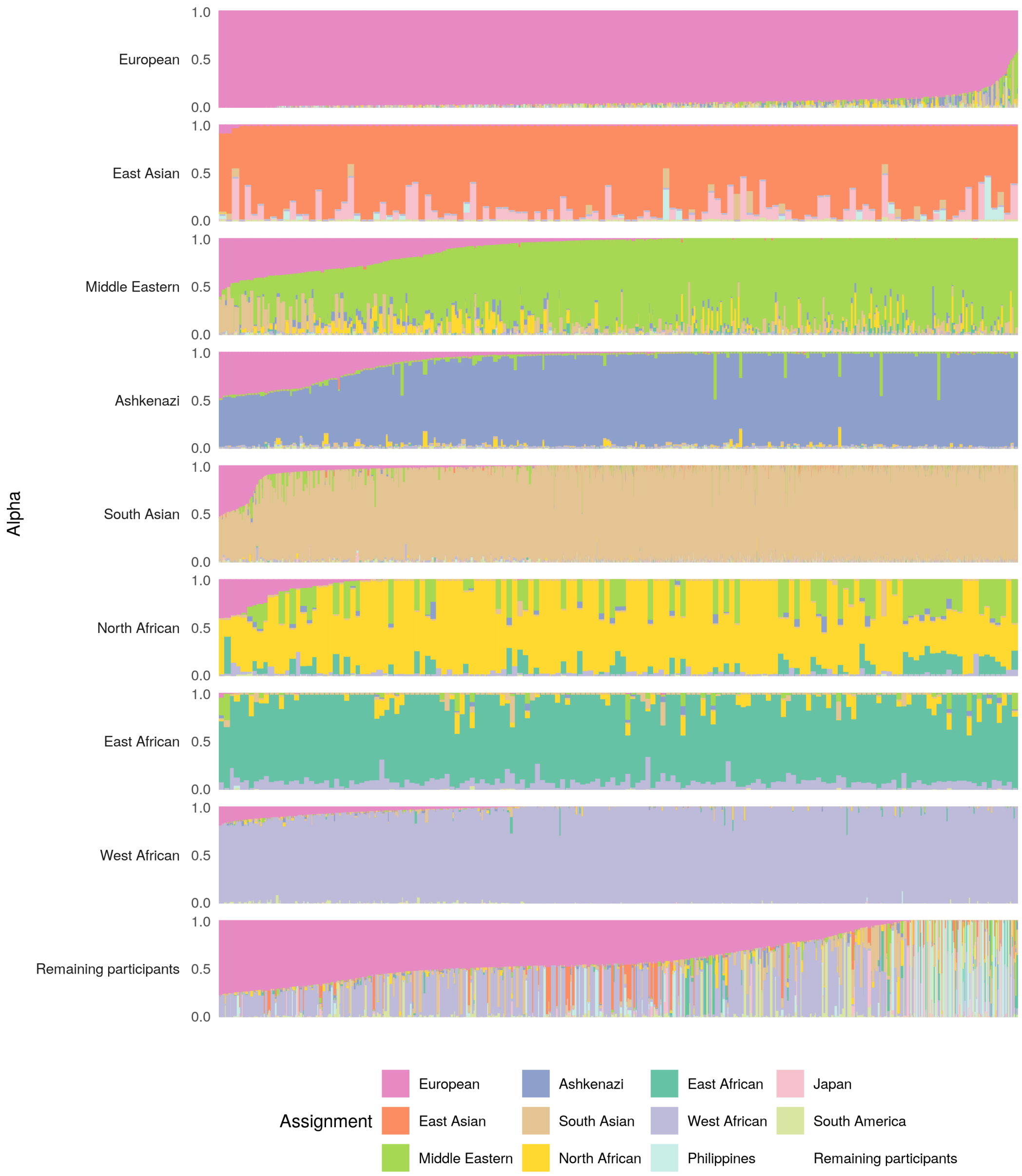

Supplementary Figure 21 - Convex ancestry proportions of 100kGP rare disease probands estimated using UK Biobank reference groups, stratified by assigned genetic ancestry group.

Ancestry proportions were estimated using a quadratic programming approach and aggregated across reference subgroups into the consolidated ancestry groups, including those smaller ancestry groups (Philippines, Japan, South America) that were merged into remaining participants for downstream analyses. Each bar represents one proband, ordered within panel by decreasing European ancestry proportion, with stacked colours indicating each participant's ancestry proportions across consolidated reference groups (as shown in the figure legend).

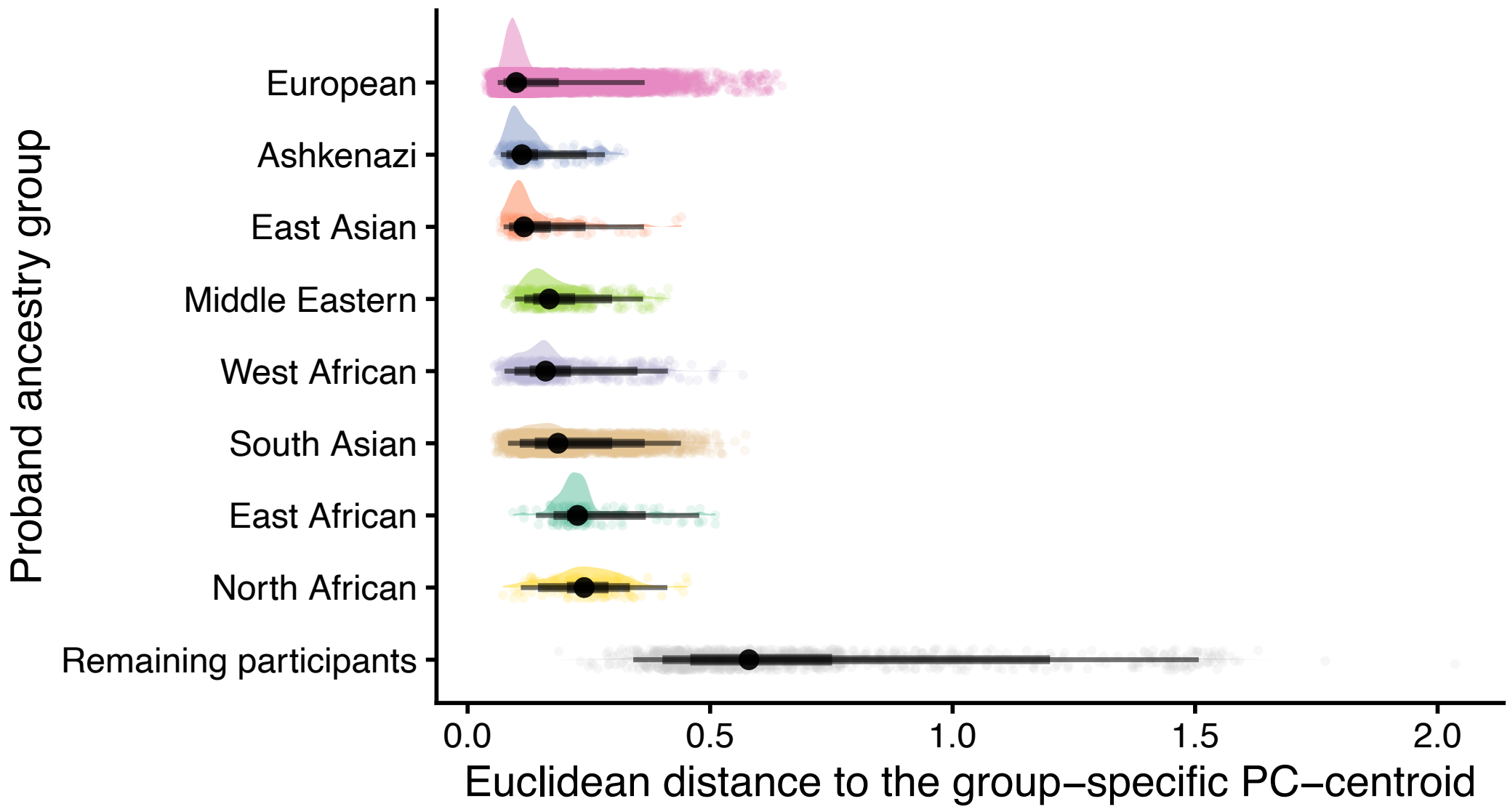

Supplementary Figure 22 - Distribution of within-group genetic heterogeneity across ancestry groups in the 100kGP rare disease cohort

For each proband, Euclidean distance in projected UK Biobank reference principal component (PC) space to the centroid of their assigned ancestry group was calculated. Points represent individual probands, and shaded densities show the distribution within each group. Black points and horizontal bars indicate group medians and interquartile ranges.

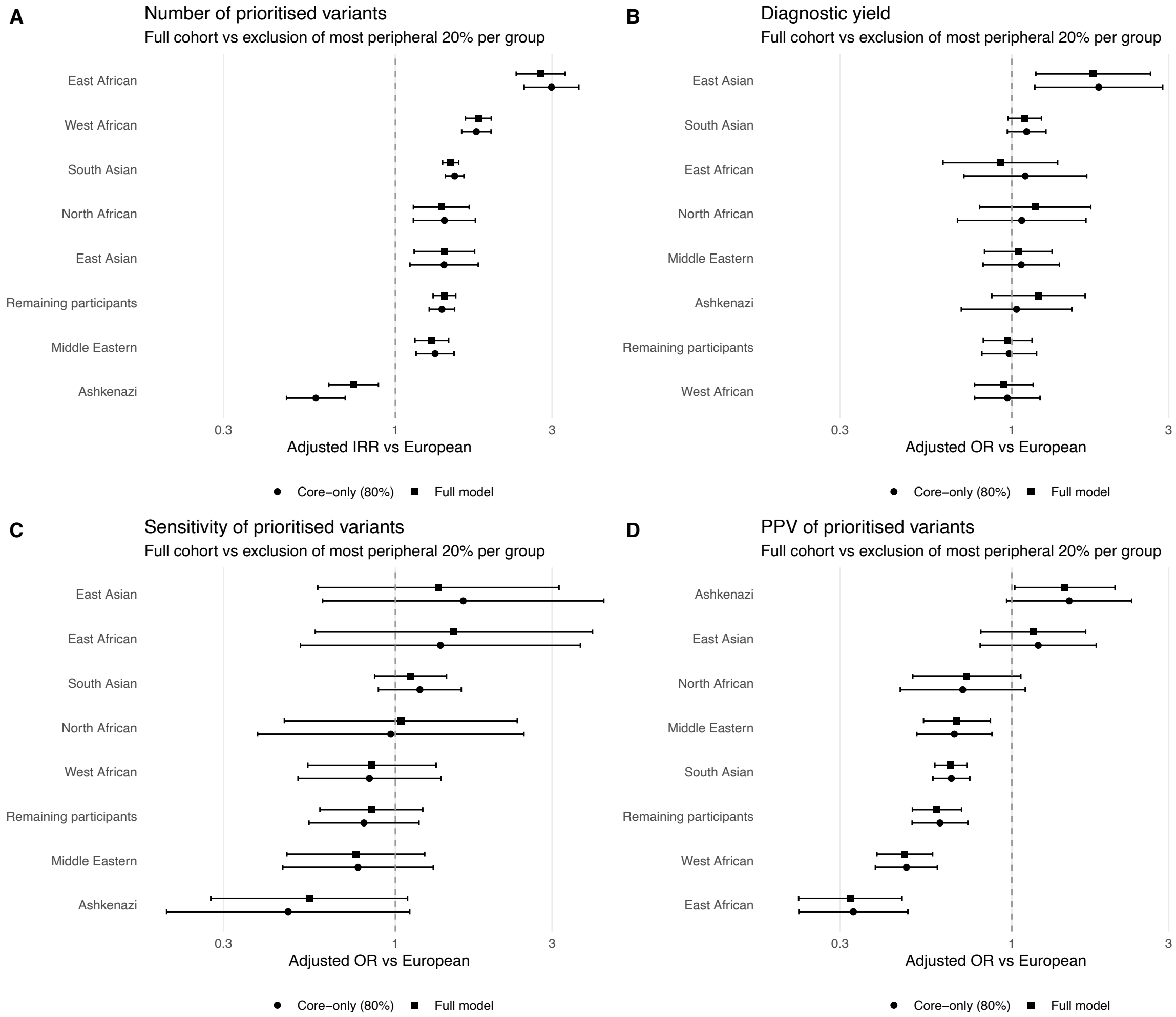

Supplementary Figure 23 - Adjusted incidence rate ratios (IRRs) and odds ratios (ORs) comparing genetically inferred ancestry groups with the European group under the full cohort and after exclusion of the most peripheral 20% of individuals within each ancestry group

Peripheral individuals were defined based on Euclidean distance to the group-specific principal component centroid (Supplementary Figure 22). Points and error bars represent adjusted estimates and 95% CIs from multivariable mixed-effects models, adjusted for age, sex, family structure, family-member affection status, and predicted consanguinity, with random intercepts for disease category and handling GMC. (A) Number of prioritised variants (IRR). (B) Diagnostic yield (OR). (C) Sensitivity of prioritised variants (OR). (D) Positive predictive value (PPV) of prioritised variants (OR).

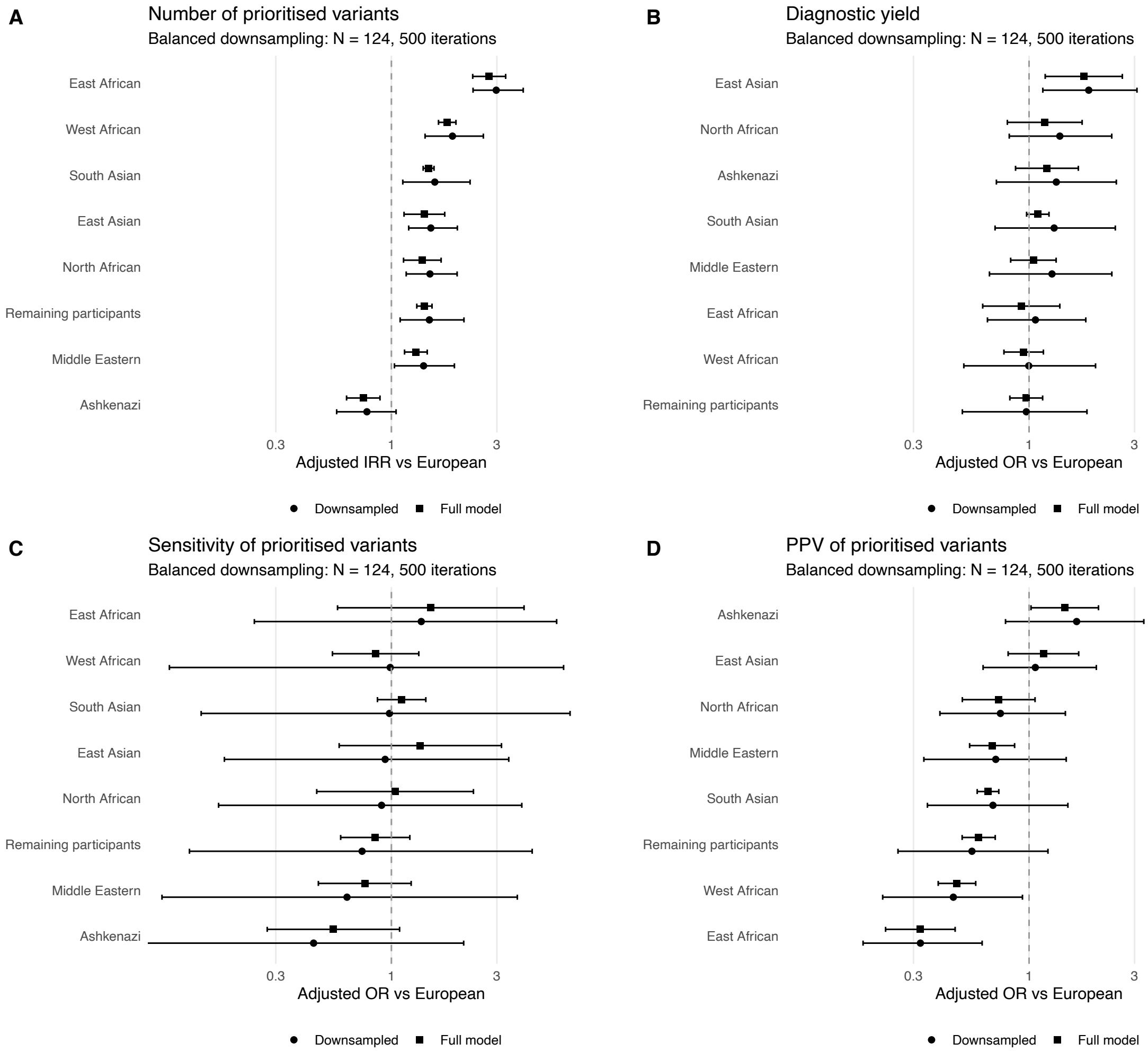

Supplementary Figure 24 - Adjusted incidence rate ratios (IRRs) and odds ratios (ORs) comparing genetically inferred ancestry groups with the European reference group in the full cohort and after balanced down sampling to 124 probands per ancestry group across 500 iterations.

Points and error bars for the down sampled analyses represent the mean estimate and empirical 95% interval across iterations; full-model estimates are shown for comparison. Estimates are from multivariable mixed-effects models adjusted for age, sex, family structure, family-member affection status, and predicted consanguinity, with random intercepts for disease category and GMC. (A) Number of prioritised variants (IRR). (B) Diagnostic yield (OR). (C) Sensitivity of prioritised variants (OR). (D) Positive predictive value (PPV) of prioritised variants (OR).

Supplementary References

1 National Genomic Research Library. 2017; published online Dec 21. DOI:10.6084/m9.figshare.4530893.v7.

2 Smedley D, Jacobsen JOB, Jager M, *et al.* Next-generation diagnostics and disease-gene discovery with the Exomiser. *Nat Protoc* 2015; **10**: 2004–15.

3 Bycroft C, Freeman C, Petkova D, *et al.* The UK Biobank resource with deep phenotyping and genomic data. *Nature* 2018; **562**: 203–9.

4 Privé F. Using the UK Biobank as a global reference of worldwide populations: application to measuring ancestry diversity from GWAS summary statistics. *Bioinformatics* 2022; **38**: 3477–80.

5 Karczewski KJ, Francioli LC, Tiao G, *et al.* The mutational constraint spectrum quantified from variation in 141,456 humans. *Nature* 2020; **581**: 434–43.

6 Auton A, Abecasis GR, Altshuler DM, *et al.* A global reference for human genetic variation. *Nature* 2015; **526**: 68–74.

7 Purcell S, Neale B, Todd-Brown K, *et al.* PLINK: A Tool Set for Whole-Genome Association and Population-Based Linkage Analyses. *Am J Hum Genet* 2007; **81**: 559–75.

8 Privé F, Aschard H, Ziyatdinov A, Blum MGB. Efficient analysis of large-scale genome-wide data with two R packages: bigstatsr and bigsnpr. *Bioinformatics* 2018; **34**: 2781–7.

9 Kousathanas A, Pairo-Castineira E, Rawlik K, *et al.* Whole-genome sequencing reveals host factors underlying critical COVID-19. *Nature* 2022; **607**: 97–103.

10 Manichaikul A, Mychaleckyj JC, Rich SS, Daly K, Sale M, Chen W-M. Robust relationship inference in genome-wide association studies. *Bioinformatics* 2010; **26**: 2867–73.

11 Whiffin N, Minikel E, Walsh R, *et al.* Using high-resolution variant frequencies to empower clinical genome interpretation. *Genet Med* 2017; **19**: 1151–8.

12 Landrum MJ, Lee JM, Benson M, *et al.* ClinVar: improving access to variant interpretations and supporting evidence. *Nucleic Acids Research* 2018; **46**: D1062–7.

13 Chen S, Francioli LC, Goodrich JK, *et al.* A genomic mutational constraint map using variation in 76,156 human genomes. *Nature* 2024; **625**: 92–100.

14 Coop G. Genetic similarity versus genetic ancestry groups as sample descriptors in human genetics. 2023; published online Jan 7. DOI:10.48550/arXiv.2207.11595.

15 Lewis ACF, Molina SJ, Appelbaum PS, *et al.* Getting genetic ancestry right for science and society. *Science* 2022; **376**: 250–2.

Supplementary Materials
